# Non‐surgical treatment for lower limb apophyseal injuries

**DOI:** 10.1002/14651858.CD015156.pub2

**Published:** 2026-07-15

**Authors:** Cylie M Williams, Kasper Krommes, Kade L Paterson, Terry Haines, Antoni Caserta, Kristian Thorborg

**Affiliations:** School of Primary and Allied Health CareMonash UniversityFrankstonAustralia; Sports Orthopedic Research Center - Copenhagen (SORC-C), Department of Orthopedic SurgeryAmager-Hvidovre University HospitalCopenhagenDenmark; Centre for Health, Exercise and Sports Medicine, Department of PhysiotherapyThe University of MelbourneMelbourneAustralia; National Centre for Healthy AgeingMonash UniversityFrankstonAustralia; Department of Clinical MedicineUniversity of CopenhagenCopenhagenDenmark

## Abstract

**Rationale:**

Lower limb apophyseal injuries are common in children and adolescents. The most common are traction apophysitis of the tibial tubercle and calcaneal apophysis. Various treatments are used for apophyseal conditions. This review provides information for health professionals and families who are deciding on treatment.

**Objectives:**

To assess the benefits and harms of non‐surgical treatment versus placebo, no treatment, or another treatment on overall pain, physical function, or participation in physical activity in children and adolescents with lower limb apophyseal injuries.

**Search methods:**

We searched the following databases with no language restrictions up to 4 January 2025: Cochrane Central Register of Controlled Trials (CENTRAL; 2025, Issue 1) via Ovid, MEDLINE Ovid, Embase Ovid, CINAHL Plus, ClinicalTrials.gov (clinicaltrials.gov), and World Health Organization's International Clinical Trials Registry Platform (ICTRP) (www.who.int/ictrp/en/).

**Eligibility criteria:**

We searched for randomised controlled trials (RCTs) reported in full text, abstract form, or as unpublished data.

**Outcomes:**

Our critical outcomes were overall pain, physical function, participation in sport, withdrawals due to adverse events, and serious adverse events. The primary time point was up to three months for overall pain, physical function, and participation in sport (measured in days), and the end of the trial period for adverse event outcomes.

**Risk of bias:**

We assessed the risk of bias (RoB) in the findings using the Cochrane tool RoB 2.

**Synthesis methods:**

We calculated the standardised mean difference (SMD) or mean difference (MD) for continuous outcomes. We used the random‐effects model to combine data and quantified heterogeneity using the I² statistic. When we were unable to pool data, we described results narratively. We assessed the certainty of the evidence using GRADE.

**Included studies:**

We included 10 RCTs, seven on calcaneal apophysitis and three on traction apophysitis of the tibial tubercle. The studies involved 654 children, whose mean age was 10.3 to 13.3 years. Most of the participants were male (73%). Six studies compared intervention versus placebo or no treatment (or both), and five studies compared one intervention versus another. In the studies reporting our critical outcomes, there were five intervention groups: pharmaceutical interventions (e.g. dexamethasone), taping, foot orthoses/heel straps, heel lifts, and heel cushioning. Placebo interventions were lidocaine injections, saline via iontophoresis, or non‐stretch tape. 'Usual care' comparators were poorly described but included exercise, stretches, nonsteroidal anti‐inflammatories, and massage.

**Synthesis of results:**

**Pharmaceutical intervention versus placebo for children with traction apophysitis of the tibial tubercle**

Compared to placebo, the evidence is very uncertain about the effects of dexamethasone on overall pain (MD −0.52, 95% CI −1.24 to 0.20; 1 study, 23 participants; very low‐certainty evidence), physical function (MD −1.76, 95% CI −16.08 to 12.56; 1 study, 19 participants; very low‐certainty evidence), and participation in sport (MD 7.90, 95% CI −0.41 to 16.21; 1 study, 16 participants; very low‐certainty evidence) in the short term. The evidence is very uncertain about adverse events in the studies of dexamethasone or dextrose versus placebo (RR 1.31, 95% CI 0.88 to 1.96; 2 studies, 74 participants; very low‐certainty evidence). Withdrawals due to adverse events were not measured.

**Pharmaceutical intervention versus usual care for children with traction apophysitis of the tibial tubercle**

Compared to usual care, the evidence is very uncertain about the effects of dexamethasone on overall pain (MD −0.80, 95% CI −1.73 to 0.13; 1 study, 21 participants; very low‐certainty evidence), physical function (MD 2.68, 95% CI −17.56 to 22.92; 1 study, 16 participants; very low‐certainty evidence), and participation in sport (MD 0.85, 95% CI −7.13 to 8.83; 1 study, 11 participants; very low‐certainty evidence) in the short term. The evidence is very uncertain about adverse events in the study of dexamethasone versus usual care (RR 1.36, 95% CI 0.88 to 2.10; 1 study, 30 participants; very low‐certainty evidence). Withdrawals due to adverse events were not measured.

**Taping versus placebo for children with calcaneal apophysitis**

Compared to placebo, the evidence is very uncertain about the effects of Kinesio tape on overall pain (MD 0.10, 95% CI −1.25 to 1.45; 1 study, 22 participants; very low‐certainty evidence) and physical function (MD 6.10, 95% CI −0.08 to 12.28; 1 study, 22 participants; very low‐certainty evidence) in the short term. Participation in sport, adverse events, and withdrawals due to adverse events were not measured.

**Foot orthoses versus heel lifts for children with calcaneal apophysitis**

Compared to heel lifts, foot orthoses likely result in little to no difference in overall pain (MD 0.00, 95% CI −0.44 to 0.44; 1 study, 123 participants; moderate‐certainty evidence) or physical function (MD −1.30, 95% CI −7.58 to 4.98; 1 study, 124 participants; moderate‐certainty evidence) in the short term. There were no adverse events reported (11 studies, 101 participants; moderate‐certainty evidence). Participation in sport and withdrawals due to adverse events were not measured.

**Heel cushioning versus heel straps for children with calcaneal apophysitis**

Compared to a heel strap, the evidence is very uncertain about the effect of heel cushioning on physical function in the short term (MD −2.00, 95% CI −12.48 to 8.48; 1 study, 43 participants; very low‐certainty evidence) and on adverse events (RR 1.05, 95% CI 0.07 to 15.69; 1 study, 43 participants; very low‐certainty evidence). Overall pain, participation in sport, and withdrawals due to adverse events were not measured.

**Certainty of the evidence**

We downgraded our certainty level for most of the evidence because of risk of bias, imprecision, and possible publication bias.

**Authors' conclusions:**

Evidence for non‐surgical treatment of lower limb apophyseal injuries is limited. We rated it mostly low to very low certainty. The studies included in this review had heterogeneous outcomes, which restricted meaningful synthesis. Outcomes were primarily focused on pain, physical function, or activity participation, and the studies did not specifically target children who had persistent symptoms of apophysitis causing functional limitations. None of the trials measured quality of life, even though cohort studies have previously reported that apophyseal injuries can impact this long‐term. Nor did the trials examine economic impacts, despite the costs of non‐surgical treatments for apophyseal conditions to families and healthcare systems.

**Funding:**

None

**Registration:**

Protocol DOI: https://doi.org/10.1002/14651858.CD015156

## Plain language summary

**What are the effective treatments for growth‐plate pain (apophysitis) in hips, legs, or feet?**

**Key messages**

- Most children with growth‐plate pain (apophysitis) get better over time. Whether treatment for apophysitis affects short‐term pain, physical function, and participation in sport is uncertain.
- It is uncertain whether any treatment is better or worse than any other. The studies were mostly small and had problems with their design. The studies did not always measure unwanted effects from a treatment, and none measured whether any children withdrew from studies because of unwanted effects.
- The evidence is limited. Most studies included more boys than girls, and included children who were more active than average. The children often knew which treatment they were receiving, which may have influenced the results.

**What is apophysitis?**

During puberty, children sometimes develop pain in the growth plates in their hips, knees, and feet. This pain is usually caused by irritation of the growth plate from repeated stress over time rather than from a single event. The pain is usually short‐term and has limited impact on a child's life. However, sometimes the pain can make children limp, play less sport, or be less physically active. The conditions are called different names based on the location of the growth, but are commonly known as 'apophysitis' or 'lower limb apophyseal injuries'.

**How is apophysitis treated?**

Many different healthcare professionals treat apophysitis, including general practitioners or physicians, physiotherapists, and podiatrists. Many treatments are used, including exercises, medicines, strapping, braces, or devices put in shoes like foot orthoses or heel lifts. Regardless of treatment, apophysitis usually gets better on its own, but in some cases, the pain can last for a long time.

**What did we want to find out?**

We wanted to find out which treatments reduce pain effectively and to understand how safe these treatments are. We wanted to know which treatments improve how children function physically and take part in sport. We also wanted to know if one treatment worked better than another.

**What did we do?**

We searched for studies that tested the different types of treatments for apophysitis in the hip, knee, and foot. We did not search for any treatments that involved surgery.

We combined results from studies that tested the same treatments and used similar methods to measure effectiveness. We rated our level of certainty about this evidence.

**What did we find?**

We found 10 studies that involved 654 children whose average age ranged from 10.3 to 13.3 years. Seven studies focused on heel pain (calcaneal apophysitis), and three focused on pain in the front of the knee (traction apophysitis of the tibial tubercle).

**Main results**

- One study (23 children) compared a medicine called dexamethasone to a placebo (fake treatment). We are very uncertain whether dexamethasone reduces pain, improves physical function, or helps children return to sport in the short term. Two studies (74 children) reported unwanted effects of medicines, but the evidence was also very uncertain.
- One study (21 children) compared dexamethasone to usual care. We are very uncertain whether dexamethasone reduces pain, improves physical function, or helps children return to sport in the short term. One study (30 children) reported unwanted effects, but the evidence was also very uncertain.
- One study (22 children) compared Kinesio tape to a placebo. We are very uncertain whether Kinesio tape improves pain or physical function in the short term. The study did not report on participation in sport or unwanted effects.
- One study (124 children) compared foot orthoses to heel lifts. It found that there is probably little to no difference between them in terms of pain or physical function in the short term. The study did not report on participation in sport. One study (101 children) reported that there were no unwanted effects.
- One study (43 children) compared heel cushioning to a heel strap. We are very uncertain whether heel cushioning improves physical function or causes any unwanted effects in the short term. The study did not report on participation in sport.
- None of the studies reported whether any children withdrew from the studies because of unwanted effects.

**What are the limitations of this research?**

- In most studies, the children and carers knew which treatments they were receiving, which may have influenced how they reported improvements. This may affect how reliable the results are.
- Apophysitis often improves on its own as children continue to grow and the growth‐plates close, so it is difficult to know if any improvements are due to treatments or natural recovery over time.
- There were many more boys than girls in the studies, and most participants were very physically active. It is unlikely that the condition impacts boys and girls differently, but we are unsure if our findings can be applied to children who are less physically active.
- Finally, most studies had only a small number of participants. This means we cannot be confident in the results and cannot know whether the findings would apply to a wider group of children with apophysitis.

**How up to date is this evidence?**

The evidence is current to 4 January 2025.

## Summary of findings

**Summary of findings 1:** Summary of findings table ‐ A pharmaceutical intervention compared to placebo for children with traction apophysitis of the tibial tubercle

**A pharmaceutical intervention compared to placebo for children with traction apophysitis of the tibial tubercle**						
**Patient or population:** children with traction apophysitis of the tibial tubercle **Setting:** Tertiary care **Intervention:** a pharmaceutical intervention **Comparison:** placebo						
Outcomes	Anticipated absolute effects^*^ (95% CI)		Relative effect (95% CI)	№ of participants (studies)	Certainty of the evidence (GRADE)	Comments
	Risk with placebo	Risk with a pharmaceutical intervention				
Overall pain assessed with: VAS (Lower = less pain) Scale from: 0 to 10 follow‐up: 8 weeks	The mean overall pain was **1.85** points	MD **0.52 points lower** (1.24 lower to 0.2 higher)	‐	23 (1 RCT)	⊕⊝⊝⊝ Very low^a,^^b,^^c^	The evidence is very uncertain about the effect of a pharmaceutical intervention on overall pain in the short term.
Physical function assessed with: LEFS (Higher = greater function) Scale from: 0 to 100 follow‐up: 8 weeks	The mean physical function was **86.42** points	MD **1.76 points lower** (16.08 lower to 12.56 higher)	‐	19 (1 RCT)	⊕⊝⊝⊝ Very low^a,^^b,^^c^	The evidence is very uncertain about the effect of a pharmaceutical intervention on physical function in the short term.
Participation in sports or physical activity assessed with: Days to return to sport (Lower = quicker) Scale from: 0 to 56 follow‐up: 8 weeks	The mean participation in sports or physical activity was **30.2** days	MD **7.9 days higher** (0.41 lower to 16.21 higher)	‐	16 (1 RCT)	⊕⊝⊝⊝ Very low^a,^^b,^^c^	The evidence is very uncertain about the effect of a pharmaceutical intervention on participation in sport in the short term.
Withdrawal due to adverse events ‐ not reported	‐	‐	‐	‐	‐	
Adverse events assessed with: Count follow‐up: range 8 weeks to 12 weeks	278 per 1000	**364 per 1000** (244 to 544)	**RR 1.31** (0.88 to 1.96)	74 (2 RCTs)	⊕⊝⊝⊝ Very low^a,^^b,^^c^	The evidence is very uncertain about the adverse effects of pharmaceutical interventions.
*The risk in the intervention group (and its 95% confidence interval) is based on the assumed risk in the comparison group and the **relative effect** of the intervention (and its 95% CI). **CI:** confidence interval; **MD:** mean difference; **RR:** risk ratio						
**GRADE Working Group grades of evidence** **High certainty:** we are very confident that the true effect lies close to that of the estimate of the effect. **Moderate certainty:** we are moderately confident in the effect estimate: the true effect is likely to be close to the estimate of the effect, but there is a possibility that it is substantially different. **Low certainty:** our confidence in the effect estimate is limited: the true effect may be substantially different from the estimate of the effect. **Very low certainty:** we have very little confidence in the effect estimate: the true effect is likely to be substantially different from the estimate of effect.						
See interactive version of this table: https://gdt.gradepro.org/presentations/#/isof/isof_question_revman_web_464807522770217534.						

^a^ We downgraded twice for risk of bias as single study had a high risk of bias ^b^ We downgraded twice for imprecision as sample size was not reached and trial ceased early ^c^ We downgraded for publication bias as only limited results were available from online trial registry without information on adherence to protocol

**Summary of findings 2:** Summary of findings table ‐ A pharmaceutical intervention compared to usual care for children with traction apophysitis of the tibial tubercle

**A pharmaceutical intervention compared to usual care for children with traction apophysitis of the tibial tubercle**						
**Patient or population:** children with traction apophysitis of the tibial tubercle **Setting:** Tertiary care **Intervention:** a pharmaceutical intervention **Comparison:** usual care						
Outcomes	Anticipated absolute effects^*^ (95% CI)		Relative effect (95% CI)	№ of participants (studies)	Certainty of the evidence (GRADE)	Comments
	Risk with usual care	Risk with a pharmaceutical intervention				
Overall pain assessed with: VAS (Lower = less pain) Scale from: 0 to 10 follow‐up: 8 weeks	The mean overall pain was **2.13** points	MD **0.8 points lower** (1.73 lower to 0.13 higher)	‐	21 (1 RCT)	⊕⊝⊝⊝ Very low^a,^^b,^^c^	The evidence is very uncertain about the effect of a dexamethasone on overall pain in the short term.
Physical function assessed with: LEFS (Higher = greater function) Scale from: 0 to 100 follow‐up: 8 weeks	The mean physical function was **81.98** points	MD **2.68 points higher** (17.56 lower to 22.92 higher)	‐	16 (1 RCT)	⊕⊝⊝⊝ Very low^a,^^b,^^c^	The evidence is very uncertain about the effect of a dexamethasone on physical function in the short term
Participation in sports or physical activity assessed with: Days to return to sport (Lower = quicker) Scale from: 0 to 56 follow‐up: 8 weeks	The mean participation in sports or physical activity was **37.25** days	MD **0.85 days higher** (7.13 lower to 8.83 higher)	‐	11 (1 RCT)	⊕⊝⊝⊝ Very low^a,^^b,^^c^	The evidence is very uncertain about the effect of a dexamethasone on participation in sport in the short term.
Withdrawal due to adverse events ‐ not reported	‐	‐	‐	‐	‐	
Adverse events assessed with: Count follow‐up: 8 weeks	643 per 1000	**874 per 1000** (566 to 1000)	**RR 1.36** (0.88 to 2.10)	30 (1 RCT)	⊕⊝⊝⊝ Very low^a,^^b,^^c^	The evidence is very uncertain about the adverse effects of dexamethasone.
*The risk in the intervention group (and its 95% confidence interval) is based on the assumed risk in the comparison group and the **relative effect** of the intervention (and its 95% CI). **CI:** confidence interval; **MD:** mean difference; **RR:** risk ratio						
**GRADE Working Group grades of evidence** **High certainty:** we are very confident that the true effect lies close to that of the estimate of the effect. **Moderate certainty:** we are moderately confident in the effect estimate: the true effect is likely to be close to the estimate of the effect, but there is a possibility that it is substantially different. **Low certainty:** our confidence in the effect estimate is limited: the true effect may be substantially different from the estimate of the effect. **Very low certainty:** we have very little confidence in the effect estimate: the true effect is likely to be substantially different from the estimate of effect.						
See interactive version of this table: https://gdt.gradepro.org/presentations/#/isof/isof_question_revman_web_464808353006742408.						

^a^ We downgraded twice for risk of bias as single study had a high risk of bias ^b^ We downgraded twice for imprecision as as sample size was not reached and trial ceased early ^c^ We downgraded for publication bias as only limited results were available from online trial registry without information on adherence to protocol

**Summary of findings 3:** Summary of findings table ‐ Taping compared to placebo for children with calcaneal apophysitis

**Taping compared to placebo for children with calcaneal apophysitis**						
**Patient or population:** children with calcaneal apophysitis **Setting:** Tertiary care **Intervention:** taping **Comparison:** placebo						
Outcomes	Anticipated absolute effects^*^ (95% CI)		Relative effect (95% CI)	№ of participants (studies)	Certainty of the evidence (GRADE)	Comments
	Risk with placebo	Risk with taping				
Overall pain assessed with: VAS (Lower = less pain) Scale from: 0 to 10 follow‐up: 1 weeks	The mean overall pain was **5.4** points	MD **0.1 points higher** (1.25 lower to 1.45 higher)	‐	22 (1 RCT)	⊕⊝⊝⊝ Very low^a,^^b^	The evidence is very uncertain about the effect of taping on overall pain in the short term.
Physical function assessed with: AOFAS (Higher = greater function Scale from: 0 to 100 follow‐up: 1 weeks	The mean physical function was **77.4** points	MD **6.1 points higher** (0.08 lower to 12.28 higher)	‐	22 (1 RCT)	⊕⊝⊝⊝ Very low^a,^^b^	The evidence is very uncertain about the effect of taping on physical function in the short term.
Participation in sports or physical activity ‐ not measured	‐	‐	‐	‐	‐	
Withdrawal due to adverse events ‐ not measured	‐	‐	‐	‐	‐	
Adverse events ‐ not measured	‐	‐	‐	‐	‐	
*The risk in the intervention group (and its 95% confidence interval) is based on the assumed risk in the comparison group and the **relative effect** of the intervention (and its 95% CI). **CI:** confidence interval; **MD:** mean difference						
**GRADE Working Group grades of evidence** **High certainty:** we are very confident that the true effect lies close to that of the estimate of the effect. **Moderate certainty:** we are moderately confident in the effect estimate: the true effect is likely to be close to the estimate of the effect, but there is a possibility that it is substantially different. **Low certainty:** our confidence in the effect estimate is limited: the true effect may be substantially different from the estimate of the effect. **Very low certainty:** we have very little confidence in the effect estimate: the true effect is likely to be substantially different from the estimate of effect.						
See interactive version of this table: https://gdt.gradepro.org/presentations/#/isof/isof_question_revman_web_464808140165475104.						

^a^ We downgraded twice for risk of bias as single study had a high risk of bias ^b^ We downgraded twice for imprecision due to very small participant numbers

**Summary of findings 4:** Summary of findings table ‐ Foot orthoses compared to heel lifts for children with calcaneal apophysitis

**Foot orthoses compared to heel lifts for children with calcaneal apophysitis**						
**Patient or population:** children with calcaneal apophysitis **Setting:** Tertiary care **Intervention:** foot orthoses **Comparison:** heel lifts						
Outcomes	Anticipated absolute effects^*^ (95% CI)		Relative effect (95% CI)	№ of participants (studies)	Certainty of the evidence (GRADE)	Comments
	Risk with heel lifts	Risk with foot orthoses				
Overall pain assessed with: Faces pain scale ( Lower = less pain) Scale from: 0 to 6 follow‐up: 4 weeks	The mean overall pain was **2.9** points	MD **0 points** (0.44 lower to 0.44 higher)	‐	123 (1 RCT)	⊕⊕⊕⊝ Moderate^a^	Foot orthoses likely do not reduce overall pain in the short term compared to heel lifts
Physical function assessed with: OAFQ‐C (Physical) (Higher = better function) Scale from: 0 to 100 follow‐up: 4 weeks	The mean physical function was **64.4** points	MD **1.3 points lower** (7.58 lower to 4.98 higher)	‐	124 (1 RCT)	⊕⊕⊕⊝ Moderate^a^	Foot orthoses likely results in little to no difference in physical function in the short term compared to heel lifts
Participation in sport ‐ not measured	‐	‐	‐	‐	‐	
Withdrawal due to adverse events ‐ not measured	‐	‐	‐	‐	‐	
Adverse events assessed with: Count follow‐up: 12 months	Not pooled as there were no reported adverse events in either group.			101 (1 RCT)	⊕⊕⊕⊝ Moderate^a^	Foot orthoses likely do not increase adverse events compared to heel lifts.
*The risk in the intervention group (and its 95% confidence interval) is based on the assumed risk in the comparison group and the **relative effect** of the intervention (and its 95% CI). **CI:** confidence interval; **MD:** mean difference; **RR:** risk ratio						
**GRADE Working Group grades of evidence** **High certainty:** we are very confident that the true effect lies close to that of the estimate of the effect. **Moderate certainty:** we are moderately confident in the effect estimate: the true effect is likely to be close to the estimate of the effect, but there is a possibility that it is substantially different. **Low certainty:** our confidence in the effect estimate is limited: the true effect may be substantially different from the estimate of the effect. **Very low certainty:** we have very little confidence in the effect estimate: the true effect is likely to be substantially different from the estimate of effect.						
See interactive version of this table: https://gdt.gradepro.org/presentations/#/isof/isof_question_revman_web_464808840774413518.						

^a^ We downgraded one level for imprecision due to one trial with results with a narrow confidence interval

**Summary of findings 5:** Summary of findings table ‐ Heel cushioning compared to heel braces for children with calcaneal apophysitis

**Heel cushioning compared to heel braces for children with calcaneal apophysitis**						
**Patient or population:** children with calcaneal apophysitis **Setting:** Tertiary care **Intervention:** heel cushioning **Comparison:** heel braces						
Outcomes	Anticipated absolute effects^*^ (95% CI)		Relative effect (95% CI)	№ of participants (studies)	Certainty of the evidence (GRADE)	Comments
	Risk with heel braces	Risk with heel cushioning				
Overall pain ‐ not reported	‐	‐	‐	‐	‐	
Physical function in the short term assessed with: OAFQ‐C (Physical) Scale from: 0 to 100 follow‐up: 4 weeks	The mean physical function in the short term was **78** point	MD **2 point lower** (14.17 lower to 10.17 higher)	‐	43 (1 RCT)	⊕⊝⊝⊝ Very low^a,^^b^	The evidence is very uncertain about the effect of heel cushioning on physical function in short term versus a heel strap.
Participation in sport ‐ not measured	‐	‐	‐	‐	‐	
Withdrawal due to adverse events ‐ not measured	‐	‐	‐	‐	‐	
Adverse events	45 per 1000	**45 per 1000** (3 to 665)	**RR 1.00** (0.07 to 14.64)	43 (1 RCT)	⊕⊝⊝⊝ Very low^a,^^b^	The evidence is very uncertain about adverse events of heel cushioning compared to bracing.
*The risk in the intervention group (and its 95% confidence interval) is based on the assumed risk in the comparison group and the **relative effect** of the intervention (and its 95% CI). **CI:** confidence interval; **MD:** mean difference; **RR:** risk ratio						
**GRADE Working Group grades of evidence** **High certainty:** we are very confident that the true effect lies close to that of the estimate of the effect. **Moderate certainty:** we are moderately confident in the effect estimate: the true effect is likely to be close to the estimate of the effect, but there is a possibility that it is substantially different. **Low certainty:** our confidence in the effect estimate is limited: the true effect may be substantially different from the estimate of the effect. **Very low certainty:** we have very little confidence in the effect estimate: the true effect is likely to be substantially different from the estimate of effect.						
See interactive version of this table: https://gdt.gradepro.org/presentations/#/isof/isof_question_revman_web_465419371536866293.						

^a^ We downgraded twice for risk of bias as single study had a high risk of bias ^b^ We downgraded twice for imprecision due to very small participant numbers

## Background

Lower limb apophyseal injuries are common conditions in children and adolescents [1], the two most common being traction apophysitis of the tibial tubercle (eponymous name: Osgood‐Schlatter's disease [2, 3]) and calcaneal apophysitis (eponymous name: Sever's disease [4]). There are lesser known apophyseal injuries that also appear in contemporary medical literature, such as patella‐patellar tendon junction (eponymous name: Sinding Larsen Johansson syndrome [5]), iliac apophysitis (i.e. anterior inferior iliac spine, anterior superior iliac spine, or iliac crest) [6], and base of the fifth metatarsal apophysitis (eponymous name: Iselin's disease [7]). Other injuries that are rarely described include ischial tuberosity or pubic apophysitis [8, 9]. Historical eponymous names for these conditions persist in contemporary literature despite the preferred naming convention being the anatomically correct injury location. These conditions are non‐infectious, rarely result from a single‐point trauma, and are a separate entity to avulsion fractures of the apophysis. It is unknown why these injuries occur in some children more than others.

Apophyseal injuries are often grouped with other types of osteochondral injuries, despite apophyseal injuries being distinctly different. Osteochondral lesions or avascular necroses are typical osteochondral injuries, one of the better known ones being avascular necrosis of the femur (Perthes disease). Apophyseal injuries do not result in bone destruction [10], but commonly result in increased bone oedema [11, 12]. Apophyseal injuries do not follow the trajectory of other osteochondral injuries, such as bone remodelling, change in joint range of motion, or painful position change [13]. Instead, children with apophyseal injuries describe pain localised to the apophysis, and it is the localised pain during activity that makes the diagnosis relatively straightforward.

Apophyseal injuries appear to occur relatively common according to sports medicine literature, and children attend general and sports medicinal health professional practices. However, prevalence and incidence data sourced from general population studies are only available for traction apophysitis of the tibial tubercle and calcaneal apophysitis [1]. The point‐prevalence of traction apophysitis of the tibial tubercle amongst the general population of 12‐ to 15‐year‐old children (n = 956) was estimated to be 9.83% (95% confidence interval (CI) 8.02% to 11.90%) in a 2011 study [14]. The lifetime incidence of traction apophysitis of the tibial tubercle amongst a general population sample (n = 389) was 12.85% (95% CI 9.69% to 16.95%) was reported in 1985 [15]. Calcaneal apophysitis has an incidence of 0.0037 per person‐year in the general population of children aged between six and 17 years (n = 16,530) [16], but there are no data available to calculate its point‐prevalence or lifetime incidence [1]. There are no general population estimates of prevalence or incidence rates of iliac apophysitis, osteochondrosis at the patella‐patellar tendon junction, or base of the fifth metatarsal apophysitis [1].

### Description of the condition

Apophyseal injuries appear in secondary growth centres of bones. These growth centres appear at different ages depending on whether the children are male or female, and their puberty status. For example, in girls, the calcaneal apophysis may appear between six and 12 years of age, but in boys, it appears between eight and 14 years of age [10, 17]. There are similar age and sex differences for the apophysis of the tibial tuberosity. In girls, it is commonly present between the ages of eight and 12 years, but in boys, between 12 and 15 years [18]. Rapid growth and weight changes during these ages are considered the primary contributing factor as to why these injuries also appear during this period of growth.

An apophyseal injury is diagnosed based on its location on the limb and skeletal maturity. This means, as the apophysis appears on the limb, some children may then have pain in that area when being physically active. Children who routinely play sport are also at a higher risk of having pain in these areas during the time when secondary apophysis is present [1]. Common symptoms are localised pain in the apophyseal region that is made worse by activity and eases with ceasing the activity or resting. Clinicians differentially rule out fractures, ligament injuries, bursitis, muscle strain or sprain, inflammatory conditions, or other bone conditions. This means apophyseal injuries are commonly diagnosed through a series of questions and a physical examination. Medical imaging is rarely needed [10, 19].

The precise reason for apophyseal injuries remains unknown. The healthy staged formation of an apophysis in the heel or knee can, at times, display similar features on magnetic resonance imaging (MRI) to those of children describing pain in the region and diagnosed clinically with an apophyseal injury [20, 21]. This presence (or absence) of particular imaging features has led clinicians to wonder about inflammation being a marker (or not) of an apophysitis diagnosis. Many authors propose that apophyseal injuries are as a result of traction from tight tendons/muscles resulting from rapid growth during puberty. For example, calcaneal apophysitis has been hypothesised to be caused by rapid tibial shaft growth exceeding the rate of lengthening in the gastrocnemius/soleus complex [22]. However, research indicates this is unlikely [23]. It has also been suggested that compression vertical velocity may be a contributing factor for calcaneal apophysitis, though observational research does not support this theory [24]. Apophyseal injuries present at the knee or hip are thought to be related to repetitive muscle contraction and overuse at each location [25], and may often coexist with insertional tendinopathies [9].

Many apophyseal injuries are self‐limiting. However, the pain these injuries cause during physical activity has been shown to impact children's quality of life [26, 27] and their participation in their usual physical activities [27]. There is increasing evidence that some apophyseal injuries, such as traction apophysitis of the tibial tubercle, can also result in chronic pain and ongoing physical activity limitation into adulthood [28, 29]. This variability in impact complicates clinical decision‐making about whether intervention is warranted, and the type of intervention, if so.

### Description of the intervention and how it might work

The most common interventions for apophyseal injuries are non‐surgical and are justified on their proposed pathophysiology and the basis of physical or mechanical load management. Load management is thought to modify the tension or strength of the muscle/tendon attaching around the painful apophysis [30]. These techniques may be through restriction in movement via resting, stopping physical activity entirely, or immobilisation [18]; modified exercise [31] or changing physical activity types [32]; or through pressure redistribution such as footwear with particular features, with or without the use of heel raisers (lifts) [33, 34] or foot orthoses [33, 34, 35]. All these interventions have been studied within different trial designs, finding variable success in pain resolution and improvement in children's quality of life.

Other treatment modalities initiated with the aim of reducing any associated inflammation or short‐term pain include the use of topical agents (ice or topical non‐steroidal anti‐inflammatory drugs (NSAIDs)) [36], oral NSAIDs or analgesics [36, 37], or stretching to reduce a real or perceived temporary muscle length deficit [36, 38, 39]. These treatments have not been tested rigorously in any trial design to understand their success for any outcomes.

Many of these treatments are used for any or all lower limb apophyseal injuries, regardless of their location.

### Why it is important to do this review

Apophyseal injuries are common and are known to impact children's quality of life [26, 31]. These injuries may cause interruptions or cessation of physical activity at a period in a child's or young person's development where it may be difficult to return to sport [40]. They may also impact the training and competition schedule of junior athletes. Often the pain from these conditions continues for up to 12 months or more [29]. For some children, persistent pain and its impact on quality of life may fulfil the ICD‐11 criteria for chronic musculoskeletal pain, depending on whether the pain is considered primary or secondary to the underlying apophysitis [31, 33, 41].

Children with apophyseal injuries and their parents commonly present to primary care, sports medicine clinics, other community health service settings, or a combination of these. Of the wide range of treatment approaches posited in the literature, many are based on theoretical mechanisms of action not supported by observational data or cohort studies, which indicates a high likelihood that ineffective, inefficient, and possibly even harmful treatment approaches may be prescribed in these settings. Moreover, prescribing some of these treatments may come at an excess cost to both the health system and families. This elevates the importance of synthesising the evidence for treatments of these types of injuries in one review.

## Objectives

To assess the benefits and harms of non‐surgical treatment versus placebo, no treatment, or another treatment on overall pain, physical function, or participation in physical activity in children and adolescents with lower limb apophyseal injuries.

## Methods

We conducted this review in accordance with the Methodological Expectations of Cochrane Intervention Reviews (MECIR), and followed PRISMA 2020 guidelines [42], as well as the *Cochrane Handbook for Systematic Reviews of Interventions* [43].

### Differences between protocol and review

1. We identified and included studies where one intervention was tested against another intervention. These individual interventions were included in our original protocol [44] and are commonly used in practice.
2. Following expert advice from Cochrane, we prioritised and reduced the number of critical outcomes to overall pain, physical function, participation in sport, and physical activity, withdrawals due to adverse events, and adverse events. We reported the main findings from trials only where critical outcomes were reported in the short term (< 3 months) and withdrawal due to adverse events and adverse events were reported at the trial's conclusion. We moved participant‐reported treatment sucess to Important outcomes.
3. Lastly, we had planned to use the Cochrane tool RoB 1 for the risk of bias assessment, but we used RoB 2, which is the current Cochrane‐recommended version.

### Criteria for considering studies for this review

#### Types of studies

We included randomised controlled trials (RCTs) reported as full text, abstract only, or unpublished data. We included cross‐over studies but not cluster‐randomised designs. There were no language restrictions.

#### Types of participants

We included studies that recruited children and young people aged between seven and 19 years with a diagnosis of the following apophyseal injuries: traction apophysitis of the tibial tubercle (Osgood‐Schlatter's disease), calcaneal apophysitis (Sever's disease), Iliac apophysitis, osteochondrosis at the patella‐patellar tendon junction (Sinding Larson Johansson syndrome), and base of the fifth metatarsal traction apophysitis (Iselin's disease).

We excluded trials of children and young people who had an underlying inflammatory disorder.

#### Types of interventions

We included trials that evaluated the effect of the following non‐surgical treatments. We grouped these according to the following list:

1. Reduction of load through abstinence from organised sporting activity or modification of sporting activity with specific dosage information (i.e. time, sporting activity).
2. Complete immobilisation (i.e. time, type).
3. Stretching exercises (guided either by a health professional within the clinical setting or implemented within the home environment) with specific dosage information (i.e. frequency, timing, type).
4. Exercise (balance, alignment/postural, strength, stability specified) with specific dosage information (i.e. frequency, timing, type).
5. Psychological focused interventions such as pain science education.
6. Pharmaceutical interventions such as prescribed or non‐prescription medicines, including topical, oral, or injected medications.
7. Foot orthoses, bracing, taping with sports tape, straps, all with specific information about wear time (e.g. hours per day), materials (e.g. stretch, non‐stretch), and force distribution properties (e.g. density).
8. Footwear or heel cushioning with specific information (e.g. wear time, heel pitch, material).
9. Heel lifts with specific information (e.g. wear time, material, height).
10. Manual therapy such as dry needling or massage with specific dosage information (i.e. body location, frequency, timing, type).
11. Extracorporeal shock wave therapy with specific dosage information (e.g. body location, frequency).

We also included trials if they used a combination of treatments.

Our comparators were as follows.

1. Placebo
2. No treatment including:
  - wait list (no other description provided by the trial authors);
  - control group (described specifically as no intervention; or no other description provided by the trial authors);
  - usual/normal care (where it was stated that participants could receive usual care, but this was not controlled by trial);
  - non‐surgical treatment and comparison groups offered, or received, the same co‐interventions, allowing the effect of the non‐surgical treatment to be isolated.
3. Another intervention

### Outcome measures

There is limited investigation into what parents, children, and young people consider to be critical outcomes with these conditions. Instead, many studies report subjective measures of intermittent pain and intermittent rest patterns. We focused our critical outcome domains on the International Classification of Function, Disability and Health – Child and Youth Version [45]: body function and structure impairment, activity, participation, quality of life, distress, and well‐being. We did not exclude studies on the basis of outcome reporting.

#### Timing of outcome measures

For this review, we prioritised reporting outcome data from the first assessment after treatment in the short term (< 3 months) for outcomes other than withdrawals due to adverse events and adverse events. We reported withdrawals due to adverse events and adverse events at the trial's conclusion.

Where available, we also assessed all overall pain, physical function, participation in sports or physical activity, treatment success, pain during a predefined activity, and quality of life, in the medium term (three to nine months after the start of the intervention) and long term (greater than nine months after the start of the intervention).

#### Critical outcomes

1. Self‐reported overall pain measured subjectively on a visual analogue scale (VAS) [46] or numerical rating scale (NRS) [47]
2. Self‐reported physical function measured subjectively by Lower Extremity Functional Scale (LEFS) [48], Knee Injury and Osteoarthritis Outcome Score Child Version — Function in Sport/Play subscale [49], Oxford Ankle Foot Questionnaire for Children (OxAFQ‐C) [50], Foot and Ankle Ability Measures [51], or other physical function scale appropriate to the age and body location
3. Self‐reported participation in sports or physical activity measured subjectively through trial participant diary or other self‐reporting tool
4. Withdrawals due to adverse events
5. Adverse events reported by a count

#### Important outcomes

1. Participant‐reported treatment success measured subjectively by a global rating of treatment such as the Patient Global Impression of Change (PGIC) scale [52], or overall treatment success as defined in the trials (e.g. proportion without pain; proportion with 25% pain; disability reduction)
2. Self‐reported pain experienced during a predefined activity measured subjectively on a VAS, NRS, or other scale
3. Active range of motion of the relevant lower limb joint measured objectively by a trained assessor using goniometry (degree of change from baseline)
4. Self‐reported, or parent proxy‐reported, quality of life measured subjectively by different self‐reported measures, such as the EuroQol 5‐Dimension 5‐Level (EQ‐5D‐5L) [53]

We used a priori decision rules regarding which data to extract in the event of multiple‐outcome reporting. Where study authors reported outcomes for more than one pain scale, we planned to extract data from the VAS before any other pain scale or subscale data. Where study authors reported outcomes for more than one function scale, we planned to extract data from the LEFS [48] before any other function scale or subscale. Where study authors reported outcomes for more than one quality of life scale, we planned to extract data from the EuroQol 5‐Dimensions 3‐Levels – Youth version (EQ‐5D‐3L‐Y) scale [54] before any other quality of life scale or subscale. We extracted change‐from‐baseline data.

### Search methods for identification of studies

#### Electronic searches

We searched the following electronic databases on 5 January 2024 and 4 January 2025.

1. Cochrane Central Register of Controlled Trials (CENTRAL; 2025, Issue 1) via Ovid
2. MEDLINE via Ovid (1966 to 4 January 2025)
3. Embase via Ovid (1980 to January 2025)
4. CINAHL Plus (1937 to January 2025)

We also searched ClinicalTrials.gov (clinicaltrials.gov) and the World Health Organization trials portal (www.who.int/ictrp/en/) for ongoing and recently completed trials. We did not impose any restrictions on the language of publication. We used the recommended Cochrane Highly Sensitive Search Strategy to identify randomised trials in Ovid: sensitivity‐maximising version (2008 revision) filter [55]. All search strategies are listed in Supplementary material 1.

#### Searching other resources

We checked the reference lists of all included studies and of relevant review articles for additional references. We contacted study authors for participant‐level data where appropriate. We also searched for errata or retractions for included studies published in full text on PubMed (https://www.ncbi.nlm.nih.gov/pubmed).

### Data collection and analysis

We used Covidence software to facilitate the selection of studies.

#### Selection of studies

Two review authors (KP and AC) independently screened the titles and abstracts identified through our search. We classified the studies as potentially eligible/unclear or not eligible. The same two review authors (KP and AC) retrieved the full‐text of potentially eligible/unclear publications and independently screened them against the inclusion criteria. We recorded the reasons for exclusion of the ineligible studies in a 'Characteristics of excluded studies' table. We resolved any disagreements through discussion.

We identified and removed any duplicates and collated multiple reports of the same study so that each study, rather than each report, was the unit of interest in the review. We recorded the selection process in sufficient detail to complete a PRISMA flow diagram ([56]; prisma‐statement.org/PRISMAStatement/Default.aspx) and a 'Characteristics of included studies' table.

#### Data extraction and management

We used a data collection form for study characteristics and outcome data in Covidence software. Two review authors (AC and KP) independently extracted and checked the following characteristics from included studies.

1. Methods: study design, total duration of study, study location and setting, withdrawals, and date of study
2. Participants: number, mean age, age range, sex, type of apophyseal injury, duration of apophyseal injury, baseline primary or secondary outcome data, inclusion criteria and exclusion criteria, country of residence, physical activity participation
3. Interventions: intervention (e.g. group, dose as described in the publication), comparison
4. Outcomes: critical and important outcomes specified and collected, and time points reported
5. Characteristics of the design of the trial as outlined in the Risk of bias assessment in included studies section.
6. Notes: funding for trial, and notable declarations of interest of trial authors

We extracted the total number of participants and number of participants per treatment group for dichotomous outcomes, and means and standard deviations and number of participants per treatment group for continuous outcomes. We noted in the 'Characteristics of included studies' table if outcome data were not reported in a usable way and when data were transformed or estimated from a graph. We resolved disagreements by consensus or by involving a third review author (CW or KT). One review author (CW) transferred data into Review Manager (RevMan) [57]. Another review author (KP) double‐checked that data were entered correctly by comparing the data presented in the systematic review with the study reports.

#### Risk of bias assessment in included studies

Two review authors (KP and AC) independently assessed risk of bias for each study using the Cochrane risk of bias tool RoB 2 and the criteria outlined in the *Cochrane Handbook for Systematic Reviews of Interventions* [58]. We resolved any disagreements by discussion or by involving another review author (CW or KT). We assessed the risk of bias according to the following domains.

1. Bias arising from the randomisation process
2. Bias due to deviations from the intended interventions
3. Bias due to missing outcome data
4. Bias in measurement of the outcome
5. Bias in the selection of the reported results

We assessed each potential source of bias as high, low, or 'some concerns', and provided information from the study report together with a justification for our judgement in the risk of bias table. We summarised the risk of bias judgements across different studies for each of the domains listed. We considered blinding separately for different key outcomes where necessary (e.g. for self‐reported and assessor‐reported outcomes). In addition, we considered the impact of missing data for each key outcome [59].

Where information on the risk of bias related to unpublished data or correspondence with a trial author, we noted this in the risk of bias table. When considering treatment effects, we considered the risk of bias for the studies that contributed to that outcome.

We used figures generated by the RoB 2 tool to provide a summary assessment of the risk of bias.

#### Measures of treatment effect

We analysed dichotomous data as risk ratios (RR) and 95% CIs. We analysed continuous data as mean difference (MD) if studies used the same scales, and standardised mean difference (SMD) if they used different scales, with 95% CIs. We entered data presented as a scale with a consistent direction of effect across studies.

For dichotomous outcomes, we calculated the absolute percentage change from the difference in the risks between the intervention and control group using GRADEpro GDT [60], and expressed it as a percentage.

In the 'Effects of interventions' section and the 'What happens' column of the summary of findings tables, we provided the absolute percentage change and the SMD or MD (change from baseline), where possible. There are no established minimally important clinical differences in the outcome measures associated with apophyseal injuries in childhood, so we interpreted the results cautiously.

#### Unit of analysis issues

Where a trial reported multiple trial arms, we included the relevant arms. If two comparisons (e.g. orthoses versus watch and wait and heel lifts versus watch and wait) were combined in the same meta‐analysis, we halved the control group to avoid double‐counting. If items were reported narratively, we reported each arm, as per the guidance in Chapter 23 in the *Cochrane Handbook*. Where a trial reported a cross‐over design, we used the outcomes for the first time point. This is because apophyseal injuries can resolve with time and the effects of any wash‐out period are unknown for any interventions.

#### Dealing with missing data

We contacted investigators or study sponsors in order to verify key study characteristics and obtain missing numerical outcome data when a study was identified as abstract only or when data was not available for all participants. Where this was not possible, and we thought the missing data may create serious bias, we described the study results narratively.

For continuous outcomes (e.g. mean change in pain score), we calculated the MD or SMD based on the number of participants analysed at that time point.

We computed missing standard deviations from other statistics such as standard errors, CIs, or P values, according to the methods recommended in the *Cochrane Handbook for Systematic Reviews of Interventions* [61].

#### Reporting bias assessment

We were not able to pool more than 10 trials as we had planned to do in our protocol.

To assess outcome reporting bias, we checked trial protocols against published reports. For studies published after 1 July 2005, we screened the Clinical Trial Register at the International Clinical Trials Registry Platform of the World Health Organization (www.who.int/ictrp/en/) for the a priori trial protocol. We evaluated whether selective reporting of outcomes was present.

#### Synthesis methods

We created summary of findings tables for all comparisons and all time points. In our main summary of findings tables, we presented results for all trials reporting critical outcomes at the primary time point (i.e. short term, less than three months).

1. Pharmaceutical intervention compared to placebo (primary comparison)
2. Pharmaceutical interventions compared to usual care
3. Taping compared to a placebo
4. Foot orthoses compared to heel lifts
5. Heel cushioning compared to heel straps

We also synthesised important outcomes for these comparisons if reported.

Where trials only reported important outcomes, and any outcomes at other time points, we reported these in the summary findings tables in supplementary materials.

We used the random‐effects meta‐analysis model to pool study data. We included all trials in the primary analysis. We used the random‐effects model as we anticipated diversity in outcome measurement types. The Restricted Maximum Likelihood (REML) estimator was used to estimate between‐trial variance. The Hartung‐Knapp‐Sidik‐Jonkman method was used to calculate a confidence interval for the meta‐analysis effect estimate when there were at least two studies, and the estimate of heterogeneity was greater than zero. There were no other scenarios requiring a different method.

We planned to stratify by condition for subgroup analysis, but this was not possible.

We undertook meta‐analyses only where this was meaningful (i.e. where studies used the same treatment strategy). We made only the following comparisons.

1. Intervention versus placebo (in the order these treatment groups are listed in Types of interventions)
2. Intervention versus no intervention (wait‐list control)
3. Intervention versus intervention (where the intervention had been used within a trial and compared to another intervention)

We assessed clinical and methodological diversity in terms of participants, interventions, outcomes, and study characteristics for the included studies to determine whether a meta‐analysis was appropriate. We conducted this assessment by inspecting these data in the data extraction tables. We assessed statistical heterogeneity by visual inspection of the forest plot to assess for obvious differences in results between the studies, and using the I² and Chi² statistical tests. We interpreted the I² value as recommended in the *Cochrane Handbook for Systematic Reviews of Interventions* [61]: an I² value of 0% to 40% might 'not be important'; 30% to 60% may represent 'moderate' heterogeneity; 50% to 90% may represent 'substantial' heterogeneity; and 75% to 100% represents 'considerable' heterogeneity. As noted in the *Cochrane Handbook for Systematic Reviews of Interventions* [61], we kept in mind that the importance of the I^2^ statistic depends on 1) the magnitude and direction of effects, and 2) the strength of evidence for heterogeneity. We interpreted the Chi² test with a P value of 0.10 or less to indicate evidence of statistical heterogeneity.

If we identified substantial heterogeneity, we reported it and investigated possible causes by following the recommendations in Section 10.10 of the *Cochrane Handbook for Systematic Reviews of Interventions* [61].

#### Investigation of heterogeneity and subgroup analysis

Due to a lack of trials reporting relevant data, we were unable to conduct our planned subgroup analyses by sex, sports participation, body mass index centile, or condition.

##### Equity‐related assessment

We did not investigate equity‐related characteristics in this review due to the lack of diversity in the data. The study data were primarily collected in secondary or tertiary care (e.g. sports medicine clinics), which may have inadvertently impacted which children could access care and participate in clinical trials.

#### Sensitivity analysis

Due to limited data, we did not carry out any sensitivity analyses.

#### Certainty of the evidence assessment

We created a main summary of findings table using the following critical outcomes.

1. Self‐reported overall pain
2. Self‐reported physical function
3. Self‐reported participation in sport
4. Withdrawals due to adverse events
5. Serious adverse events

Two review authors (AC and KP) independently assessed the certainty of the body of evidence (the studies that contributed data to the meta‐analyses for the prespecified outcomes) using the five GRADE considerations (study limitations, consistency of effect, imprecision, indirectness, and publication bias), and we reported this as high, moderate, low, or very low. We used methods and recommendations described in Chapter 14 of the *Cochrane Handbook for Systematic Reviews of Interventions* [62]. We used GRADEpro GDT software to prepare the summary of findings tables [60]. We used version 3 of the GRADEpro view to display our summary of findings tables. We justified all decisions to downgrade the certainty of evidence for each outcome using footnotes, and we made comments to aid the reader's understanding of the review where necessary.

1. High certainty: we are very confident that the true effect lies close to that of the estimate of the effect.
2. Moderate certainty: we are moderately confident in the effect estimate: the true effect is likely to be close to the estimate of the effect, but there is a possibility that it is substantially different.
3. Low certainty: our confidence in the effect estimate is limited: the true effect may be substantially different from the estimate of the effect.
4. Very low certainty: we have very little confidence in the effect estimate: the true effect is likely to be substantially different from the estimate of effect.

### Consumer involvement

We did not have consumer involvement in this review. However, management of apophyseal injuries was within the top 15 condition groupings prioritised through consultation with health professionals and families to support development of guidance for managing children’s musculoskeletal disorders resulting in chronic pain [63].

## Results

### Description of studies

#### Results of the search

The search was conducted up to 4 January 2025. We identified 1670 studies. After automation tools within Covidence removed 314 ineligible studies and 212 duplicates, there were 1142 articles to be screened. Of those 1142, we assessed 32 in full text. We excluded 12 articles due to ineligible study design, four because participant populations were ineligible, and three as we were unable to source data or the trial had been abandoned. We identified three recently completed unpublished trials. Table provides detailed characteristics of studies that did not have results available at the time we submitted this review for editorial processing (Carl 2025 [64]; Faude 2020 [65]; Krommes 2025 [66]). We included 10 unique studies in the review. See Figure for a flow diagram of the study selection process.

**1:** Table 1. Completed studies awaiting publication of results

Trial registration number and/or study reference	Principal investigator (or sponsor)/country	Apophysitis type	Intervention arms	Main selection criteria	Outcome/s	Registration date	Recruitment commenced	Trial status to 1 May 2025	Planned sample size	Actual sample size	Planned follow‐up
Carl 2025	Rebecca Carl, USA	Traction apophysitis of the tibial tubercle (Osgood‐Schlatter's disease) and calcaneal apophysitis (Sever's disease)	No intervention versus static stretch (group stretching exercises) versus active elongation (group: exercise)	Diagnoses of the conditions and regular access to the internet	Overall pain, Self‐reported participation in sports	29/03/2013	10/2012	Completed 10/2023	Unknown	129	8 weeks
Faude 2020	Oliver Faude, Switzerland	Traction apophysitis of the tibial tubercle (Osgood‐Schlatter's disease)	Usual care versus physiotherapy programme (group: exercise)	Diagnosis of the condition, ability to follow instructions and participate in two exercise sessions per week for 8 weeks	Self‐reported physical function	19/01/2021	11/11/2020	Completed 07/07/2022	Unknown	36	8 weeks
Krommes 2025	Kasper Krommes, Denmark	Traction apophysitis of the tibial tubercle (Osgood‐Schlatter's disease)	Usual care versus exercise and education (group: exercise)	Pain at site, aggravated by specific movements and reduced sport participation	Self‐reported physical function	30/11/2021	01/01/2022	Completed 08/03/2024	130	130	5 months

**1:**
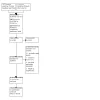
Flow diagram

#### Included studies

Ten studies met our inclusion criteria (Alfaro‐Santafa 2021 [67, 68]; James 2016 [69]; Kuyucu 2017 [70]; Nakase 2020 [71]; Perhamre 2011a [72]; Perhamre 2012 [73]; Reesman 2024 [74]; Sweeney 2023 [75, 76]; Topol 2011 [77]; Wiegerinck 2016 [78]). A full description of these studies can be found in Supplementary material 2, and a summary of interventions and participants is provided in Table. We contacted one study author to retrieve raw participant data (James 2016). However, these data had already been destroyed as per the trial protocol.

**2:** Table 2. Summary characteristics of included studies aligning to the intervention categories

Study name, year, country of conduct	Study design	Apophysitis region	Population (sample size: intervention/control)	Intervention/control summary	Outcome domains with available data	Specific outcome measure	Time point of measurement	Method of synthesis
Intervention group 6: pharmaceutical interventions Placebo								
Nakase 2020, Japan	RCT (parallel)	Tibial tubercle	‐ Failed conservative intervention > 1 month (25 knees/24 knees)	‐ 1% lidocaine (1 mL) with 20% dextrose (1 mL; dextrose group) ‐ 1% lidocaine (1 mL) with saline (1 mL; saline group	**Critical:** 4. Withdrawal due to adverse events 5. Total adverse events **Important** 2. Self‐reported pain experienced during a predefined activity	**Critical** 4. Count 5. Count **Important** 2. VISA score	4 weeks (short) 3 months (medium)	**Important:** meta‐analysis and summary
Intervention group 7: foot orthoses, bracing, taping with sports tape, straps Placebo								
Kuyucu 2017, Turkey	RCT (parallel)	Calcaneal	Junior footballers diagnosed with calcaneal apophysitis (total n = 22/unknown group distribution)	‐ Kinesio tape ‐ Sham tape	**Critical:** 1. Self‐reported overall pain 2. Self‐reported physical function	**Critical:** 1. VAS 2. American Orthopedic Foot‐Ankle Society score	1 week (short) 3 months (medium)	**Critical:** summary
Intervention group 7: foot orthoses, bracing, taping with sports tape, straps No treatment								
Perhamre 2012, Sweden	RCT (parallel)	Calcaneal	Children 9 to 15 years with confirmed calcaneal apophysitis present for > 2 weeks, with pain during a ball sport being 4 or greater on the BorgCR‐10 in the last week. High level of sport engagement (15/15)	‐ Custom‐made foot orthoses ‐ No treatment	**Important:** 2. Self‐reported pain experienced during a predefined activity	**Important:** 2. Borg’s CR‐10	4 weeks (short)	**Important:** summary
Intervention group 6: pharmaceutical interventions Placebo Usual care								
Topol 2011, Argentina	RCT (parallel)	Tibial tubercle	‐ Girls aged 9 to 15 years or boys aged 10 to 17 years with anterior knee pain and involved in jumping or kicking sports (17/18/19)	‐ 1% lidocaine with 12.5% dextrose (2 mL) ‐ 1% lidocaine with saline (2 mL; saline group ‐ Usual care	**Important:** 2. Self‐reported pain experienced during a predefined activity	**Important:** 2. Nirschl Pain Phase Scale	3 months (medium)	**Important:** meta‐analysis
Reesman 2024, USA	RCT (parallel)	Tibial tubercle	‐ Children 7 to 14 years, able to identify pain/burns during treatment, confirmed apophysitis and knee pain impacting on activities of daily living (16/15/14)	‐ Dexamethasone (DPS 0.75 mg) delivered through iontophoresis ‐ 0.9% sodium chloride delivered through iontophoresis ‐ Usual care	**Critical:** 1. Self‐reported overall pain 2. Self‐reported physical function 3. Self‐reported participation in sport 5. Total adverse events	**Critical:** 1. Wong‐Baker FACES Pain Rating Scale 2. Lower Extremity Function Scale 3. Return to sport checklist 5. Count	4 weeks (short)	**Critical:** summary **Important:** meta‐analysis
Intervention group 4: exercise Intervention group 9: heel lifts No treatment								
Wiegerinck 2016, Netherlands	RCT (parallel)	Calcaneal	Children aged 8 to 15 years with diagnosed calcaneal apophysitis (33/33/32)	‐ Exercise programme from physical therapist ‐ ViscoHeel ‐ heel lift ‐ No treatment	**Critical:** 2. Self‐reported physical function 5. Total adverse events **Important:** 1. Participant‐reported treatment success 2. Self‐reported pain experienced during a predefined activity	**Critical:** 2. OxAFQ‐C 5. Count **Important:** 1. VAS of satisfaction 3. Faces Pain Scale‐Revised during Algometer	6 weeks (Short) 3 months (medium)	**Critical:** summary **Importan:** summary
Intervention group 7: foot orthoses, bracing, taping with sports tape, straps Intervention group 8: footwear or heel cushioning								
Sweeney 2023, USA	RCT (parallel)	Calcaneal	Children 7 to 14 who participate in barefoot sport (16/16)	‐ X‐brace ‐ Cheetah heel cup	**Critical:** 2. Self‐reported physical function 5. Total adverse events **Important:** 2. Self‐reported pain experienced during a predefined activity	**Critical:** 2. OxAFQ‐C 5. Count **Important:** 2. VAS	4 weeks (short) 3 months (medium)	**Critical:** summary **Important:** summary
Intervention group 7: foot orthoses, bracing, taping with sports tape, straps with or without standardising the footwear Intervention group 9: heel lifts with or without standardising the footwear								
Alfaro‐Santafa 2021, Spain	RCT (parallel)	Calcaneal	Children 9 to 12 years (104/104)	‐ Custom made foot orthoses ‐ heel lift	**Critical** 1. Self‐reported overall pain **Important:** 2. Self‐reported pain experienced during a predefined activity	**Critical** 1. VAS **Important:** 2. Algometry	3 months (medium)	**Critical:** summary **Important:** summary
James 2016, Australia	RCT (factorial)	Calcaneal	Children aged 8 to 14 years (31/31/31/31)	‐ Prefabricated foot orthoses with usual or standardlised footwear ‐ Heel lift with usual or standardised footwear	**Critical:** 1. Self‐reported overall pain 2. Self‐reported physical function 5. Total adverse events **Important** 3. Active range of motion of the relevant lower limb joint	**Critical:** 1. Faces Pain Scale 2. Oxford Ankle Foot questionnaire 5. Count **Important** 3. Weight‐bearing lunge test	4 weeks (short for all outcomes) 6 months (medium for critical 2 and 5 only) 12 months (long for critical 2 and 5)	**Critical:** summary **Importan:** summary
Perhamre 2011a, Sweden	RCT (cross‐over)	Calcaneal	Males, aged 9 to 15 years, history of calcaneal apophysitis for > 2 weeks but < 26 weeks. High level of sport engagement	‐ Customised thermoplastic heel cup (foot orthoses) ‐ heel lift	**Important:** 2. Self‐reported pain experienced during a predefined activity	**Important:** 2. Borg’s CR‐10	4 weeks (short)	**Important:** summary

Borg’s CR‐10: Borg's Category Ratio‐10; OxAFQ‐C: Oxford Ankle Foot Questionnaire for Children; VAS: visual analogue scale; VISA: Victorian Institute of Sport Assessment

There were five studies in Europe, two of which were by the same author group (Alfaro‐Santafa 2021; Kuyucu 2017; Perhamre 2011a; Perhamre 2012; Wiegerinck 2016), two studies in North America (Reesman 2024; Sweeney 2023), one in South America (Topol 2011), and one in Oceania (James 2016). Nine studies were RCTs of parallel design (Alfaro‐Santafa 2021; James 2016; Kuyucu 2017; Nakase 2020; Perhamre 2012; Reesman 2024; Sweeney 2023; Topol 2011; Wiegerinck 2016); the other study used a cross‐over design (Perhamre 2011a). One study was discontinued early due to the COVID‐19 pandemic and recruitment issues, with limited data entered into the trial registry; we included these data (Reesman 2024).

Table outlines baseline data from the 10 studies. Seven focused on calcaneal apophysitis (Alfaro‐Santafa 2021; James 2016; Kuyucu 2017; Perhamre 2011a; Perhamre 2012; Sweeney 2023; Wiegerinck 2016) and three focused on traction apophysitis of the tibial tubercle (Nakase 2020; Reesman 2024; Topol 2011). Two studies specifically recruited based on sport type: football (Kuyucu 2017) or gymnastics (Sweeney 2023), while a further four focused on children who regularly played sport for at least six hours a week (Nakase 2020; Perhamre 2011a; Perhamre 2012; Wiegerinck 2016); the remaining studies were not focused on particular sport or amount of time spent in sport.

**3:** Table 3. Summary of baseline data of each trial

Study name and year	Apophysitis region	Total sample size at baseline	Duration of symptoms	Sport participation level or activities	Sex ‐ male (%)	Age	Intervention groups and participant n at baseline	Baseline (critical outcomes) mean (SD)	Baseline (important outcomes) mean (SD) or median (IQR)
Intervention group 6: pharmaceutical interventions Placebo									
Nakase 2020	Tibial tubercle	49 knees in 38 participants	6.7 (6.2) months	All played sport more than 3 times per week	37 (97%)	12.4 (0.9)	1% lidocaine 1 ml in 20% dextrose n = 2 5 knees	N/A	2. Self‐reported pain experienced during a predefined activity ‐ VISA score Mean (SD) = 58.7 (18.3)
			7.1 (8.2 (months)			12.4 (1.2)	Placebo (1% lidocaine 1 ml in saline ) n = 24 knees	N/A	2. Self‐reported pain experienced during a predefined activity ‐ VISA score Mean (SD) = 63.4 (16.4)
Intervention group 7: foot orthoses, bracing, taping with sports tape, straps Placebo									
Kuyucu 2017	Calcaneus	22	NR	Regularly played football	22 (100 %)	Overall mean 13.18 years	NR	1. Self‐reported overall pain Wong‐Baker FACES Mean (SD) = 7.0 (0.9) 2. Self‐reported physical function American Orthopedic Foot‐Ankle Society score Mean (SD) = 62.4 (12.8)	NA
								1. Self‐reported overall pain (Wong‐Baker FACES) Mean (SD) = 6.8 (1.2) 2. Self‐reported physical function American Orthopedic Foot‐Ankle Society score Mean (SD) = 70.5 (6.0)	
Intervention group 7: foot orthoses, bracing, taping with sports tape, straps No treatment									
Perhamre 2012	Calcaneus	30	NR	All regularly participated in sport at Engstom's five level activity	NR	NR	Foot orthoses n = 15	NA	2. Self‐reported pain experienced during a predefined activity ‐ Borg's CR‐10 Median = 2.0
							No treatment n = 15		2. Self‐reported pain experienced during a predefined activity ‐ Borg's CR‐10 Median = 7.0
Intervention group 6: pharmaceutical interventions Placebo Usual care									
Topol 2011	Tibial tubercle	65 knees in 54 participants	NR	NR	51 (94%)	Overall mean 13.3)	Lidocaine 1% and 12.5% dextrose n = 17	NA	2. Self‐reported overall pain during activity NPPS Mean (SD) = 4.6 (1.0)
							Placebo (lidocaine 1%) n = 18		2. Self‐reported overall pain during activity NPPS Mean (SD) = 4.2 (1.0)
							Usual care n = 19		2 Self‐reported overall pain during activity NPPS Mean (SD) = 4.3 (1.0)
Reesman 2024	Tibial tubercle	45	NR	NR	7 (44%)	NR	Iontophoresis with dexamethasone n = 16	NR	NR
					11 (73%)		Placebo (Iontophoresis with saline) n = 15		
					8 (57%)		Usual care n = 14		
Intervention group 4: exercise Intervention group 9: heel lifts No treatment									
Wiegerinck 2016	Calcaneus	98	< 4 weeks	Hours in sport participation per week (mean (SD): 6.3 (3.3)	27 (82%)	10.6 (1.4)	Exercise n = 33	2. Self‐reported physical function ‐ Oxford Ankle foot questionnaire child overall score Mean (SD) = 43.0 (7.7)	2. Self‐reported pain experienced during a predefined activity FPS‐R mean (SD) where the activity was pain on direct pressure of the growth plate Mean (SD) = 6.3 (1.7)
				6.7 (3.7)	22 (67%)	10.3 (1.4)	Heel lifts n = 33	2. Self‐reported physical function ‐ Oxford Ankle foot questionnaire child overall score Mean (SD) = 39.2 (8.5)	2. Self‐reported pain experienced during a predefined activity FPS‐R mean (SD) where the activity was pain on direct pressure of the growth plate Mean (SD) = 6.2 (2.0)
				6.6 (2.7)	27 (77%)	10.9 (1.9)	No treatment n = 32	2. Self‐reported physical function ‐ Oxford Ankle foot questionnaire child overall score Mean (SD) = 42.3 (8.4)	2. Self‐reported pain experienced during a predefined activity FPS‐R mean (SD) where the activity was pain on direct pressure of the growth plate Mean (SD) = 6.1 (1.9)
Intervention group 7: foot orthoses, bracing, taping with sports tape, straps Intervention group 8: footwear or heel cushioning									
Sweeney 2023	Calcaneus	32	NR	Time in barefoot sport (mean (SD) = 13.3 (8.2)	1 (6%)	10.1 (1.6)	Heel strap n = 16	NR	NR
				Time in barefoot sport (mean (SD) = 10.9 (7.3)	2 (12%)	10.6 (1.6)	Heel cushioning n = 16	NR	NR
Intervention group 7: foot orthoses, bracing, taping with sports tape, straps with or without standardising the footwear Intervention group 9: heel lifts with or without standardising the footwear									
Alfaro‐Santafa 2021	Calcaneal	208	NR	NR	85 (82%)	11.1 (1.0) years	Foot orthoses n = 104	1. Self‐reported overall pain VAS (mm): Mean (SD) 80.1 (13.1)	2. Self‐reported pain experienced during a predefined activity Pain threshold (kgf) Mean (SD) = 2.9 (0.4)
					88 (88%)	11.1 (1.0) years	Heel lifts n = 104	1. Self‐reported overall pain VAS (mm) Mean (SD) = 81.3 (13.2)	2. Self‐reported pain experienced during a predefined activity Pain threshold (kgf) Mean (SD) = 2.7 (0.4)
James 2016	Calcaneal	124	9.36 (6.36) (Months	NR	16 (52%)	10.8 (1.41)	Foot orthoses n = 62	1. Self‐reported overall pain measured Mean (SD) = 4.2 (1.1) 2. Self‐reported physical function measured by the Oxford Ankle foot questionnaire ‐ Physical domain Median (IQR) = 50.0 (33.3 to 62.5)	NA
			11.52 (9.00) (months)		22 (71%)	10.61 (1.36)	Heel lifts n = 62	1. Self‐reported overall pain measured mean (SD) 4.6 (1.3) 2. Self‐reported physical function measured by the Oxford Ankle foot questionnaire ‐ Physical domain Median (IQR) = 41.7 (33.3 to 62.5)	
Perhamre 2011a	Calcaneal	48	Weeks (range) = 12 (4 to 24)	All regularly participated in sport at Engstom's five level activity	48 (100%)	Median (range) 12 (9 to 14)	Customised thermoplastic heel cup n = 20	NA	2. Self‐reported pain experienced during a predefined activity ‐ Borg's CR‐10 Median (IQR) = 4.5 (4 to 5.5)
			Weeks (range = 8 (4 to 25)			12 (10‐14)	Heel lifts n = 24		2. Self‐reported pain experienced during a predefined activity ‐ Borg's CR‐10 Median (IQR) = 4 (3 to 5.25)

N: number; NR: not reported; NA: not applicable; IQR: interquartile range

##### Participant characteristics

There were a total of 654 children included, with mean ages ranging from 10.3 years to 13.3 years. Most participants had calcaneal apophysitis (n = 562 (86%)).

Participants were mostly boys (n = 480 (73%)). This was influenced by seven studies with boys exceeding 75% of the total population (Alfaro‐Santafa 2021; Kuyucu 2017; Nakase 2020; Perhamre 2011a; Perhamre 2012; Topol 2011; Wiegerinck 2016). There was only one study with more female participants (Sweeney 2023), which was focused on barefoot sport (gymnastics).

Six studies relied on a health professional diagnosis confirmation as part of the study (James 2016; Kuyucu 2017; Perhamre 2012; Reesman 2024; Sweeney 2023; Wiegerinck 2016), while the inclusion criteria of two studies required medical imaging (Alfaro‐Santafa 2021, Kuyucu 2017). The remaining four studies did not provide information about who made the diagnosis or undertook the screening for entry into the study.

##### Types of interventions

There were five different intervention groups of treatments included within the studies:

1. pharmaceutical interventions;
2. foot orthoses, bracing, taping with sports tape, straps;
3. footwear or heel cushioning;
4. heel lifts;
5. exercise.

Half of the studies had a placebo/no treatment arm (Kuyucu 2017; Nakase 2020; Perhamre 2012; Reesman 2024; Topol 2011), two of which had both a placebo arm and no treatment arm (Reesman 2024; Topol 2011). There was a three‐arm trial that compared two treatments with a no‐treatment arm (Wiegerinck 2016) and four other studies that compared two different treatments (Alfaro‐Santafa 2021; Perhamre 2011a; Sweeney 2023; James 2016).

Table provides details of how each of the interventions or control conditions were described in the studies, including any additional treatment instructions provided to the children or co‐interventions.

**4:** Table 4. Intervention descriptions

Trial	Intervention detail	Comparison/s	Profession/training providing intervention	Setting	Specific information about intervention/s	Dose/frequency	Co‐interventions/ advice
Intervention group 6: pharmaceutical interventions versus placebo (1 trial)							
Nakase 2020	1% lidocaine (1 mL) with 20% dextrose (1 mL; dextrose group)	1% lidocaine (1 mL) with saline (1 mL; saline group	Profession not reported/single investigator performed all injections	Hospital	Half of the solution was injected into the deep infrapatellar bursa and infrapatellar fat pad and the remainder was injected into the superficial infrapatellar bursa using ultrasonographic guidance in long axis image.	Injections were administered monthly for 3 months.	No limits on sport applied
Intervention group 7: foot orthoses, bracing, taping with sports tape, straps versus placebo (1 trial)							
Kuyucu 2017	Kinesio taping ‐ applied using a 4 to 6 inch tape portion, cut and pulled from both ends to apply moderate to severe tension (50 to 75%)	Hypafix (BSN Medical Guilllaime Kroll, Luxembourg) hypoallergenic tape, without applying tension	Physiotherapist/ single trained investigator	Not described	Taping was applied in a way to intersect the insertion point of the Achilles tendon to the calcaneus perpendicularly. There was an application of moderate to severe tension (50 to 75%). The knee was placed in extension and the ankle in maximum dorsiflexion during the application.	Applied every 3 days, with 1‐day intervals between two consecutive sessions in order to rest the skin. In this way, a total of 12 kinesio taping procedures were carried out.	Both groups included stretching exercises, topical analgesic treatment, and massage therapy aimed at heel and plantar fascia.
Intervention group 7: foot orthoses, bracing, taping with sports tape, straps versus wait list treatment (1 trial)							
Perhamre 2012	Individually made moulded rigid ‘‘Wessmark’’ cup (image provided)	Without a heel cup	Not described	Sports medicine clinic	Not described	Not described	Not described
Intervention group 6: pharmaceutical interventions versus placebo versus usual care (2 trials)							
Topol 2011	1% lidocaine with 12.5% dextrose (2 ml mL)	1% lidocaine with saline (2 mL; (placebo)/ usual care	Physician performed all injections; physical therapist provided usual care.	Hospital	A single leg squat and palpation were used to mark the most distal and proximal areas of pain/tenderness. Injections were performed with a 27‐gauge needle beginning at the most distal point of tenderness, with insertion to bony depth, and injecting 1⁄2 mL. Injections were repeated at 1 cm intervals, moving proximally for a total of 3 to 4 midline injections. Usual care involved video instructions on stretching and exercise in addition to at least one individual appointment for confirmation of proper exercise performance.	Injections were provided on three occasions in three months (frequency not described) regardless of symptoms.	Acetaminophen as required for postinjection discomfort. Participants were recommended to avoid running or kicking for 1 week after the first injection, and to run as tolerated after the first week. They were also advised not to run or kick for 3 days after both the second and any subsequent injections. No limits were placed on those in usual care.
Reesman 2024	Dexamethasone (DPS 0.75 mg) delivered through iontophoresis	1.5 ml of 0.9% saline solution delivered through iontophoresis/ usual care	Physical therapist	Sports Medicine Physical Therapy centre	The I‐Bresis System and I‐Bresis Patch were used as the delivery system. 1.5 ml of the dexamethasone sodium phosphate (4ml/1mL) was placed on one side of the I‐Bresis patch and 1.5 ml of 0.9% sodium chloride (saline) solution was placed on the other of the patch. The placebo replicated this with both patch sides having 1.5 ml of 0.9% sodium chloride (saline) solution. Usual care included a standard physical therapy protocol for apophysitis of the knee without further details of what this included.	Duration: 123 minutes. Frequency: twice per week for a total of 12 sessions over a maximum of 8 weeks or until Return to Sport criteria were met, whichever was sooner.	Regardless of grouping, all participants received a standard physical therapy protocol for apophysitis of the knee. This included up to 20 visits.
Intervention group 4: exercise Intervention group 9: heel lifts No treatment							
Wiegerinck 2016	Exercises	ViscoHeel (Bauerfeind)/advised to pragmatically cease painful activity and restart when pain subsided	Orthopaedic surgeon/ physical therapist	Paediatric orthopaedic department	Exercises primarily focused on eccentric calf strengthening, in addition to daily “at home” exercises that were not described. ViscoHeel is a viscoelastic silicon heel cushion of unknown thickness and density reported to lift the heel.	No exercise dose provided. Participants were advised to wear the heel cushion daily and during physical activity; it was fitted to all footwear.	No limitations were placed on physical activities.
Intervention group 7: foot orthoses, bracing, taping with sports tape, straps Intervention group 8: footwear or heel cushioning							
Sweeney 2023	X Brace (Tulis)	Cheetah heel cup (Tulis)	Sports medicine physicians	Tertiary care regional hospital	X Brace is an elastic foot brace with an integrated silicone strip on the heel strap that was fitted by the participant. The Cheetah heel cup was a slip‐on neoprene ankle brace with a built‐in multicell, multilayer waffle heel cup without any further description of materials, density, or height.	Participants were recruited to wear devices during all barefoot activities for 3 months.	Participants were also provided with interventions including stretching, heel cups for daily activity (worn in shoes during the day), activity modification recommendations, ice, non‐steriodal anti‐inflammatory drugs, or a combination of all.
Intervention group 7: foot orthoses, bracing, taping with sports tape, straps Intervention group 9: heel lifts							
Alfaro‐Santafa 2021	Customised foot orthoses	Off‐the‐shelf heel lift (8mm)	Podiatrist	Podiatry clinic	Children in treatment A group received custom‐made foot orthoses as treatment intervention. Orthoses were 9 mm thick and were composed of: confortene, polypropylene, poron XRD, and lunasoft (image provided). No additional details on prescription or modifications to device Heel lift was 8 mm, composed of confortene, poron XRD, and ethyl vinyl acetate.	Participants were encouraged to wear interventions for 8 to 10 hours per day for all activities.	All participants were provided with information about triceps surae stretching, and sport activity intensity reduction.
James 2016	Prefabricated orthoses with own or standardised shoes	Heel lifts (6 mm) with own or standardised shoes	Podiatrist	Community health service	Prefabricated orthoses were a Prothotic W (The Orthotic Laboratory), a polyurethane device that aims to invert the rearfoot with a medial varus wedge combined with a small notch in the cuboid area, covered with a 3 mm blown multidensity EVA cover (Multiform). Heel lifts were 6 mm and made from high‐density (PE 180 kg/m^3^) ethylene vinyl acetate (EVA). Footwear was standardised to either the Addidas Supernova, described as having a firm heel counter, dual density EVA midsole and rearfoot control, or own footwear.	Wearing instructions were provided to participants, but these were not described.	Participants were asked to ice the area of pain once a day for a total of 10 minutes during the initial stage of care (1 month). They were also recommended to continue the icing regime after sporting activities until pain free. They were provided with a stretching programme at the 1 month follow‐up appointment consisting of a static weight‐bearing gastrocnemius and soleus stretch with a picture of limb positioning provided.
Perhamre 2011a	Heel cup	Heel lift (5 mm)	Not described	Sports medicine clinic	Heel cup was a rigid thermoplastic cup (of 3 mm height but with no wedge effect) moulded directly on the bare heel with a three‐fourth length arch support. It extended 2 to 3 mm up the heel. Heel lift was a 5 mm cork wedge covered with a thin elastic surface.	Participants wore their allocated intervention for 4 weeks, had a washout period of 2 weeks, then received the alternate intervention for 4 weeks. Participants could then choose their preferred intervention for an additional 12 weeks. No further wear instructions were provided.	Not described

There were two studies using dextrose injections, albeit with different dosages (Nakase 2020; Topol 2011). The one study using exercise as an intervention, provided limited information, describing the intervention only as eccentric exercises without descriptions of frequency, intensity, or timing (Wiegerinck 2016). No study specifically described stretching exercises (guided either by a health professional within the clinical setting or implemented within the home environment) with specific dosage information (i.e. frequency, timing, type), or manual therapy such as dry needling or massage with specific dosage information (i.e. body location, frequency, timing, type). However, stretching exercises were generally described as an adjunct to all participants in four studies (Alfaro‐Santafa 2021; James 2016; Kuyucu 2017; Sweeney 2023), while massage was described as an adjunctive treatment to all participants in one study (Kuyucu 2017). Reesman 2024 also described an adjunctive physical therapy protocol, but without providing content details.

###### Co‐interventions

Two studies by the same author provided no information on any co‐interventions (Perhamre 2011a; Perhamre 2012). Other studies reported no limits on physical activity during participation (Nakase 2020; Wiegerinck 2016), self‐initiated ice application when in pain (James 2016; Sweeney 2023), a physical therapy programme and/or exercises or stretches (Alfaro‐Santafa 2021; James 2016; Kuyucu 2017; Reesman 2024; Sweeney 2023), restriction or modification of physical activities (Alfaro‐Santafa 2021; Sweeney 2023; Topol 2011), or advice on topical or systemic analgesic medication use (Kuyucu 2017; Sweeney 2023; Topol 2011). James 2016 described different types of footwear as co‐interventions without effect. Therefore, we did not consider their impact during analysis. In all other studies, the co‐interventions were not described in any way that we could consider their impact during any analysis.

##### Critical outcomes

###### Self‐reported overall pain

Overall pain was reported in four studies with different interventions (Alfaro‐Santafa 2021; James 2016; Kuyucu 2017; Reesman 2024), with no consistency in outcome time points. Three studies related to calcaneal apophysitis, with James 2016 describing overall pain in the short term only, Alfaro‐Santafa 2021 in the medium term, and Kuyucu 2017 in the short and medium term. Reesman 2024 reported overall pain in relation to traction apophysitis of the tibial tubercle in the short term. Overall pain‐related data were collected with either a visual analogue scale (VAS) (Alfaro‐Santafa 2021; Kuyucu 2017) or Wong‐Baker FACES Pain Rating Scale [79] (James 2016; Reesman 2024). On both scales, the lower score indicates less pain. Only James 2016 described the actual question used to anchor the response relating to overall pain.

###### Self‐reported physical function

Physical function was the most commonly reported critical outcome. This was measured in three studies using the physical domain of the Oxford Ankle Foot Questionnaire for Children [50] (James 2016; Sweeney 2023; Wiegerinck 2016). This tool consists of four separate domains and is not designed to give an overall single score, such as the single overall score reported by Wiegerinck 2016. Each domain is scored out of 100, where higher scores indicate better function relating to the physical, school/play, emotional, or footwear domains. We used the physical domain score where available (James 2016; Sweeney 2023), as per the scoring instructions.

Kuyucu 2017 used the American Orthopedic Foot‐Ankle Society Score [80]. This tool is scored out of 100, where 50 points are assigned to function, 40 points to pain, and 10 points to alignment to give a total score. As this tool has more points assigned to function, we reported the overall score for this outcome. A score of 100 represents no impairment, while a score of zero represents severe impairment.

Reesman 2024 reported physical function using the Lower Extremity Function Scale [48]. This tool focusses questions on activities with increasing demands. It is scored out of 80, where 0 indicates the most severe limitation and 80 indicates having no limitations.

###### Self‐reported participation in sports

Participation in sports data were collected in one study using a return to sport checklist completed by the therapist (Reesman 2024), which used pain during physical activities to measure the number of days for the participant to return to their preferred sport with no or low pain.

###### Total adverse events

Total adverse events were reported as a count in five studies (James 2016; Nakase 2020; Reesman 2024; Sweeney 2023; Wiegerinck 2016).

###### Withdrawals due to adverse events

Nakase 2020 was the only study reporting withdrawals due to the children not wanting another injection, though these were not classified as withdrawals due to adverse events. Withdrawals were described in other studies. However, these were described as lost to follow‐up without reasons and not due to adverse events (Alfaro‐Santafa 2021; James 2016; Reesman 2024; Sweeney 2023; Wiegerinck 2016). There were four studies that reported retaining all participants (Kuyucu 2017; Perhamre 2011a; Perhamre 2012; Topol 2011).

###### Important outcomes

####### Self‐reported treatment success

One study collected information from participants relating to their perception of treatment success through a VAS where 0 mm = very dissatisfied and 100 mm = very satisfied (Wiegerinck 2016).

####### Self‐reported pain experienced during a predefined activity

Seven studies focused on measuring pain only during specific predefined activities (Alfaro‐Santafa 2021; Nakase 2020; Perhamre 2011a; Perhamre 2012; Sweeney 2023; Topol 2011; Wiegerinck 2016). These studies varied in timeframe: Perhamre 2011a and Perhamre 2012 reported outcomes in the short term; Nakase 2020, Sweeney 2023 and Wiegerinck 2016 reported outcomes in the short and medium term; and Alfaro‐Santafa 2021 and Topol 2011 reported outcomes only in the medium term. Activities were predominately related to sport of choice (Nakase 2020; Perhamre 2011a; Perhamre 2012; Topol 2011); however, for two studies, it was pain relating to algometer measurement only, where pain intensity or perception was measured during a predefined pressure application (Alfaro‐Santafa 2021; Wiegerinck 2016). One study used an algometry threshold value as a pseudo‐measure of pain experienced during an activity (Alfaro‐Santafa 2021).

A VAS was used in Sweeney 2023, where 0 indicated no pain, and 100, most pain. This was applied to three activities in this study: pain during sports participation, pain during activities of daily living, and pain during rest. We prioritised reporting pain during sports participation.

The VISA score (without information on the type of VISA score) was used by Nakase 2020. This tool assesses both pain and its impact on physical function. Therefore, we prioritised this tool for this outcome. The Borg CR10 tool [81] was used in two studies to measure perceived pain on a scale of 0 to 10 (Perhamre 2011a; Perhamre 2012). The Wong‐Baker FACES Pain Rating Scale [79] was used in one study for rating pain during algometer testing (Wiegerinck 2016), with a lower score indicating less pain.

One study indicated in their protocol that they would collect binary data on the presence of pain during activity and algometer measurement (Alfaro‐Santafa 2021). Binary pain results were not in the final publication. However, this study also reported overall pain, so we used only overall pain data in the main summary of findings tables.

Lastly, the Nirschl Pain Phase Scale (NPPS) [82] was used by Topol 2011, where a lower score indicated less pain. This is a seven‐level measure of sport inhibition and sport‐related symptoms. NPPS scores between four and seven are observed where sports participation is most impacted by pain.

####### Active range of motion of the relevant lower limb joint

One study measured ankle joint range of motion with the weight‐bearing lunge test in the short term (James 2016).

####### Self‐reported, or parent proxy‐reported, quality of life

No studies measured quality of life outcomes.

#### Excluded studies

We excluded 12 studies at full‐text screening due to their study design (Blumenfeld 2023 [83]; Feyzioğlu 2018 [84]; Gaulrapp 2016 [85]; Gerulis 2004 [86]; Holden 2021 [87]; Perhamre 2011 (b) [88]; Rathleff 2020 [89]; Rathleff 2023 [90]; Shields 2016 [91]; Smith 1977 [92]; University of Delaware 2021 [93]; Vermass 2018 [94];). These were primarily cohort designs, commentaries, or case series. We excluded a further four studies as the participants were ineligible based on our inclusion criteria (Hadell 2015 [95]; Molund 2018 [96]; Selhorst 2023 [97]; Wu 2022 [98]). The remaining three studies were abandoned, or we were unable to obtain data from the lead investigators (Karaczun 2016 [99]; Lucciani 2016 [100]; Universitair Medisch Centrum Groningen 2025 [101]). These are listed in detail in Supplementary material 3.

There are three studies recently completed that have no outcome data. Details of these are listed in Supplementary material 4.

### Risk of bias in included studies

We assessed the included studies using the Cochrane RoB 2 tool. We provided support for our judgements for all outcomes in Supplementary material 5 and full consensus responses to the signalling questions for each domain for outcomes of all studies in Supplementary material 8.

Of four studies reporting overall pain, we judged three to be at high risk of bias (Alfaro‐Santafa 2021; Kuyucu 2017; Reesman 2024). This was because of limited information described in the trial registry, challenges with blinding, small participant numbers, or differences between protocol and published results. We judged the fourth study to be at low risk of bias (James 2016).

Five studies reported physical function outcomes. We judged three at high risk of bias because of limited descriptive information, challenges with blinding, and either the absence of a published protocol or unexplained differences between the published protocol and the reported outcomes (Kuyucu 2017; Reesman 2024; Sweeney 2023). We had some concerns about one study relating to blinding and analysis (Wiegerinck 2016). We judged one study at low risk of bias (James 2016).

One study reported participation in sport, and we judged it to be at high risk of bias because of the limited information described in the trial registry record (Reesman 2024).

Four studies reported adverse events and one also reported withdrawals due to adverse events. We judged two of these at high risk of bias because of the limited information within the trial registry or small participant numbers, and deviations between protocol and publication (Reesman 2024; Sweeney 2023). We had some concerns about two of the studies because of limited information about randomisation or limited outcome information or blinding (Nakase 2020; Wiegerinck 2016). We judged one study to be at low risk of bias (James 2016).

Seven studies reported pain during a predefined activity. We judged two of these studies to be at high risk of bias due to not reporting the predefined outcome planned in the protocol and also because of differences in randomisation, which may have impacted group allocation (Alfaro‐Santafa 2021) or data collection methods and analysis (Sweeney 2023). We had some concerns about five studies (Nakase 2020; Perhamre 2011a; Perhamre 2012; Topol 2011; Wiegerinck 2016). These concerns were because of limited information about randomisation, methods used to collect outcome data, or no published protocol was found to support pre‐planned analysis.

There was one study reporting outcomes of joint range of motion that we rated at low risk of bias (James 2016).

### Synthesis of results

Studies reporting critical outcomes are listed below in each comparison with analysis at each time point in Supplementary material 6.

#### Comparison 1: Pharmaceutical intervention compared to a placebo for children with traction apophysitis of the tibial tubercle

See Table and Supplementary material 9, which describe all outcomes for studies comparing dexamethasone (DPS 0.75 mg) delivered through iontophoresis compared to a placebo and studies comparing 12.5% or 20% dextrose injection in 1% lidocaine injections to a placebo. The critical outcomes reported in this comparison were overall pain, physical function, and participation in sport in the short term, which were reported in one study (Reesman 2024). Adverse events were measured in the short term in two studies (Nakase 2020 ; Reesman 2024). Pain during activity was measured in the medium term in one study (Nakase 2020) and the long term in another study (Topol 2011).

The evidence is very uncertain about whether dexamethasone (DPS 0.75 mg) delivered through iontophoresis impacts overall pain (MD −0.52, 95% CI −1.24 to 0.20; 23 participants; very low‐certainty evidence; Analysis 1.1), physical function (MD −1.76, 95% CI −16.08 to 12.56; 19 participants; very low‐certainty evidence; Analysis 1.2), or participation in sport (MD 7.90, 95% CI −0.41 to 16.21; 16 participants; very low‐certainty evidence; Analysis 1.3) compared to a placebo in the short term for traction apophysitis at the tibial tubercle. We downgraded twice for risk of bias due to evidence being from a single study at high risk of bias, and twice for imprecision as the optimal information size was not reached and the study ceased early. We also downgraded for publication bias as only limited results were available from the online trial registry without information on adherence to the protocol.

The evidence is very uncertain about the adverse effects of pharmaceuticals in the short term (RR 1.31, 95% CI 0.88 to 1.96; 2 studies, 74 participants; very low‐certainty evidence; Figure), with downgrading decisions reported in Figure. A pharmaceutical intervention may result in little to no difference in pain during an activity in the medium term (SMD −0.47, 95% CI −4.91 to 3.97; I² = 60%; 2 studies, 86 participants; low‐certainty evidence; Figure), with downgrading decisions reported in Figure. The evidence is very uncertain about whether 12.5% dextrose in 1% lidocaine injections impact pain during activity in the long term (MD −1.00, 95% CI −1.87 to −0.13; 1 study, 34 participants; very low‐certainty evidence). We downgraded once for risk of bias due to some concerns with trial blinding and downgraded twice for imprecision due to very small participant numbers.

**2:**
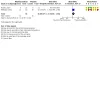


**3:**
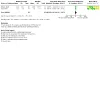


#### Comparison 2: Pharmaceutical intervention compared to usual care for children with traction apophysitis of the tibial tubercle

See Table, and Supplementary material 10, which describe all outcomes for studies comparing dexamethasone (DPS 0.75 mg) delivered through iontophoresis, or a study comparing 12.5% in 1% lidocaine injections to usual care. Usual care was poorly described. The critical outcomes reported in this comparison were overall pain, physical function, and participation in sport in the short term, and adverse outcomes, which were measured in one study (Reesman 2024). Pain during an activity was measured in the medium and long term in one study (Topol 2011).

The evidence is very uncertain about whether dexamethasone (DPS 0.75 mg) delivered through iontophoresis impacts overall pain (MD −0.80, 95% CI −1.73 to 0.13; 21 participants; very low‐certainty evidence; Analysis 2.1), physical function (MD 2.68, 95% CI −17.56 to 22.92; 16 participants; very low‐certainty evidence; Analysis 2.2), or participation in sport (MD 0.85, 95% CI −7.13 to 8.83; 11 participants; very low‐certainty evidence; Analysis 2.3) in the short term compared to usual care. The evidence is very uncertain about the adverse effect of pharmaceuticals in the short term compared to usual care (RR 1.36, 95% CI 0.88 to 2.10; 1 study, 30 participants; very low‐certainty evidence; Analysis 2.4). We downgraded twice for high risk of bias and twice for imprecision as the optimal information size was not reached and the study ceased early. We also downgraded for publication bias as only limited results were available from the online trial registry, without any information on adherence to the protocol.

The evidence is also very uncertain about whether 12.5% dextrose in 1% lidocaine injections impact pain during activity in the medium term (MD −2.40, 95% CI −3.24 to −1.56; 1 study, 43 participants; very low‐certainty evidence; Analysis 2.5) or long term (MD −2.30, 95% CI −3.14 to −1.46; 1 study, 35 participants; very low‐certainty evidence; Analysis 2.6). We downgraded once for risk of bias due to some concerns with trial blinding and twice for imprecision due to very small participant numbers.

#### Comparison 3: Taping compared to a placebo for children with calcaneal apophysitis

See Table and Supplementary material 11, which describe all outcomes for a trial comparing Kinesio tape applied as described in Table versus a non‐stretch tape acting as a placebo. The critical outcomes reported in this comparison were overall pain and physical function in the short term and the medium term (Kuyucu 2017).

The evidence is very uncertain about whether Kinesio tape impacts overall pain in the short term (MD 0.10, 95% CI −1.25 to 1.45; 1 study, 22 participants; very low‐certainty evidence; Analysis 3.1) or medium term (MD −1.00, 95% CI −1.77 to −0.23; 1 study, 22 participants; very low‐certainty evidence; Analysis 3.2), or impacts physical function in the short term (MD 6.10, 95% CI −0.08 to 12.28; 1 study, 22 participants; very low‐certainty evidence; Analysis 3.3) or medium term (MD 11.20, 95% CI 5.69 to 16.71; 1 study, 22 participants; very low‐certainty evidence; Analysis 3.4), compared to a placebo tape. We downgraded twice for risk of bias as this single study had a high risk of bias, and twice for imprecision due to very small participant numbers.

#### Comparison 4: Foot orthoses compared to heel lifts for children with calcaneal apophysitis

See Table and Supplementary material 12, which describe all outcomes for trials comparing prefabricated foot orthoses (James 2016) or customised foot orthoses (Alfaro‐Santafa 2021; Perhamre 2011a) to heel lifts. The critical outcomes reported in this comparison were overall pain and physical function in the short term, and adverse events (James 2016). Overall pain and physical function were also reported in the medium term (Alfaro‐Santafa 2021) and long term (James 2016). Joint range of motion was reported in the short term (James 2016).

Foot orthoses likely do not reduce overall pain in the short term compared to heel lifts (MD 0.00, 95% CI −0.44 to 0.44; 1 study, 123 participants; moderate‐certainty evidence; Analysis 4.1), but the evidence is very uncertain in the medium term (MD −55.70, 95% CI −60.97 to −50.43; 1 study, 208 participants; very low‐certainty evidence; Analysis 4.2). Foot orthoses likely result in little to no difference in physical function in the short term (MD −1.30, 95% CI −7.58 to 4.98; 124 participants; Analysis 4.3) or long term (MD −4.30, 95% CI −10.68 to 2.08; 1 study, 101 participants; moderate‐certainty evidence; Analysis 4.5) compared to heel lifts. Whereas in the medium term, foot orthoses likely result in a slight reduction in physical function compared to heel lifts (MD −7.80, 95% CI −14.22 to −1.38; 1 study, 106 participants; moderate‐certainty evidence; Analysis 4.4). Foot orthoses likely result in little to no difference in joint range of motion in the short term (MD 0.70, 95% CI −0.98 to 2.38; 1 study, 124 participants; moderate‐certainty evidence; Analysis 4.8). There were no reported adverse events from foot orthoses in the long term from one study.

We downgraded the evidence from James 2016 by one level for imprecision due to representing only one study, with results with a narrow confidence interval, and we downgraded the evidence from Alfaro‐Santafa 2021 twice for risk of bias as this single study had a high risk of bias, and for publication bias due to unexplained differences in outcome measure reporting and randomisation differences between protocol and publication.

##### Comparison 5: Heel cushioning compared to heel straps for children with calcaneal apophysitis

See Table and Supplementary material 13, which describe all outcomes for the study comparing heel cushioning to heel straps (Sweeney 2023). The critical outcomes reported in this comparison were physical function in the short term and adverse events. Physical function was also reported in the medium term, along with pain during activity in the short term and medium term.

The evidence is very uncertain about the effect of heel cushioning on physical function in the short term compared to a heel strap (MD −2.00, 95% CI −12.48 to 8.48; 1 study, 43 participants; very low‐certainty evidence; Analysis 5.1) or medium term (MD 2.00, 95% CI −7.02 to 11.02; 1 study, 43 participants; very low‐certainty evidence; Analysis 5.2), or on pain during activity in the short term (MD 1.10, 95% CI −0.03 to 2.23; 1 study, 43 participants; Analysis 5.4) or medium term (MD −0.20, 95% CI −1.61 to 1.21; 1 study, 43 participants; very low‐certainty evidence; Analysis 5.5). There were no adverse events reported (Analysis 5.3). We downgraded twice for high risk of bias in this single study and twice for imprecision due to very small participant numbers.

##### Additional comparisons of important outcomes

Additional summary of findings tables for studies that reported only our important outcomes are provided as supplementary materials: heel lifts verus no treatment (Supplementary material 14); foot orthoses versus no treatment (Supplementary material 15); exercise versus no treatment (Supplementary material 16); and exercise versus heel lifts (Supplementary material 17).

#### Equity assessment

We identified greater study recruitment of male participants within this review, in addition to a larger number of children participating in sport at a high level of competition or frequency greater than community‐based sport. However, we did not investigate equity‐related characteristics in this review.

#### Reporting biases

We used a comprehensive search strategy for databases and clinical trial registries. As only a limited number of studies were identified, we interpreted the findings cautiously and performed descriptive analyses.

We were unable to determine publication bias through the creation of funnel plots due to the limited number of studies and heterogeneity in the reported outcomes and timeframes.

## Discussion

### Summary of main results

We evaluated the broad impact of treatment options that have been researched for lower limb apophysitis conditions in 654 children. Most of these trials focused on calcaneal apophysitis (562 participants, 86% of total sample), and most were boys (480, 73%). To guide clinical practice, we compared the results from trials comparing interventions to placebo, interventions to waitlist or usual care treatment, and one intervention to another.

We found trials reporting diverse outcome measures. We prioritised reporting critical outcome measures of overall pain, physical function, and participation in sport in this review. Trials measuring these outcomes included a range of interventions: dexamethasone (DPS 0.75 mg) delivered through iontophoresis versus a placebo versus usual care in a 3‐arm trial (1 trial, 45 participants); 20% dextrose in 1% lidocaine to a placebo (1 trial, 38 participants); 12.5% dextrose in 1% lidocaine versus a placebo versus usual care in a three‐arm trial (1 trial, 54 participants); taping versus a placebo (1 trial, 22 participants); foot orthoses versus heel lifts (2 trials, 236 participants); and heel cushioning versus a heel strap (1 trial, 32 participants).

In nearly all interventions, the evidence was very uncertain about the impact of the interventions evaluated on overall pain, physical function, participation in sports activity, pain during activity, or joint range of movement at any time point. However, compared to heel lifts, foot orthoses likely result in little to no difference in overall pain or joint range of motion in the short term or physical function in the short term or long term. Foot orthoses are likely to result in a slight reduction in physical function in the medium term, with no reported adverse events regarding foot orthoses in the long term from one study.

In all studies, it is possible that some of the pain scores reported in the short term were clinically significant. However, these occurred in both groups, potentially because of natural resolution of this condition over time. There are also no agreed minimally important clinical differences in pain or physical function scores for lower limb apophyseal injuries in childhood. We have therefore interpreted the review results cautiously.

### Limitations of the evidence included in the review

We planned to include trials on all lower limb apophyseal injuries. However, no trials were identified investigating treatments for iliac apophysitis, osteochondrosis at the patella–patellar tendon junction (Sinding Larsen Johansson syndrome), or base of the fifth metatarsal traction apophysitis (Iselin’s disease). The included trials varied substantially in the outcomes measured and the timing of outcome assessment, limiting our ability to conduct meta‐analyses. Although we considered grouping assessment time points, we decided against this because these conditions often resolve naturally over time. Consequently, most findings were based on single trials and are reported narratively.

We prioritised reporting the critical outcomes of overall pain, physical function, and participation in sport. Results for all other outcomes and assessment time points are presented in supplementary materials. Given the low to very low certainty of the evidence for the non‐prioritised outcomes, this approach is unlikely to have omitted important findings.

We were unable to undertake any subgroup analysis due to limited data within any of the subgroups. Most of the trials included children who had moderate‐to‐high levels of physical activity, but there were missing data in other trials about the physical activity baseline. It is unknown what impact this may have had on the results or the applicability of the results to children with low‐to‐moderate physical activity levels.

Additionally, there were trials included where the usual care treatment arm was inadequately described (Reesman 2024; Topol 2011), again limiting the interpretation of these results. Commonly, exercise interventions are reported in a way to enable these to be replicated, using checklists such as the TIDieR‐Rehab checklist [102] or the Consensus on Exercise Reporting Template (CERT) [103]. Likewise, customised foot orthoses used in one trial were not described in a way that could be replicated in clinical practice (Alfaro‐Santafa 2021). Without detailed intervention information, it limits a clinician's ability to determine if the intervention or usual care intervention arm is representative of interventions available in different countries or settings.

We commonly downgraded the evidence due to trials having small participant numbers, limited transparency in pre‐registered protocols, limited description of blinding or randomisation, or early stopping.

The absence of sport‐specific populations may also limit the transferability of findings. Furthermore, limited reporting of physical activity levels and maturation stages in the trials prevented potential subgroup analyses, despite the variable activity levels at which these conditions occur.

### Limitations of the review processes

We conducted our search of electronic databases and trial registries without any language restrictions. There were review authors who declared conflicts of interest during the development of the protocol, and in order to minimise bias, they did not screen, extract data, or undertake analysis as per our pre‐protocol plan.

We could not use funnel plots to assess publication bias due to the limited number of included trials. We identified three ongoing trials focusing on traction apophysitis of the tibial tubercle (two studies) and calcaneal apophysitis (one study), that we anticipate will be eligible for inclusion when this review is updated, which will strengthen the certainty of evidence.

### Agreements and disagreements with other studies or reviews

We identified two prior systematic reviews during protocol development [104, 105] and one after our protocol [106]. These reviews also focus on treatments for calcaneal apophysitis or traction apophysitis of the tibial tubercle.

Our review identified a greater number of studies both due to an increase in published trials since prior reviews were published, and because of the holistic approach we took to apophyseal injuries. As our review only focused on randomised controlled trials, it provides extensive details on trial data. We noted these details were lacking in the other reviews due to their inclusion of cohort studies, prospective and retrospective observation studies, and case series.

We also identified risk of bias differences between our review and the most recent review, which was by Hernandez‐Lucas [106]. We reported risk of bias according to guidance in the *Cochrane Handbook*, and we applied GRADE, meaning that our review differs in its certainty of the evidence judgement. We identified that the most recent review applied risk of bias criteria inconsistently, and we were unable to replicate how they came to their judgements when we reviewed the trials together with the published protocols, online pre‐prints, and trial registry information [106].

Like our review, this most recent review highlighted the diversity in outcomes and methodological limitations of the included studies. However, our reviews differed in how the findings were interpreted and reported, reflecting our more cautious assessment of the inconsistency of the evidence and limited certainty of the available data.

## Authors' conclusions

### Implications for practice

There is insufficient evidence from randomised controlled trials to support most of the common treatments used in practice for traction apophysitis of the tibial tubercle or calcaneal apophysitis. Across studies, many outcomes also improved in the control groups (e.g. placebo, usual care) over the short, medium, or long term. The certainty of evidence was mostly very low to low, largely due to small participant numbers and incomplete reporting of trial results.

The inconsistency in outcomes used, variation in outcome reporting, and diversity of interventions, limited our ability to pool results. This also restricted our capability to determine whether observed effects were clinically meaningful. Quality of life outcomes were not used in any trials. This also means that important outcomes may not have been captured.

These observations should be carefully considered by clinicians when deciding whether to implement specific interventions, particularly given the limited and uncertain evidence base. In particular, some of the interventions evaluated are associated with high healthcare utililisation costs, while others impose a substantial time and financial burden on both the healthcare system and affected families.

In light of the limited certainty of evidence supporting any particular intervention, it would not seem unreasonable for clinicians managing apophyseal injuries to prioritise lower‐cost treatment options, especially when anticipated benefits may be similar across interventions, and spontaneous improvement over time is common.

#### Equity‐related implications for practice

It is unknown if there are equity‐related implications relating to clinical practice. Children who play competitive sport may have greater psychological or social impacts during injury. However, these may also be children who have a higher risk tolerance, and greater ability to self‐manage pain and physical function during their training and games, meaning these injuries may have a lower impact on some.

Short‐term injuries and pain for children who play community‐based sport may also result in the child giving up sport or significantly reducing their physical activity in the short term.

The difference in children's tolerance for playing with discomfort or pain that takes them out of play may be different, and this review was unable to provide more information about these circumstances.

Clinicians should carefully consider these observations when interpreting the results of this review.

### Implications for research

Apophyseal injuries are commonly self‐resolving conditions for most children. Despite this, some children report that these injuries impact their quality of life and have long‐term sequelae.

Small participant numbers in the studies included in this review and high risk of bias due to problems in their design limit the strength of the available evidence. Several studies compared one intervention to another, with little or no inclusion of placebo or sham comparison groups.

No studies included quality of life as an outcome measure. The inconsistent use of outcomes is a common issue in paediatric clinical trials, particularly for musculoskeletal conditions associated with chronic pain. Recent work has highlighted the importance of consistent outcome measures being used in trials attempting to evaluate children's report of pain that persists with functional impacts. Outcome measures should reflect the factors that impact children and families, including overall pain, pain interference with activities of daily living, overall wellbeing, emotional functioning, physical functioning, and sleep quality [107].

#### Equity‐related implications for research

Apophyseal injuries are commonly short‐term and self‐resolving. Despite limited findings, this review showed that, regardless of the intervention, there were some positive changes over time in many outcome domains. Given the low to very low certainty of the available evidence, it is uncertain whether any intervention improves or worsens outcomes. Future research should consider the self‐limiting nature of apophyseal injuries when designing studies and selecting participants, particularly focusing children who have substantial functional impairment and therefore may have the greatest potential to benefit from treatment.

We did not consider the financial costs of treatments as we did not find any trials reporting the cost of treatments. All treatments used in trials come with time or financial costs for families and the health system. Some of these will vary drastically based on the equipment and technology used. Future research should consider the costs of interventions, as out‐of‐pocket expenses may affect access to treatment. Identifying effective and cost‐effective interventions could reduce low‐value care and improve equitable access to appropriate management.

## Supporting Information

Supplementary materials are available with the online version of this article: 10.1002/14651858.CD015156.pub2.

Supplementary materials are published alongside the article and contain additional data and information that support or enhance the article. Supplementary materials may not be subject to the same editorial scrutiny as the content of the article and Cochrane has not copyedited, typeset or proofread these materials. The material in these sections has been supplied by the author(s) for publication under a Licence for Publication and the author(s) are solely responsible for the material. Cochrane accordingly gives no representations or warranties of any kind in relation to, and accepts no liability for any reliance on or use of, such material.

**Supplementary material 1** Search strategies

**Supplementary material 2** Characteristics of included studies

**Supplementary material 3** Characteristics of excluded studies

**Supplementary material 4** Characteristics of ongoing studies

**Supplementary material 5** Risk of bias

**Supplementary material 6** Analyses

**Supplementary material 7** Data package

**Supplementary material 8** Consensus responses to the signalling questions for each domain for all extracted outcomes

**Supplementary material 9** Supplementary summary of findings: Pharmaceutical intervention compared to a placebo for children with traction apophysitis of the tibial tubercle for all outcomes

**Supplementary material 10** Supplementary summary of findings: pharmaceutical intervention compared to usual care for children with traction apophysitis of the tibial tubercle for all outcomes

**Supplementary material 11** Supplementary findings tables: Taping compared to placebo for children with calcaneal apophysitis for all outcomes

**Supplementary material 12** Supplementary summary of findings: Foot orthoses compared to heel lifts for children with calcaneal apophysitis for all outcomes

**Supplementary material 13** Supplementary findings tables: Heel cushioning compared to straps for children with calcaneal apophysitis for all outcomes

**Supplementary material 14** Supplementary summary of findings: Heel lifts compared to no treatment for children with calcaneal apophysitis

**Supplementary material 15** Supplementary summary of findings : Foot orthoses compared to no treatment for children with calcaneal apophysitis

**Supplementary material 16** Supplementary summary of findings: Exercise compared to no treatment for children with calcaneal apophysitis

**Supplementary material 17** Supplementary summary of findings: Exercise compared to heel lifts for children with calcaneal apophysitis

## Figures and Tables

**Table:** 

**Date**	**Event**	**Description**
24 September 2026	Amended	Amended intervention description in abstract from 1. Heel cushioning compared to heel brace to 1. Heel cushioning compared to heel straps

## Additional information

### Registration and protocol

Protocol DOI: https://doi.org/10.1002/14651858.CD015156

### Data, code and other materials

The following are available for download for users of the Cochrane Library: full search strategies for each database (Supplementary material 1); full citations of each unique report for all studies included, ongoing, awaiting classification, or excluded at the full‐text screen; study data, including study information, study arms, and study results or test data (Supplementary material 2;Supplementary material 3 ; Supplementary material 4); consensus risk of bias assessments (Supplementary material 5Supplementary material 8); and analysis data, including overall estimates and settings, all outcome measures at all time points, and individual data rows (Supplementary material 6; Supplementary material 7Supplementary material 10; Supplementary material 11; Supplementary material 12; Supplementary material 13; Supplementary material 14; Supplementary material 15; Supplementary material 16; Supplementary material 17; Supplementary material 9/).

We conducted analyses and data management using RevMan [57]. Template data extraction forms from Covidence can be made available by the review authors on reasonable request.

### History

Protocol first published: Issue 12, 2023 Review first published: Issue 7, 2026
