# Supplementary material for: Non‐surgical treatment for lower limb apophyseal injuries
Source: Cochrane Database Syst Rev. 2026 Jul 15;2026(7):CD015156. doi: 10.1002/14651858.CD015156.pub2 (PMC13370774; doi:10.1002/14651858.CD015156.pub2)
Supplement: Supplementary file 1 — Supplementary material 1 Search strategies [file CD015156-SUP-01-searchStrategy.html]

Search strategies


# Supplementary material 1 to: Non-surgical treatment for lower limb apophyseal injuries

Williams CM, Krommes K, Paterson KL, Haines T, Caserta A, Thorborg K
  
https://doi.org/10.1002/14651858.CD015156.pub2

The material in this section has been supplied by the author(s) for publication under a Licence for Publication and the author(s) are solely responsible for the material. Cochrane has peer reviewed this material in accordance with its editorial policies, but Cochrane has not copyedited, formatted or proofread. Cochrane accordingly gives no representations or warranties of any kind in relation to, and accepts no liability for any reliance on or use of, such material.

Back to top

# Search strategies

## Search strategy

**Cochrane Central Register of Controlled Trials (CENTRAL; 2025, Issue 1) via Ovid; MEDLINE Ovid (1966 to 4th Jan, 2025); Embase Ovid (1980 to 4th Jan, 2025)**

1) Detailed, transparent format

Reproduction of the line history as provided, including Ovid field expansions.

|  |  |
| --- | --- |
| **Line** | **Search statement** |
| **1** | "Iselin".mp. [mp=title, book title, abstract, original title, name of substance word, subject heading word, floating sub-heading word, keyword heading word, organism supplementary concept word, protocol supplementary concept word, rare disease supplementary concept word, unique identifier, synonyms, population supplementary concept word, anatomy supplementary concept word] |
| **2** | "fifth metatars\*".mp. [mp=title, book title, abstract, original title, name of substance word, subject heading word, floating sub-heading word, keyword heading word, organism supplementary concept word, protocol supplementary concept word, rare disease supplementary concept word, unique identifier, synonyms, population supplementary concept word, anatomy supplementary concept word] |
| **3** | "5th metatars\*".mp. [mp=title, book title, abstract, original title, name of substance word, subject heading word, floating sub-heading word, keyword heading word, organism supplementary concept word, protocol supplementary concept word, rare disease supplementary concept word, unique identifier, synonyms, population supplementary concept word, anatomy supplementary concept word] |
| **4** | 2 or 3 |
| **5** | osteochondro\*.mp. [mp=title, book title, abstract, original title, name of substance word, subject heading word, floating sub-heading word, keyword heading word, organism supplementary concept word, protocol supplementary concept word, rare disease supplementary concept word, unique identifier, synonyms, population supplementary concept word, anatomy supplementary concept word] |
| **6** | Osteochondrosis.sh. |
| **7** | apophys\*.mp. [mp=title, book title, abstract, original title, name of substance word, subject heading word, floating sub-heading word, keyword heading word, organism supplementary concept word, protocol supplementary concept word, rare disease supplementary concept word, unique identifier, synonyms, population supplementary concept word, anatomy supplementary concept word] |
| **8** | limit 7 to abstracts |
| **9** | "physeal".mp. [mp=title, book title, abstract, original title, name of substance word, subject heading word, floating sub-heading word, keyword heading word, organism supplementary concept word, protocol supplementary concept word, rare disease supplementary concept word, unique identifier, synonyms, population supplementary concept word, anatomy supplementary concept word] |
| **10** | limit 9 to abstracts |
| **11** | physis.mp. [mp=title, book title, abstract, original title, name of substance word, subject heading word, floating sub-heading word, keyword heading word, organism supplementary concept word, protocol supplementary concept word, rare disease supplementary concept word, unique identifier, synonyms, population supplementary concept word, anatomy supplementary concept word] |
| **12** | limit 11 to abstracts |
| **13** | 5 or 6 or 8 or 10 or 12 |
| **14** | injur\*.mp. [mp=title, book title, abstract, original title, name of substance word, subject heading word, floating sub-heading word, keyword heading word, organism supplementary concept word, protocol supplementary concept word, rare disease supplementary concept word, unique identifier, synonyms, population supplementary concept word, anatomy supplementary concept word] |
| **15** | limit 14 to abstracts |
| **16** | 4 and 13 and 14 |
| **17** | 1 or 16 |
| **18** | "Severs".mp. [mp=title, book title, abstract, original title, name of substance word, subject heading word, floating sub-heading word, keyword heading word, organism supplementary concept word, protocol supplementary concept word, rare disease supplementary concept word, unique identifier, synonyms, population supplementary concept word, anatomy supplementary concept word] |
| **19** | "Sever's".mp. [mp=title, book title, abstract, original title, name of substance word, subject heading word, floating sub-heading word, keyword heading word, organism supplementary concept word, protocol supplementary concept word, rare disease supplementary concept word, unique identifier, synonyms, population supplementary concept word, anatomy supplementary concept word] |
| **20** | 18 or 19 |
| **21** | calcane\*.mp. [mp=title, book title, abstract, original title, name of substance word, subject heading word, floating sub-heading word, keyword heading word, organism supplementary concept word, protocol supplementary concept word, rare disease supplementary concept word, unique identifier, synonyms, population supplementary concept word, anatomy supplementary concept word] |
| **22** | limit 21 to abstracts |
| **23** | heel.mp. [mp=title, book title, abstract, original title, name of substance word, subject heading word, floating sub-heading word, keyword heading word, organism supplementary concept word, protocol supplementary concept word, rare disease supplementary concept word, unique identifier, synonyms, population supplementary concept word, anatomy supplementary concept word] |
| **24** | limit 23 to abstracts |
| **25** | 22 or 24 |
| **26** | 8 or 10 or 12 |
| **27** | children.mp. [mp=title, book title, abstract, original title, name of substance word, subject heading word, floating sub-heading word, keyword heading word, organism supplementary concept word, protocol supplementary concept word, rare disease supplementary concept word, unique identifier, synonyms, population supplementary concept word, anatomy supplementary concept word] |
| **28** | limit 27 to abstracts |
| **29** | adolesc\*.mp. [mp=title, book title, abstract, original title, name of substance word, subject heading word, floating sub-heading word, keyword heading word, organism supplementary concept word, protocol supplementary concept word, rare disease supplementary concept word, unique identifier, synonyms, population supplementary concept word, anatomy supplementary concept word] |
| **30** | limit 29 to abstracts |
| **31** | 28 or 30 |
| **32** | "heel pain".mp. [mp=title, book title, abstract, original title, name of substance word, subject heading word, floating sub-heading word, keyword heading word, organism supplementary concept word, protocol supplementary concept word, rare disease supplementary concept word, unique identifier, synonyms, population supplementary concept word, anatomy supplementary concept word] |
| **33** | limit 32 to abstracts |
| **34** | 31 and 33 |
| **35** | 15 and 25 and 26 |
| **36** | 20 or 34 or 35 |
| **37** | Iliac.mp. [mp=title, book title, abstract, original title, name of substance word, subject heading word, floating sub-heading word, keyword heading word, organism supplementary concept word, protocol supplementary concept word, rare disease supplementary concept word, unique identifier, synonyms, population supplementary concept word, anatomy supplementary concept word] |
| **38** | limit 37 to abstracts |
| **39** | "ASIS".mp. [mp=title, book title, abstract, original title, name of substance word, subject heading word, floating sub-heading word, keyword heading word, organism supplementary concept word, protocol supplementary concept word, rare disease supplementary concept word, unique identifier, synonyms, population supplementary concept word, anatomy supplementary concept word] |
| **40** | limit 39 to abstracts |
| **41** | 38 or 40 |
| **42** | 6 or 26 |
| **43** | 15 and 42 |
| **44** | 41 and 42 and 43 |
| **45** | Osgood\*.mp. [mp=title, book title, abstract, original title, name of substance word, subject heading word, floating sub-heading word, keyword heading word, organism supplementary concept word, protocol supplementary concept word, rare disease supplementary concept word, unique identifier, synonyms, population supplementary concept word, anatomy supplementary concept word] |
| **46** | schlatter\*.mp. [mp=title, book title, abstract, original title, name of substance word, subject heading word, floating sub-heading word, keyword heading word, organism supplementary concept word, protocol supplementary concept word, rare disease supplementary concept word, unique identifier, synonyms, population supplementary concept word, anatomy supplementary concept word] |
| **47** | 45 or 46 |
| **48** | tubercl\*.mp. [mp=title, book title, abstract, original title, name of substance word, subject heading word, floating sub-heading word, keyword heading word, organism supplementary concept word, protocol supplementary concept word, rare disease supplementary concept word, unique identifier, synonyms, population supplementary concept word, anatomy supplementary concept word] |
| **49** | limit 48 to abstracts |
| **50** | tuberosit\*.mp. [mp=title, book title, abstract, original title, name of substance word, subject heading word, floating sub-heading word, keyword heading word, organism supplementary concept word, protocol supplementary concept word, rare disease supplementary concept word, unique identifier, synonyms, population supplementary concept word, anatomy supplementary concept word] |
| **51** | limit 50 to abstracts |
| **52** | 49 or 51 |
| **53** | Knee.mp. [mp=title, book title, abstract, original title, name of substance word, subject heading word, floating sub-heading word, keyword heading word, organism supplementary concept word, protocol supplementary concept word, rare disease supplementary concept word, unique identifier, synonyms, population supplementary concept word, anatomy supplementary concept word] |
| **54** | limit 53 to abstracts |
| **55** | 52 and 54 |
| **56** | 13 and 15 |
| **57** | 55 and 56 |
| **58** | 47 or 57 |
| **59** | "Sinding Larsen".mp. [mp=title, book title, abstract, original title, name of substance word, subject heading word, floating sub-heading word, keyword heading word, organism supplementary concept word, protocol supplementary concept word, rare disease supplementary concept word, unique identifier, synonyms, population supplementary concept word, anatomy supplementary concept word] |
| **60** | "Sinding-Larsen".mp. [mp=title, book title, abstract, original title, name of substance word, subject heading word, floating sub-heading word, keyword heading word, organism supplementary concept word, protocol supplementary concept word, rare disease supplementary concept word, unique identifier, synonyms, population supplementary concept word, anatomy supplementary concept word] |
| **61** | "Sinding-Larson".mp. [mp=title, book title, abstract, original title, name of substance word, subject heading word, floating sub-heading word, keyword heading word, organism supplementary concept word, protocol supplementary concept word, rare disease supplementary concept word, unique identifier, synonyms, population supplementary concept word, anatomy supplementary concept word] |
| **62** | "Sinding Larson".mp. [mp=title, book title, abstract, original title, name of substance word, subject heading word, floating sub-heading word, keyword heading word, organism supplementary concept word, protocol supplementary concept word, rare disease supplementary concept word, unique identifier, synonyms, population supplementary concept word, anatomy supplementary concept word] |
| **63** | 59 or 60 or 61 or 62 |
| **64** | inferior.mp. [mp=title, book title, abstract, original title, name of substance word, subject heading word, floating sub-heading word, keyword heading word, organism supplementary concept word, protocol supplementary concept word, rare disease supplementary concept word, unique identifier, synonyms, population supplementary concept word, anatomy supplementary concept word] |
| **65** | limit 64 to abstracts |
| **66** | distal\*.mp. [mp=title, book title, abstract, original title, name of substance word, subject heading word, floating sub-heading word, keyword heading word, organism supplementary concept word, protocol supplementary concept word, rare disease supplementary concept word, unique identifier, synonyms, population supplementary concept word, anatomy supplementary concept word] |
| **67** | limit 66 to abstracts |
| **68** | apex.mp. [mp=title, book title, abstract, original title, name of substance word, subject heading word, floating sub-heading word, keyword heading word, organism supplementary concept word, protocol supplementary concept word, rare disease supplementary concept word, unique identifier, synonyms, population supplementary concept word, anatomy supplementary concept word] |
| **69** | limit 68 to abstracts |
| **70** | pole.mp. [mp=title, book title, abstract, original title, name of substance word, subject heading word, floating sub-heading word, keyword heading word, organism supplementary concept word, protocol supplementary concept word, rare disease supplementary concept word, unique identifier, synonyms, population supplementary concept word, anatomy supplementary concept word] |
| **71** | limit 70 to abstracts |
| **72** | 65 or 67 or 69 or 71 |
| **73** | 56 and 72 |
| **74** | 63 or 73 |
| **75** | "Clinical Trials as Topic".sh. |
| **76** | "controlled clinical trial".pt. |
| **77** | "randomized controlled trial".pt. |
| **78** | "trial".ti. |
| **79** | "randomized".mp. [mp=title, book title, abstract, original title, name of substance word, subject heading word, floating sub-heading word, keyword heading word, organism supplementary concept word, protocol supplementary concept word, rare disease supplementary concept word, unique identifier, synonyms, population supplementary concept word, anatomy supplementary concept word] |
| **80** | limit 79 to abstracts |
| **81** | "groups".mp. [mp=title, book title, abstract, original title, name of substance word, subject heading word, floating sub-heading word, keyword heading word, organism supplementary concept word, protocol supplementary concept word, rare disease supplementary concept word, unique identifier, synonyms, population supplementary concept word, anatomy supplementary concept word] |
| **82** | limit 81 to abstracts |
| **83** | "quasi\*".mp. [mp=title, book title, abstract, original title, name of substance word, subject heading word, floating sub-heading word, keyword heading word, organism supplementary concept word, protocol supplementary concept word, rare disease supplementary concept word, unique identifier, synonyms, population supplementary concept word, anatomy supplementary concept word] |
| **84** | limit 83 to abstracts |
| **85** | "placebo".mp. [mp=title, book title, abstract, original title, name of substance word, subject heading word, floating sub-heading word, keyword heading word, organism supplementary concept word, protocol supplementary concept word, rare disease supplementary concept word, unique identifier, synonyms, population supplementary concept word, anatomy supplementary concept word] |
| **86** | limit 85 to abstracts |
| **87** | "randomly".mp. [mp=title, book title, abstract, original title, name of substance word, subject heading word, floating sub-heading word, keyword heading word, organism supplementary concept word, protocol supplementary concept word, rare disease supplementary concept word, unique identifier, synonyms, population supplementary concept word, anatomy supplementary concept word] |
| **88** | limit 87 to abstracts |
| **89** | 75 or 76 or 77 or 78 or 80 or 82 or 84 or 86 or 88 |
| **90** | 17 or 36 or 44 or 58 or 74 |
| **91** | 89 and 90 |

2) Reproducible, simplified format

Same line history, expressed as operational steps in the Ovid interface.

For free-text searches, the user selected Abstract under Limits before running the search. For subject headings, the user selected Map Term to Subject Heading. For lines that show a limit being applied, Ovid created the new set by applying the Abstracts limit to the referenced prior set.

Other line types: pt selects Publication Type, ti searches the Title field.

|  |  |
| --- | --- |
| **Line** | **Search terms and how to apply them** |
| **1** | "Iselin" select Abstract under Limits |
| **2** | "fifth metatars\*" select Abstract under Limits |
| **3** | "5th metatars\*" select Abstract under Limits |
| **4** | 2 or 3 |
| **5** | osteochondro\* select Abstract under Limits |
| **6** | Osteochondrosis select Map Term to Subject Heading |
| **7** | apophys\*.mp. select Abstract under Limits |
| **8** | Limit on Abstracts set for line 7 by Ovid |
| **9** | "physeal" select Abstract under Limits |
| **10** | Limit on Abstracts set for line 9 by Ovid |
| **11** | physis select Abstract under Limits |
| **12** | Limit on Abstracts set for line 11 by Ovid |
| **13** | 5 or 6 or 8 or 10 or 12 |
| **14** | injur\* select Abstract under Limits |
| **15** | Limit on Abstracts set for line 14 by Ovid |
| **16** | 4 and 13 and 14 |
| **17** | 1 or 16 |
| **18** | "Severs" select Abstract under Limits |
| **19** | "Sever's" select Abstract under Limits |
| **20** | 18 or 19 |
| **21** | calcane\* select Abstract under Limits |
| **22** | Limit on Abstracts set for line 21 by Ovid |
| **23** | heel select Abstract under Limits |
| **24** | Limit on Abstracts set for line 23 by Ovid |
| **25** | 22 or 24 |
| **26** | 8 or 10 or 12 |
| **27** | children select Abstract under Limits |
| **28** | Limit on Abstracts set for line 27 by Ovid |
| **29** | adolesc\* select Abstract under Limits |
| **30** | Limit on Abstracts set for line 29 by Ovid |
| **31** | 28 or 30 |
| **32** | "heel pain" select Abstract under Limits |
| **33** | Limit on Abstracts set for line 32 by Ovid |
| **34** | 31 and 33 |
| **35** | 15 and 25 and 26 |
| **36** | 20 or 34 or 35 |
| **37** | Iliac select Abstract under Limits |
| **38** | Limit on Abstracts set for line 37 by Ovid |
| **39** | "ASIS" select Abstract under Limits |
| **40** | Limit on Abstracts set for line 39 by Ovid |
| **41** | 38 or 40 |
| **42** | 6 or 26 |
| **43** | 15 and 42 |
| **44** | 41 and 42 and 43 |
| **45** | Osgood\* select Abstract under Limits |
| **46** | schlatter\* select Abstract under Limits |
| **47** | 45 or 46 |
| **48** | tubercl\* select Abstract under Limits |
| **49** | Limit on Abstracts set for line 48 by Ovid |
| **50** | tuberosit\* select Abstract under Limits |
| **51** | Limit on Abstracts set for line 50 by Ovid |
| **52** | 49 or 51 |
| **53** | Knee select Abstract under Limits |
| **54** | Limit on Abstracts set for line 53 by Ovid |
| **55** | 52 and 54 |
| **56** | 13 and 15 |
| **57** | 55 and 56 |
| **58** | 47 or 57 |
| **59** | "Sinding Larsen" select Abstract under Limits |
| **60** | "Sinding-Larsen" select Abstract under Limits |
| **61** | "Sinding-Larson" select Abstract under Limits |
| **62** | "Sinding Larson" select Abstract under Limits |
| **63** | 59 or 60 or 61 or 62 |
| **64** | inferior select Abstract under Limits |
| **65** | Limit on Abstracts set for line 64 by Ovid |
| **66** | distal\* select Abstract under Limits |
| **67** | Limit on Abstracts set for line 66 by Ovid |
| **68** | apex select Abstract under Limits |
| **69** | Limit on Abstracts set for line 68 by Ovid |
| **70** | pole select Abstract under Limits |
| **71** | Limit on Abstracts set for line 70 by Ovid |
| **72** | 65 or 67 or 69 or 71 |
| **73** | 56 and 72 |
| **74** | 63 or 73 |
| **75** | "Clinical Trials as Topic" select Map Term to Subject Heading |
| **76** | "controlled clinical trial" select Publication Type |
| **77** | "randomized controlled trial" select Publication Type |
| **78** | "trial" search Title field |
| **79** | "randomized" select Abstract under Limits |
| **80** | Limit on Abstracts set for line 79 by Ovid |
| **81** | "groups" select Abstract under Limits |
| **82** | Limit on Abstracts set for line 81 by Ovid |
| **83** | "quasi\*" select Abstract under Limits |
| **84** | Limit on Abstracts set for line 83 by Ovid |
| **85** | "placebo" select Abstract under Limits |
| **86** | Limit on Abstracts set for line 85 by Ovid |
| **87** | "randomly" select Abstract under Limits |
| **88** | Limit on Abstracts set for line 87 by Ovid |
| **89** | 75 or 76 or 77 or 78 or 80 or 82 or 84 or 86 or 88 |
| **90** | 17 or 36 or 44 or 58 or 74 |
| **91** | 89 and 90 |

**CINAHL Plus (1937 to 4th Jan, 2025)**

1. **TX Iselin**
2. TX fifth metatars\* or 5th metatars\*
3. TX osteochondro\*
4. MH Osteochondrosis
5. TI apophys\* OR
6. AB apophys\*
7. TI physeal
8. AB physeal
9. TI physis
10. AB physis
11. 3 OR 4 OR 5 OR 6 OR 7 OR 8 OR 9 OR 10
12. TI injur\*
13. AB injur\*
14. 12 OR 13
15. **2 AND 11 AND 14**
16. TX Severs
17. TX Sever's
18. **16 OR 17**
19. TI calcane\*
20. AB calcane\*
21. TI heel
22. AB heel
23. 19 OR 20 OR 21 OR 22
24. TI apophys\*
25. AB apophys\*
26. TI physeal
27. AB physeal
28. TI physis
29. AB physis
30. 24 OR 25 OR 26 OR 27 OR 28 OR 29
31. TI injur\*
32. AB injur\*
33. 31 or 32
34. **23 AND 30 AND 33**
35. TI Heel pain
36. AB Heel pain
37. 35 OR 36
38. TI children
39. AB children
40. TI adolesc\*
41. AB adolesc\*
42. 38 OR 39 OR 40 OR41
43. **37 AND 42**
44. TI iliac
45. AB iliac
46. TI ASIS
47. AB ASIS
48. 44 OR 45 OR 46 OR 47
49. TI apophys\*
50. AB apophys\*
51. MH Osteochondrosis
52. TI physeal
53. AB physeal
54. TI physis
55. AB physis
56. 49 OR 50 OR 51 OR 52 OR 53 OR 54 OR 55
57. TI injur\*
58. AB injur\*
59. 57 OR 58
60. **48 AND 56 AND 59**
61. TX Osgood\*
62. TX schlatter\*
63. **60 OR 61**
64. TI tubercl\*
65. AB tubercl\*
66. TI tuberosit\*
67. AB tuberosit\*
68. 64 OR 65 OR 66 OR 67
69. TI knee
70. AB knee
71. 69 OR 70
72. 68 AND 71
73. TI osteochondro\*
74. AB osteochondro\*
75. TX Osteochondrosis
76. TI apophys\*
77. AB apophys\*
78. TI physeal
79. AB physeal
80. TI physis
81. AB physis
82. 73 OR 74 OR 75 OR 76 OR 77 OR 78 OR 79 OR 80 OR 81
83. TI injur\*
84. AB injur\*
85. 83 OR 84
86. **72 AND 82 AND 85**
87. TX Sinding Larsen
88. TX Sinding‐Larsen
89. TX Sinding Larson
90. TX Sinding‐Larson
91. **87 OR 88 OR 89 OR 90**
92. TI patella\*
93. AB patella\*
94. 92 OR 93
95. TI inferior
96. AB inferior
97. TI distal\*
98. AB distal\*
99. TI apex
100. AB apex
101. TI pole
102. AB pole
103. 95 OR 96 OR 97 OR 98 OR 99 OR 100 OR 101 OR 102
104. TI osteochondro\*
105. AB osteochondro\*
106. MH Osteochondrosis
107. TI apophys\*
108. AB apophys\*
109. TI physeal
110. AB physeal
111. TI physis
112. AB physis
113. 104 OR 105 OR 106 OR 107 OR 108 OR 109 OR 110 OR 111 OR 112
114. TI injur\*
115. AB injur\*
116. 114 OR 115
117. **94 AND 103 AND 113 AND 116**
118. **1 OR 15 OR 18 OR 34 OR 43 OR 60 OR 63 OR 86 OR 91 OR 117**
119. MH Clinical Trials
120. TI trial
121. TI randomi\*ed
122. AB randomi\*ed
123. 121 OR 122
124. TI groups
125. AB groups
126. 124 OR 125
127. TI quasi\*
128. AB quasi\*
129. 127 OR 128
130. TI placebo
131. AB placebo
132. 127 OR 128
133. TI randomly
134. AB randomly
135. 133 OR 135
136. **119 OR 120 OR 123 OR 126 OR 129 OR 132 OR 135**
137. **118 AND 136**

**Clinicaltrials.gov**

Other terms: Iselin disease OR (fifth metatarsal OR 5th metatarsal) OR Sever’s disease OR Severs OR (calcaneal apophysitis OR heel pain OR calcaneus) OR (Osgood-Schlatter OR Osgood OR Schlatter OR tibial tubercle OR tibial tuberosity OR knee) OR (Sinding-Larsen OR Sinding Larsen OR Sinding-Larson OR Sinding Larson OR Larsen-Johansson OR patella OR patellar apex OR inferior pole OR distal patella) OR (iliac crest OR ASIS OR anterior superior iliac spine) OR apophysitis OR physeal OR physis OR osteochondrosis OR osteochondritis OR growth plate injury | Child (birth - 17) | Interventional studies

**WHO ICTRP**

(Iselin OR "fifth metatarsal" OR "5th metatarsal") OR (Sever OR "Sever's disease" OR "calcaneal apophysitis" OR "heel pain" OR calcaneus) OR ("Osgood-Schlatter" OR Osgood OR Schlatter OR "tibial tubercle" OR "tibial tuberosity" OR knee) OR ("Sinding Larsen" OR "Sinding-Larsen" OR "Sinding Larson" OR "Sinding-Larson" OR "Larsen-Johansson" OR patella OR "patellar apex" OR "inferior pole" OR "distal patella") OR ("iliac crest" OR ASIS OR "anterior superior iliac spine") OR apophysitis OR physeal OR physis OR osteochondrosis OR osteochondritis OR "growth plate injury" | In clinical trials in children
