# Supplementary material for: Non‐surgical treatment for lower limb apophyseal injuries
Source: Cochrane Database Syst Rev. 2026 Jul 15;2026(7):CD015156. doi: 10.1002/14651858.CD015156.pub2 (PMC13370774; doi:10.1002/14651858.CD015156.pub2)
Supplement: Supplementary file 2 — Supplementary material 2 Characteristics of included studies [file CD015156-SUP-02-characteristicsOfIncludedStudies.html]

Characteristics of included studies


# Supplementary material 2 to: Non-surgical treatment for lower limb apophyseal injuries

Williams CM, Krommes K, Paterson KL, Haines T, Caserta A, Thorborg K
  
https://doi.org/10.1002/14651858.CD015156.pub2

The material in this section has been supplied by the author(s) for publication under a Licence for Publication and the author(s) are solely responsible for the material. Cochrane has reviewed this material, but Cochrane has not copyedited, formatted or proofread. Cochrane accordingly gives no representations or warranties of any kind in relation to, and accepts no liability for any reliance on or use of, such material.

Back to top

# Characteristics of included studies

## Table of contents

- Studies ordered by Study ID
  - Alfaro-Santafa 2021
  - James 2016
  - Kuyucu 2017
  - Nakase 2020
  - Perhamre 2011a
  - Perhamre 2012
  - Reesman 2024
  - Sweeney 2023
  - Topol 2011
  - Wiegerinck 2016
- Footnotes
- References to studies

## Studies ordered by Study ID

Alfaro-Santafa 2021

| Methods | Randomised controlled trial (parallel group) |
| Participants | **Country of study:** Spain  **Sample size**: 208  **Sex:** 35 female, 173 male  **Total group age (mean (sd))**: 11.2 (1.0) years  **Apophysitis type:** Calcaneal  **Inclusion criteria:** Physically active, aged between 9 and 12 years old and diagnosed radiologically with calcaneal osteochondritis in one foot  **Exclusion criteria:** Heel trauma in the past 2 months; had received anti-inflammatory drugs and/or physical treatment for pain in the past 3 months; had presented physical or neurological impairment; were sedentary; or were not interested |
| Interventions | **Intervention 1** (n=104): Customised foot orthoses (Grouped intervention Foot orthoses, bracing, taping with sports tape, straps)  **Intervention 2** (n=104): 8mm heel lift Grouped intervenion: heel lifts) |
| Outcomes | **Critical outcomes:**  Self-reported overall pain measured by the VAS at 12 weeks (Medium)  **Important outcomes:**  Pain during algometry as a pseudo-measure of activity pain |
| Notes | **Source of funding:** No funding recieved |

James 2016

| Methods | Factorial randomised comparative effectiveness trial |
| Participants | **Country of study:** Australia  **Sample size**: 124  **Gender:** 52 girls, 72 boys  **Total group age (mean (sd))**: 10.9 (1.48) years  **Apophysitis type:** Calcaneal  **Inclusion criteria:** Children aged between 8 and 14 years, who provided a subjective report of pain located at the calcaneal apophysis (ie, posterior aspect of heel) and with pain on palpation (positive calcaneal squeeze medial and lateral borders)  **Exclusion criteria:** Children who had been diagnosed with a fracture or tumour of the foot/leg, a major lower limb orthopaedic condition and/or a previous diagnosis of infective, reactive or rheumatoid arthritis in the past 12 months |
| Interventions | **Intervention A:** Off the shelf orthoses with cushioning worn in usual footwear or standardised footwear (Group: Foot orthoses, bracing, taping with sports tape, straps)  **Intervention B:** Heel lifts worn in usual footwear or standardised footwear (Group: Heel lifts) |
| Outcomes | **Critical outcomes:**  **-** Self-reported overall pain measured by the Faces Pain Scale at 4 weeks (Short)  - Self-reported physical function measured by the Oxford Ankle foot questionnaire at 4 weeks (Short), 6 months (Medium and 12 months (Long)  - Total adverse events  **Important outcomes:**  - Active range of motion of the relevant lower limb joint |
| Notes | **Source of funding:** Monash Health Emerging Research Grant, Adidas Australia provided the footwear for the trial at no cost to the patient or trial and The Orthotic Laboratory provided the prefabricated orthoses at no cost to the patient or trial.  **Data not reported:** Self-reported physical function measured by the Oxford Ankle foot questionnaire is reported in manuscript in domains, medians and interquartile ranges, raw data obtained from author. |

Kuyucu 2017

| Methods | Randomised controlled trial (parallel group) |
| Participants | **Country of study:** Turkey  **Sample size**: 22  **Sex:** 22 male  **Total group age (mean (sd))**: 13.18 (Not reported) years  **Apophysitis type:** Calcaneal  **Inclusion criteria:** Diagnosed with calcaneal apophysitis with clinical examination and radiography between 2016 and 2017, and all were actively engaged in football playing  **Exclusion criteria:** Radiologically closed calcaneal apophysis, a body mass index (BMI) greater than 25, comorbidities in addition to apophysitis, and a history of heel trauma or conservative treatment |
| Interventions | **Intervention A:** Kinesio taping (Group: Foot orthoses, bracing, taping with sports tape, straps)  **Intervention B:** Hypafix taping without tension (Group: Placebo) |
| Outcomes | **Critical outcomes:**  **-** Self-reported overall pain measured by the VAS at 4 weeks (Short) and 3 months (Medium)  - Self-reported physical function measured by the American Orthopedic Foot-Ankle Society Score at 1 week (Short) and 3 months (Medium) |
| Medium term outcome timepoint | Self reported overall pain - 3 months |
| Notes | **Source of funding:**  This study was unfunded  Noted error in manuscript with means (SD), median, reported as median (SD), median |

Nakase 2020

| Methods | Randomised controlled trial |
| Participants | **Country of study:** Japan  **Sample size**: 49 knees  **Gender:** 1 girl,37 boys  **Total group age (mean (sd))**: 12.4 (1.04) years  **Apophysitis type:** Traction apophysitis of the tibial tubercle  **Inclusion criteria:** Inability to continue physical activities due to anterior knee pain. Onset history could be either progressive or acute after a direct trauma on the anterior tibial tuberosity. Pain had to be localized at the site of the anterior tibial tuberosity site, increased by palpation, and to restrain isometric and eccentric knee extensions  **Exclusion criteria:** Knee effusion, patella instability, patellar tendinopathy at the proximal part and unresolved Osgood Schlatters Disease in adults. |
| Interventions | **Intervention A:** 1% lidocaine 1ml in 20% dextrose (Group: Pharmaceutical intervention)  **Intervention B:** Placebo = 1% lidocaine 1 ml in saline |
| Outcomes | **Critical outcomes:**  Total adverse events  Withdrawals  **Important outcomes:**  - Self‐reported pain experienced during a predefined activity |
| Medium term outcome timepoint | **Important outcomes:**  - Self‐reported pain experienced during a predefined activity |
| Notes | **Source of funding:** No funding declaration |

Perhamre 2011a

| Methods | Randomised controlled trial (cross over) |
| Participants | **Country of study:** Sweden  **Sample size**: 44  **Gender:** 44 boys  **Total group age (mean (sd))**: 11.9 (1.1) years  **Apophysitis type:** Calcaneal  **Inclusion criteria:** Male between 9 and 15 years with a history of calcaneal apohysitis and heel pain present for >2 weeks, but <26 weeks. Engaged in the highest level of sport activity using Engstrom’s activity index  **Exclusion criteria:** Nil described |
| Interventions | **Intervention 1** (n=24): Customised thermoplastic heel cup (Grouped intervention Foot orthoses, bracing, taping with sports tape, straps)  **Intervention 2** (n=20): 5mm heel lift (Grouped intervenion: heel lifts) |
| Outcomes | **Important:**  - Self‐reported pain experienced during a predefined activity measured Borg's CR-10 in two sports (4 weeks) |
| Notes | **Source of funding:** No funding declaration |

Perhamre 2012

| Methods | Randomized controlled trial (Parallel group) |
| Participants | **Country of study:** Sweden  **Sample size**: 50, with 35 participants with calcaneal apophysitis  **Gender:** Unable to be determined from way in which groups were reported but both genders included  **Total group age (median (*****range*****))**: 11 (*9-14*) years  **Apophysitis type:** Calcaneal  **Inclusion criteria:** Both genders, age 9–15 years, diagnosis of Sever’s injury confirmed with tenderness over the lower one- third of the posterior calcaneus and a positive calcaneus compression test on examination by the first author. Heel pain had to be present for more than 2 weeks when examined. The most painful activity had to be a ball sport of some kind (soccer, floor ball, handball, etc.) with a pain level of 4 or more on the Borg CR-10 scale during the last week activities. All participants had to be high-level athletes, measured with Engstrom’s five level activity score index, speak Swedish and consent  **Exclusion criteria:** intermittent pain, specified disease that could interfere with the heel pain, dominant pain from the Achilles tendon, earlier fracture in the area, a poorly defined ache in the lower extremities and participation in another study including any kind of treatment for pain. |
| Interventions | **Intervention A:** Heel cup (Group: Foot orthoses, bracing, taping with sports tape, straps, all with specific information about wear time (e.g. hours per day), materials (e.g. stretch, non‐stretch), and force distribution properties (e.g. density)  **Intervention B:** No treatment |
| Outcomes | **Important:**  **-** Self‐reported pain experienced during a predefined activity (if no overall pain) measured by Borg CR-10 (4 weeks) |
| Notes | **Source of funding:** No funding declaration |

Reesman 2024

| Methods | Randomised controlled trial |
| Participants | **Country of study:** United States of America  **Sample size**: 45  **Gender:** Girls 19, boys 26  **Total group age (mean (sd))**: Not reported  **Apophysitis type:** Tibial traction apophysitis  **Inclusion criteria:**   1. Written approval from the referring physician for potential subject to be considered for enrollment into this study 2. Provision of signed and dated informed consent form 3. Stated willingness to comply with all study procedures and availability for the duration of the study 4. In good general health as evidenced by written approval from referring physician for potential inclusion in study. 5. Has the ability to effectively identify pain/burns and communicate with the investigators or their parents that they are experiencing pain or burning during treatment 6. Referred to CHKD Sports Medicine Physical Therapy by Children's Hospital of The King's Daughters Primary Care Sports Medicine physicians or Children's Hospital of The King's Daughters Orthopedics physicians with a diagnosis of apophysitis of the knee and with a prescription for standard PT treatment with iontophoresis 7. Able and willing to complete iontophoresis treatments within eight (8) weeks of first treatment 8. Must be ambulatory 9. Males 7 to 14 years of age who have not reached skeletal maturity (skeletal maturity based on referring physician's clinical judgement or as demonstrated via radiograph images taken within 90 days of enrollment) 10. Females 7 to 14 years of age who have not reached skeletal maturity (skeletal maturity based on referring physician's clinical judgement or as demonstrated via radiograph images taken within 90 days of enrollment) and who meet one of the following criteria:     1. Pre-menarcheal     2. Within two (2) year post onset of menses 11. Males or females over the age of 14 only with radiographic evidence of skeletal immaturity, with images taken within 90 days of enrollment 12. Index knee symptomatic for pain with activities of daily living or while playing sports.     **Exclusion criteria:**   1. Index knee symptomatic for pain only with palpation and not with activities of daily living or while playing sport 2. Diagnosis of bilateral apophysitis of the knee where both knees meet all of the inclusion criteria 3. Systemic fungal infections 4. Has an implanted electronic device 5. Has a known sensitivity to Dexamethasone 6. Presence of damaged skin, denuded skin, or other recent scar tissue on index knee 7. Presence of active dermatologic conditions in the affected area (e.g., eczema, psoriasis) 8. Presence of an abnormal neurological exam that indicates the subject would have a reduced ability to perceive pain (e.g. peripheral neuropathy) 9. Has a known sensitivity to electrical current 10. Is currently taking systemic steroids 11. Has had iontophoresis with Dexamethasone treatment within the past 30 days 12. Previously enrolled in this study 13. Currently enrolled in another treatment research study |
| Interventions | **Intervention A:** Iontophoresis with Dexamethasone (Group: Pharmaceutical interventions)  **Intervention B:** Iontophoresis with Sodium Chloride (Group: Placebo)  **Intervention C:** Physical therapy (Group: Usual Care - Participants received a standard PT protocol for apophysitis of the knee involving up to 20 visits) |
| Outcomes | **Critical outcomes:**  - Self reported overall pain reported by the FACES Pain rating scale at 4 weeks (Short)  - Self-reported physical function measured by the Lower Extremity Function Scale at 4 weeks and Godin Leisure-Time Activity Scale (Short)  **-** Self-reported participation in sport (Time to meet return to sport criteria) (Short)  - Total adverse events |
| Notes | Study was limited by limited enrollment, primarily as a result of the COVID-19 pandemic. Slow enrollment following the pandemic led to early closure of the study. Results are from the online trial registration.  **Source of funding:** No funding declaration |

Sweeney 2023

| Methods | Randomised controlled trial (parallel group) |
| Participants | **Country of study:** USA  **Sample size**: 32  **Sex:** 29 female, 3 male  **Total group age (mean (sd))**: 10.4 (1.6) years  **Apophysitis type:** Calcaneal  **Inclusion criteria:** Aged 7 to 14 years who participated in a barefoot sport, diagnosed with calcaneal apophysitis  **Exclusion criteria:** History of foot and ankle surgery or rheumatic disease |
| Interventions | **Intervention A** (n=16): Straps  **Intervention B** (n=16): Heel cushioning with specific information |
| Outcomes | **Critical:**  Self-reported physical function (4 weeks)  Total adverse events (4 weeks)    **Important:**  Self‐reported pain experienced during a predefined activity measured (4 weeks) |
| Medium term outcome timepoint | **Critical:**  2. Self-reported physical function (3 months)  5. Total adverse events (3 months)    **Important:**  1. Self‐reported pain experienced during a predefined activity measured (3 months) |
| Notes | **Source of funding:** This study was supported through the American Medical Society for Sports Medicine (AMSSM) Young Investigator’s research grant. Funding provided by the AMSSM Research Committee and AMSSM Foundation. |

Topol 2011

| Methods | Randomized controlled trial |
| Participants | **Country of study:** Argentina  **Sample size**: 65 knees in 54 participants  **Gender:** 3 girls, 51 boys  **Total group age (mean (sd))**: 13.3 (SD not reported)  **Apophysitis type:** Traction apophysitis of the tibial tubercle  **Inclusion criteria:** Girls age 9 to 15 and boys aged 10 to 17 in the area of Rosario, Argentina, were screened for anterior knee pain, but only if they were involved in a jumping or kicking sport on an organized team with a coach. Absence of either patellofemoral crepitus or patellar origin tenderness was required, as well as reproduction of the exact pain and localization of pain precisely to the tibial tuberosity during a single leg squat. Once confident of the diagnosis, patients were required to have attempted at least 2 months of formal and gently progressive hamstring stretching, quads strengthening, gradual sport reintroduction, and to have had pain with sport for at least 3 months.  **Exclusion criteria:** Not reported |
| Interventions | **Intervention A:** Pharmaceutical intervention (lidocaine 1% and 12.5% dextrose)  **Intervention B:** Placebo pharmaceutical internvention (lidocaine 1%)  **Intervention C:** Usual Care including: Physical therapist instructed stretching and exercise methods with supplementary video with single follow up session |
| Outcomes | No short term outcomes |
| Medium term outcome timepoint | **Important:**  Self‐reported pain experienced during a predefined activity at 3 months (Medium) |
| Notes | **Source of funding:** No funding declaration |

Wiegerinck 2016

| Methods | Randomised controlled trial (parallel group) |
| Participants | **Country of study:** Netherlands  **Sample size**: 98  **Gender:** 76 male  **Total group age (mean (sd))**: 10.6 (1.6) years  **Apophysitis type:** Calcaneal  **Inclusion criteria:** Diagnosis of calcaneal apophysitis, based on clinical symptoms with: Positive squeeze test (pressure pain at posterolateral and/or medial side of heel. No abnormalities on basic foot/ankle radiography. Pain complaints for at least 4 wk before the start of treatment FPS-R should be at least 30 mm at time of inclusion Capable of performing prescribed exercises.  **Exclusion criteria:** Age< 8 > 15 y Subject previously underwent one of the evaluated treatment modalities for current complaints Other injury to the affected foot or leg over the last year. Complaints based on other foot or ankle pathology |
| Interventions | **Intervention A:** Exercise program from physical therapist (Group: Exercise)  **Intervention B:** Heel lift - ViscoHeel (Group: Heel lifts)  **Intervention C:** No treatment and were advised pragmatically to cease pain inducing activities |
| Outcomes | **Critical:**  Self‐reported physical function (6 weeks)    **Important:**  Participant‐reported treatment success (6 weeks)  Self‐reported pain experienced during a predefined activity (6 weeks) |
| Medium term outcome timepoint | **Critical:**  2. Self‐reported physical function (3 months)  **Important:**  1. Participant‐reported treatment success (3 months)  2. Self‐reported pain experienced during a predefined activity (3 months) |
| Notes | No funding disclosed |

## Footnotes

VAS - Visual Analogue Scale

Borg CR-10 - Borg CR-10 (Category-Ratio 10)

FPS-R - Faces Pain Scale-Revised (FPS-R)

## References to studies

### Alfaro-Santafa 2021 {published data only}

- \*Alfaro-Santafé J, Gómez-Bernal A, Lanuza-Cerzócimo C, Alfaro-Santafé J, Pérez-Morcillo A, Almenar-Arasanz A. Effectiveness of custom-made foot orthoses vs. heel-lifts in children with calcaneal apophysitis (Sever’s Disease): a CONSORT-compliant randomized trial. Children 2021;8(11):1-10.
- NCT03960086. Effectiveness of custom-made foot orthoses vs heel lifts in children with calcaneal apophysitis. https://clinicaltrials.gov/study/NCT03960086 (first posted 22 May 2019).

### James 2016 {published data only}

- \*James AM, Williams CM, Haines TP. Effectiveness of footwear and foot orthoses for calcaneal apophysitis: a 12-month factorial randomised trial. British Journal of Sports Medicine 2016;50(20):1268-75.

### Kuyucu 2017 {published data only}

- \*Kuyucu E, Gulenc B, Bicer H, Erdil M. Assessment of the kinesiotherapy's efficacy in male athletes with calcaneal apophysitis. Journal of Orthopaedic Surgery and Research 2017;12(1):146.

### Nakase 2020 {published data only}

- \*Nakase J, Oshima T, Takata Y, Shimozaki K, Asai K, Tsuchiya H. No superiority of dextrose injections over placebo injections for Osgood-Schlatter disease: a prospective randomized double-blind study. Archives of Orthopaedic and Trauma Surgery 2020;140(2):197-202. [DOI: 10.1007/s00402-019-03297-2]

### Perhamre 2011a {published data only}

- \*Perhamre S, Janson, S, Norlin R, Klassbo M. Sever's injury; treat it with a heel cup: a randomized, crossover study with two insole alternatives. Scandinavian Journal of Medicine & Science in Sports 2011;21(6):e42-e47.

### Perhamre 2012 {published data only}

- \*Perhamre S, Lundin F, Klassbo M, Norlin R. A heel cup improves the function of the heel pad in Sever's injury: effects on heel pad thickness, peak pressure and pain. Scandinavian Journal of Medicine & Science in Sports 2012;22(4):516-22. [DOI: 10.1111/j.1600-0838.2010.01266.x]

### Reesman 2024 {unpublished data only}

- \*NCT03606980. Iontophoresis with dexamethasone and physical therapy to treat apophysitis of the knee in pediatrics. https://clinicaltrials.gov/study/NCT03606980 (first posted 31 July 2018).

### Sweeney 2023 {published data only}

- \*Sweeney EA, Little CC, Wilson JC, Potter MN, Seehusen CN, Howell DR. Comparison of braces for treatment of Sever's disease (calcaneal apophysitis) in barefoot athletes: a randomized clinical trial. Journal of Athletic Training 2023;58(5):437-44.
- Sweeney E, Little C, Wilson J, Potter M, Seehusen C, Howell D. Comparison of braces for treatment of Sever's disease in barefoot athletes. Clinical Journal of Sport Medicine 2022;32(2):164. [DOI: 10.1097/JSM.0000000000001016]

### Topol 2011 {published data only}

- \*Topol GA, Podesta LA, Reeves KD, Raya MF, Fullerton BD, Yeh HW. Hyperosmolar dextrose injection for recalcitrant Osgood-Schlatter disease. Pediatrics 2011;128(5):e1121-8. [DOI: 10.1542/peds.2010-19310]

### Wiegerinck 2016 {published data only}

- \*Wiegerinck JI, Zwiers R, Sierevelt IN, Van Weert HC, Van Dijk CN, Struijs PA. Treatment of calcaneal apophysitis: wait and see versus orthotic device versus physical therapy: a pragmatic therapeutic randomized clinical trial. Journal of Pediatric Orthopedics 2016;36(2):152‐7.
