# Supplementary material for: Non‐surgical treatment for lower limb apophyseal injuries
Source: Cochrane Database Syst Rev. 2026 Jul 15;2026(7):CD015156. doi: 10.1002/14651858.CD015156.pub2 (PMC13370774; doi:10.1002/14651858.CD015156.pub2)
Supplement: Supplementary file 3 — Supplementary material 3 Characteristics of excluded studies [file CD015156-SUP-03-characteristicsOfExcludedStudies.html]

Characteristics of excluded studies


# Supplementary material 3 to: Non-surgical treatment for lower limb apophyseal injuries

Williams CM, Krommes K, Paterson KL, Haines T, Caserta A, Thorborg K
  
https://doi.org/10.1002/14651858.CD015156.pub2

The material in this section has been supplied by the author(s) for publication under a Licence for Publication and the author(s) are solely responsible for the material. Cochrane has reviewed this material, but Cochrane has not copyedited, formatted or proofread. Cochrane accordingly gives no representations or warranties of any kind in relation to, and accepts no liability for any reliance on or use of, such material.

Back to top

# Characteristics of excluded studies

## Table of contents

- Studies ordered by Study ID
  - Blumenfeld 2023
  - Feyzioğlu 2018
  - Gaulrapp 2016
  - Gerulis 2004
  - Hadell 2015
  - Holden 2021
  - Karaczun 2016
  - Lucciani 2016
  - Molund 2018
  - Perhamre 2011 (b)
  - Rathleff 2020
  - Rathleff 2023
  - Selhorst 2023
  - Shields 2016
  - Smith 1977
  - Universitair Medisch Centrum Groningen 2025
  - University of Delaware 2021
  - Vermass 2018
  - Wu 2022
- References to studies

## Studies ordered by Study ID

| Study | Reason for exclusion |
| --- | --- |
| Blumenfeld 2023 | Ineligible study design - Secondary data analysis of trial including adolescents with Patellofemoral pain and/or Osgood-Schlatter Disease |
| Feyzioğlu 2018 | Ineligible study design - protocol for case series design |
| Gaulrapp 2016 | Ineligible study design - case series design |
| Gerulis 2004 | Ineligible study design - cohort study with non-randomised interventions and unclear processes for allocating treatments |
| Hadell 2015 | Ineligible participant population - due to not the condition of interest |
| Holden 2021 | Ineligible study design - cohort study |
| Karaczun 2016 | Unable to source data - abandoned trial registration |
| Lucciani 2016 | Unable to source data - no response from authors for requests of data |
| Molund 2018 | Ineligible participant population - due to age and condition studied |
| Perhamre 2011 (b) | Ineligible study design - cohort study |
| Rathleff 2020 | Ineligible study design - cohort study |
| Rathleff 2023 | Ineligible study design - prognostic study |
| Selhorst 2023 | Ineligible participant population - recruitment focused on primary musculoskeletal pain |
| Shields 2016 | Ineligible study design - commentary |
| Smith 1977 | Ineligible study design - commentary |
| Universitair Medisch Centrum Groningen 2025 | Unable to source data - abandoned trial |
| University of Delaware 2021 | Ineligible study design - registration of a cohort study |
| Vermass 2018 | Ineligible study design - prognostic study |
| Wu 2022 | Ineligible participant population - condition inadequately described in adults over the age of likelihood of condition being present |

## References to studies

### Blumenfeld 2023 {published data only}

- Blumenfeld MB, Straszek CLN, Holden S, Thorborg K, Rathleff MS. Does an activity modification strategy for adolescents with patellofemoral pain and Osgood-Schlatter affect sedentary time? An ancillary analysis. BMJ Open Sport and Exercise Medicine 2023;9(Supplement 1):A9-A10. [DOI: https://dx.doi.org/10.1136/bmjsem-2023-sportskongres2023.26]

### Feyzioğlu 2018 {published data only}

- Feyzioğlu Ö, Öztürk Ö, Muğrabi S. The effects of custom-made insoles on foot pressure redistribution and spatio - temporal gait parameters in Sever disease. ClinicalTrials.Gov 2018. [CLINICALTRIALS.GOV: NCT03787316]

### Gaulrapp 2016 {published data only}

- Gaulrapp H. Clinical examination, imaging and therapy of Osgood-Schlatter's disease. Der Orthopade 2016;45(3):219-225. [DOI: https://dx.doi.org/10.1007/s00132-016-3225-1]

### Gerulis 2004 {published data only}

- Gerulis V, Kalesinskas R, Pranckevicius S, Birgeris P. Importance of conservative treatment and physical load restriction to the course of Osgood-Schlatter's disease. Medicina (Kaunas, Lithuania) 2004;40(4):363-369.

### Hadell 2015 {published data only}

- Fadell, M. Intra Articular injections with platelet rich plasma in patients with Juvenile Osteochondritis Dissecans of the knee. ClinicalTrials.Gov 2015. [CLINICALTRIALS.GOV: NCT02397278]

### Holden 2021 {published data only}

- Holden S, Olesen, JL, Winiarski LM, Krommes K, Thorborg K, Hölmich P, et al. Is the prognosis of Osgood-Schlatter poorer than anticipated? A prospective cohort study with 24-Month follow-up. Orthopaedic journal of sports medicine 2021;9 (8):9. [DOI: 10.1177/23259671211022239]

### Karaczun 2016 {published data only}

- Karaczun M. Comparison of methods for the treatment of Osgood-Schlatter disease. UMIN-CTR Clinical Trial 2016. [UMIN-CTR CLINICAL TRIAL: UMIN000013899]

### Lucciani 2016 {published data only (unpublished sought but not used)}

- \*Lucciani JF. Treatment of the Osgood Schlatter Disease by immobilization ( Ankle Cruro Resin ) versus sporting rest ( reference treatment ) : randomized controlled study. https://clinicaltrials.gov/study/NCT02824172 2016.

### Molund 2018 {published data only}

- Molund M, Husebye EE, Hellesnes J, Nilsen F, Hvaal K. Proximal medial gastrocnemius recession and stretching versus stretching as treatment of chronic plantar heel pain. Foot & ankle International 2018;39(12):1423-1431. [DOI: https://dx.doi.org/10.1177/1071100718794659]

### Perhamre 2011 (b) {published data only}

- Perhamre S, Janson S, Norlin R, Klassbo M. Sever's injury: treatment with insoles provides effective pain relief. Scandinavian journal of medicine & science in sports 2011;21(6):819‐823. [DOI: 10.1111/j.1600-0838.2010.01051.x]

### Rathleff 2020 {published data only}

- \*Rathleff MS, Winiarski L, Krommes K, Graven-Nielsen T, Hölmich P, Olesen J, etal. Activity modification and knee strengthening for Osgood-Schlatter disease: a prospective cohort study. Orthopaedic journal of sports medicine 2020;8(4):2325967120911106. [DOI: https://doi.org/10.1177/23259671209111]

### Rathleff 2023 {published data only}

[ctg: NCT05791513]

- Rathleff MS. The effectiveness of using a clinical support tool in managing adolescents with non-traumatic knee pain. ClinicalTrials.gov 2023. [CLINICALTRIALS.GOV: NCT05791513]

### Selhorst 2023 {published data only}

- Selhorst M. Psychologically informed education intervention for adolescents with atraumatic lower-extremity injuries. ClinicalTrials.gov 2023. [CLINICALTRIALS.GOV: NCT05701618]

### Shields 2016 {published data only}

- Shields N. Wait and see, heel raise and eccentric exercise may be equally effective treatments for children with calcaneal apophysitis. Journal of Physiotherapy 2016;62(2):112-112. [DOI: 10.1016/j.jphys.2015.12.004]

### Smith 1977 {published data only}

- Smith JB. Knee problems in children. Pediatric Clinics of North America 1977;24(4):841-855. [DOI: http://dx.doi.org/10.1016/S0031-3955(16)33502-7]

### Universitair Medisch Centrum Groningen 2025 {published data only (unpublished sought but not used)}

- Universitair Medisch Centrum Groningen. The use of a patellar strap in children with Osgood-Schlatter disease: a pilot study of the short term effect on symptoms and sports participation. https://onderzoekmetmensen.nl/en/trial/42555.

### University of Delaware 2021 {published data only}

[ctg: NCT04816188]

- University of Delaware. Heel pain in adolescents: a pilot study on the effectiveness of exercise therapy and activity modification. ClinicalTrials.gov 2021. [CLINICALTRIALS.GOV: NCT04816188]

### Vermass 2018 {published data only}

- Vermaas C. Heel complaints in young athletes. Overview of Medical Research in the Netherlands (OMON), formerly the Dutch Trial register 2018. [NTR: NTR7189]

### Wu 2022 {published data only}

- Wu Z, Tu X, Tu Z. Hyperosmolar dextrose injection for Osgood-Schlatter disease: a double-blind, randomized controlled trial. Archives of orthopaedic and trauma surgery 2022;142(9):2279-2285. [DOI: https://dx.doi.org/10.1007/s00402-021-04223-1]
