# Supplementary material for: Non‐surgical treatment for lower limb apophyseal injuries
Source: Cochrane Database Syst Rev. 2026 Jul 15;2026(7):CD015156. doi: 10.1002/14651858.CD015156.pub2 (PMC13370774; doi:10.1002/14651858.CD015156.pub2)
Supplement: Supplementary file 4 — Supplementary material 4 Characteristics of ongoing studies [file CD015156-SUP-04-characteristicsOfOngoingStudies.html]

Characteristics of ongoing studies


# Supplementary material 4 to: Non-surgical treatment for lower limb apophyseal injuries

Williams CM, Krommes K, Paterson KL, Haines T, Caserta A, Thorborg K
  
https://doi.org/10.1002/14651858.CD015156.pub2

The material in this section has been supplied by the author(s) for publication under a Licence for Publication and the author(s) are solely responsible for the material. Cochrane has reviewed this material, but Cochrane has not copyedited, formatted or proofread. Cochrane accordingly gives no representations or warranties of any kind in relation to, and accepts no liability for any reliance on or use of, such material.

Back to top

# Characteristics of ongoing studies

## Table of contents

- Studies ordered by Study ID
  - Carl 2025
  - Faude 2020
  - Krommes 2025
- References to studies

## Studies ordered by Study ID

Carl 2025

| Methods | Randomised Controlled Trial (Parallel) |
| Participants | **Country of study:** USA  **Sample size**: Unknown  **Gender:** Unknown  **Total group age (mean (sd))**: Unknown  **Apophysitis type:** Calcaneal (Severs), Traction apophysitis of the tibial tubercle (Osgood‐Schlatter's disease)  **Inclusion criteria:** Children aged between 8 and 17 years, diagnosis of OSS, SLJ, or Sever's disease, must have regular access to the Internet  **Exclusion criteria:** history of prior treatment for OSS, SLJ, or Sever's disease, history of previous injury to the affected joint requiring more than 1 week off of sports or activities |
| Interventions | **Intervention 1:** Sypmtomative treatment (Control)  **Intervention 2:** Static stretch (Group - Stretching exercises (guided either by a health professional within the clinical setting or implemented within the home environment)  **Intervention 3:** Active Elongation (Group - Exercise (balance, alignment/postural, strength, stability specified) with specific dosage information ) |
| Outcomes | **Major outcome:**  Self‐reported overall pain  Self‐reported participation in sports |
| Starting date | 10/2012 |
| Notes | Completion date 10/2023 |

Faude 2020

| Methods | Randomised controlled trial (parallel) |
| Participants | **Country of study:** Switzerland  **Sample size**: Not reported  **Total group age (mean (sd))**: Not reported  **Apophysitis type:** Traction apophysitis of the tibial tubercle (Osgood‐Schlatter's disease)  **Inclusion criteria:** Uni- or bilateral OSD, ability to follow instructions, sufficient knowledge of German, availability: can participate in two exercise sessions per week for a period of 8 weeks  **Exclusion criteria:** any history of knee surgery, medication intake affecting the knee, unstable fractures, neurological disorders, systematic diseases, already in physiotherapeutic treatment because of the knee, not possible to do any physiotherapy sessions at the Universitäts-Kinderspital Basel (UKBB) |
| Interventions | Intervention 1: Physiotherapy program (Groups - Manual therapy such as dry needling or massage with specific dosage information and Exercise)  Intervention 2: Usual care |
| Outcomes | **Major outcomes:**  - Self-reported physical function  - Self-reported overall pain measured by visual analogue scale  - Self-reported participation in sports  **Minor outcomes:**  - Active range of motion of the relevant lower limb joint |
| Starting date | 11/11/2020 |
| Notes | Completion date 07/07/2022 |

Krommes 2025

| Study name | Comparison of Two Different Treatment Approaches for Adolescents With Osgood Schlatter (SOGOOD) |
| Methods | Randomised controlled trial (Parallel) |
| Participants | **Country of study:** Denmark  **Sample size**: N/A  **Gender:** N/A  **Total group age (mean (sd))**:  **Apophysitis type:** Traction apophysitis of the tibial tubercle (Osgood‐Schlatter's disease)  **Inclusion criteria:** Tenderness on palpation of the tibial tuberosity or pain during resisted isometric knee extensions, Insidious onset of pain or swelling of the tibial tuberosity for ≥6 weeks, provoked by at least 2 of the following positions or activities; prolonged sitting or kneeling, squatting, running, hopping/jumping, stair walking or during multidirectional sports, clinical diagnosis of Osgood Schlatter, markedly reduced sports participation OR severely affected by pain during sports participation  **Exclusion criteria:** Other primary pathology or complaints from other structures of the knee, other injuries, complaints or illnesses that may cause disability, or specifically restricts levels of physical activity or sports participation, previous surgery in the lower extremities or lumbar spine, congenital deformities, device implants og cysts og tumors of the knee, participants not willing to cease concomitant treatment, participants and their parents not able to understand and communicate in written and verbal Danish |
| Interventions | **Intervention A -** Active novel self management of load and progressive excercise (Group - Exercise (balance, alignment/postural, strength, stability specified) with specific dosage information )    **Intervention B** - Usual Care |
| Outcomes | **Major outcomes**  - Self-reported physical function (Short, Medium and long term)  - Participant-reported treatment success (Medium term)  - Self-reported participation in sports (Medium term)  - Total adverse events    **Minor outcomes**  **-** Self-reported pain experienced during a predefined activity (Medium term) |
| Starting date | 01/01/2022 |
| Notes | Completion date: 08/03/2024 |

## References to studies

### Carl 2025 {published data only}

- Carl R. Comparison of Three Treatments for Lower Extremity Apophysitis. https://clinicaltrials.gov/study/NCT01826071 10th February, 2025.

### Faude 2020 {published data only (unpublished sought but not used)}

- \*Faude, O. Treatment for Osgood Schlatter Patients With a Physiotherapy Program (TrOPhy). https://classic.clinicaltrials.gov/show/NCT04716608 first received 11 November 2020.

### Krommes 2025 {unpublished data only}

- \*Krommes, K. A Comparison of Two Different Treatment Approaches for Adolescents With Osgood Schlatter (SOGOOD). https://clinicaltrials.gov/study/NCT05174182.
