# Supplementary material for: Non‐surgical treatment for lower limb apophyseal injuries
Source: Cochrane Database Syst Rev. 2026 Jul 15;2026(7):CD015156. doi: 10.1002/14651858.CD015156.pub2 (PMC13370774; doi:10.1002/14651858.CD015156.pub2)
Supplement: Supplementary file 5 — Supplementary material 5 Risk of bias [file CD015156-SUP-05-riskOfBias2.html]

Risk of bias


# Supplementary material 5 to: Non-surgical treatment for lower limb apophyseal injuries

Williams CM, Krommes K, Paterson KL, Haines T, Caserta A, Thorborg K
  
https://doi.org/10.1002/14651858.CD015156.pub2

The material in this section has been supplied by the author(s) for publication under a Licence for Publication and the author(s) are solely responsible for the material. Cochrane has reviewed this material, but Cochrane has not copyedited, formatted or proofread. Cochrane accordingly gives no representations or warranties of any kind in relation to, and accepts no liability for any reliance on or use of, such material.

Back to top

# Risk of bias

## Table of contents

- Risk of bias for analysis 1.1 Overall pain in the short term
- Risk of bias for analysis 1.2 Physical function in the short term
- Risk of bias for analysis 1.3 Participation in sport in the short term
- Risk of bias for analysis 1.4 Adverse events in the short term
- Risk of bias for analysis 1.5 Pain during an activity in the medium term
- Risk of bias for analysis 1.6 Pain during an activity in the long term
- Risk of bias for analysis 2.1 Overall pain in the short term
- Risk of bias for analysis 2.2 Physical function in the short term
- Risk of bias for analysis 2.3 Participation in sport in the short term
- Risk of bias for analysis 2.4 Adverse events in the short term
- Risk of bias for analysis 2.5 Pain during an activity in the medium term
- Risk of bias for analysis 2.6 Pain during an activity in the long term
- Risk of bias for analysis 3.1 Overall pain in the short term
- Risk of bias for analysis 3.2 Overall pain in the medium term
- Risk of bias for analysis 3.3 Physical function in the short term
- Risk of bias for analysis 3.4 Physical function in the medium term
- Risk of bias for analysis 4.1 Overall pain in the short term
- Risk of bias for analysis 4.2 Overall pain in the medium term
- Risk of bias for analysis 4.3 Physical function in the short term
- Risk of bias for analysis 4.4 Physical function in the medium term
- Risk of bias for analysis 4.5 Physical function in the long term
- Risk of bias for analysis 4.6 Adverse events
- Risk of bias for analysis 4.8 Joint range of motion in the short term
- Risk of bias for analysis 5.1 Physical function in the short term
- Risk of bias for analysis 5.3 Adverse events
- Risk of bias for analysis 5.4 Pain during activity in the short term
- Risk of bias for analysis 5.5 Pain during activity in the medium term

## Risk of bias

Risk of bias for analysis 1.1 Overall pain in the short term

| **Study** | **Bias** | | | | | | | | | | | |
| **Randomisation process** | | **Deviations from intended interventions** | | **Missing outcome data** | | **Measurement of the outcome** | | **Selection of the reported results** | | **Overall** | |
| Authors' judgement | Support for judgement | Authors' judgement | Support for judgement | Authors' judgement | Support for judgement | Authors' judgement | Support for judgement | Authors' judgement | Support for judgement | Authors' judgement | Support for judgement |
| Reesman 2024 | Some concerns | No information about randomisation process. | High risk of bias | No information about deviations due to trial ending early | High risk of bias | Trial ended early, missing outcome data. | High risk of bias | Trial ended early, limited information about how outcomes were collected and the outcome is subjective | Some concerns | No protocol published. | High risk of bias | No other bias identifed |

Risk of bias for analysis 1.2 Physical function in the short term

| **Study** | **Bias** | | | | | | | | | | | |
| **Randomisation process** | | **Deviations from intended interventions** | | **Missing outcome data** | | **Measurement of the outcome** | | **Selection of the reported results** | | **Overall** | |
| Authors' judgement | Support for judgement | Authors' judgement | Support for judgement | Authors' judgement | Support for judgement | Authors' judgement | Support for judgement | Authors' judgement | Support for judgement | Authors' judgement | Support for judgement |
| Reesman 2024 | Some concerns | No information about randomisation process. | High risk of bias | No information about deviations due to trial ending early | High risk of bias | Trial ended early, missing outcome data. | High risk of bias | Trial ended early, limited information about how outcomes were collected and the outcome is subjective | Some concerns | No protocol published. | High risk of bias | No protocol published. |

Risk of bias for analysis 1.3 Participation in sport in the short term

| **Study** | **Bias** | | | | | | | | | | | |
| **Randomisation process** | | **Deviations from intended interventions** | | **Missing outcome data** | | **Measurement of the outcome** | | **Selection of the reported results** | | **Overall** | |
| Authors' judgement | Support for judgement | Authors' judgement | Support for judgement | Authors' judgement | Support for judgement | Authors' judgement | Support for judgement | Authors' judgement | Support for judgement | Authors' judgement | Support for judgement |
| Reesman 2024 | Some concerns | No information about randomisation process. | High risk of bias | No information about deviations due to trial ending early | High risk of bias | Trial ended early, missing outcome data. | High risk of bias | Trial ended early, limited information about how outcomes were collected and the outcome is subjective | Some concerns | No protocol published. | High risk of bias | No other bias identified |

Risk of bias for analysis 1.4 Adverse events in the short term

| **Study** | **Bias** | | | | | | | | | | | |
| **Randomisation process** | | **Deviations from intended interventions** | | **Missing outcome data** | | **Measurement of the outcome** | | **Selection of the reported results** | | **Overall** | |
| Authors' judgement | Support for judgement | Authors' judgement | Support for judgement | Authors' judgement | Support for judgement | Authors' judgement | Support for judgement | Authors' judgement | Support for judgement | Authors' judgement | Support for judgement |
| Nakase 2020 | Some concerns | Randomisation process was not described. | Low risk of bias | Participants and people delivering intervention were blinded. No differences between groups. | Low risk of bias | Data available for nearly all participants. | Some concerns | It was unclear if the outcome assessors were aware of the intervention group. | Some concerns | No information on pre-specified analysis plan. | Some concerns | No other bias identified. |
| Reesman 2024 | Some concerns | No information about randomisation process. | High risk of bias | No information about deviations due to trial ending early | High risk of bias | Trial ended early, missing outcome data. | Some concerns | Trial ended early, limited information about how outcomes were collected | Some concerns | No protocol published. | High risk of bias | No other bias identified |

Risk of bias for analysis 1.5 Pain during an activity in the medium term

| **Study** | **Bias** | | | | | | | | | | | |
| **Randomisation process** | | **Deviations from intended interventions** | | **Missing outcome data** | | **Measurement of the outcome** | | **Selection of the reported results** | | **Overall** | |
| Authors' judgement | Support for judgement | Authors' judgement | Support for judgement | Authors' judgement | Support for judgement | Authors' judgement | Support for judgement | Authors' judgement | Support for judgement | Authors' judgement | Support for judgement |
| Nakase 2020 | Some concerns | Randomisation process was not described. | Low risk of bias | Participants and people delivering intervention were blinded. No differences between groups. | Low risk of bias | Data available for nearly all participants. | Some concerns | It was unclear if the method of measuring outcome was appropriate for the knee as only the VISA was described. It was unclear if the outcome assessors were aware of the intervention group. | Some concerns | No information on pre-specified analysis plan. | Some concerns | No other bias identified. |
| Topol 2011 | Low risk of bias | Random numbers table was used for assignment to supervised usual care or to an injection solution group blinded to the subject, guardian, and the treating/evaluating physician. | Some concerns | Those in the usual care group were aware of not receiving either injection, people delivering this arm of the trial were also aware of assignment. | Low risk of bias | All participant data included. | Low risk of bias | Outcome measurement described | Some concerns | Protocol analysis plan not available. | Some concerns | No other bias identified. |

Risk of bias for analysis 1.6 Pain during an activity in the long term

| **Study** | **Bias** | | | | | | | | | | | |
| **Randomisation process** | | **Deviations from intended interventions** | | **Missing outcome data** | | **Measurement of the outcome** | | **Selection of the reported results** | | **Overall** | |
| Authors' judgement | Support for judgement | Authors' judgement | Support for judgement | Authors' judgement | Support for judgement | Authors' judgement | Support for judgement | Authors' judgement | Support for judgement | Authors' judgement | Support for judgement |
| Topol 2011 | Low risk of bias | Random numbers table was used for assignment to supervised usual care or to an injection solution group blinded to the subject, guardian, and the treating/evaluating physician. | Some concerns | Those in the usual care group were aware of not receiving either injection, people delivering this arm of the trial were also aware of assignment. | Low risk of bias | All participant data included. | Low risk of bias | Outcome measurement described | Some concerns | Protocol analysis plan not available. | Some concerns | No other bias identified. |

Risk of bias for analysis 2.1 Overall pain in the short term

| **Study** | **Bias** | | | | | | | | | | | |
| **Randomisation process** | | **Deviations from intended interventions** | | **Missing outcome data** | | **Measurement of the outcome** | | **Selection of the reported results** | | **Overall** | |
| Authors' judgement | Support for judgement | Authors' judgement | Support for judgement | Authors' judgement | Support for judgement | Authors' judgement | Support for judgement | Authors' judgement | Support for judgement | Authors' judgement | Support for judgement |
| Reesman 2024 | Some concerns | No information about randomisation process. | High risk of bias | No information about deviations due to trial ending early | High risk of bias | Trial ended early, missing outcome data. | High risk of bias | Trial ended early, limited information about how outcomes were collected and the outcome is subjective | Some concerns | No protocol published. | High risk of bias | No other bias identifed |

Risk of bias for analysis 2.2 Physical function in the short term

| **Study** | **Bias** | | | | | | | | | | | |
| **Randomisation process** | | **Deviations from intended interventions** | | **Missing outcome data** | | **Measurement of the outcome** | | **Selection of the reported results** | | **Overall** | |
| Authors' judgement | Support for judgement | Authors' judgement | Support for judgement | Authors' judgement | Support for judgement | Authors' judgement | Support for judgement | Authors' judgement | Support for judgement | Authors' judgement | Support for judgement |
| Reesman 2024 | Some concerns | No information about randomisation process. | High risk of bias | No information about deviations due to trial ending early | High risk of bias | Trial ended early, missing outcome data. | High risk of bias | Trial ended early, limited information about how outcomes were collected and the outcome is subjective | Some concerns | No protocol published. | High risk of bias | No protocol published. |

Risk of bias for analysis 2.3 Participation in sport in the short term

| **Study** | **Bias** | | | | | | | | | | | |
| **Randomisation process** | | **Deviations from intended interventions** | | **Missing outcome data** | | **Measurement of the outcome** | | **Selection of the reported results** | | **Overall** | |
| Authors' judgement | Support for judgement | Authors' judgement | Support for judgement | Authors' judgement | Support for judgement | Authors' judgement | Support for judgement | Authors' judgement | Support for judgement | Authors' judgement | Support for judgement |
| Reesman 2024 | Some concerns | No information about randomisation process. | High risk of bias | No information about deviations due to trial ending early | High risk of bias | Trial ended early, missing outcome data. | High risk of bias | Trial ended early, limited information about how outcomes were collected and the outcome is subjective | Some concerns | No protocol published. | High risk of bias | No other bias identified |

Risk of bias for analysis 2.4 Adverse events in the short term

| **Study** | **Bias** | | | | | | | | | | | |
| **Randomisation process** | | **Deviations from intended interventions** | | **Missing outcome data** | | **Measurement of the outcome** | | **Selection of the reported results** | | **Overall** | |
| Authors' judgement | Support for judgement | Authors' judgement | Support for judgement | Authors' judgement | Support for judgement | Authors' judgement | Support for judgement | Authors' judgement | Support for judgement | Authors' judgement | Support for judgement |
| Reesman 2024 | Some concerns | No information about randomisation process. | High risk of bias | No information about deviations due to trial ending early | High risk of bias | Trial ended early, missing outcome data. | Some concerns | Trial ended early, limited information about how outcomes were collected | Some concerns | No protocol published. | High risk of bias | No other bias identified |

Risk of bias for analysis 2.5 Pain during an activity in the medium term

| **Study** | **Bias** | | | | | | | | | | | |
| **Randomisation process** | | **Deviations from intended interventions** | | **Missing outcome data** | | **Measurement of the outcome** | | **Selection of the reported results** | | **Overall** | |
| Authors' judgement | Support for judgement | Authors' judgement | Support for judgement | Authors' judgement | Support for judgement | Authors' judgement | Support for judgement | Authors' judgement | Support for judgement | Authors' judgement | Support for judgement |
| Topol 2011 | Low risk of bias | Random numbers table was used for assignment to supervised usual care or to an injection solution group blinded to the subject, guardian, and the treating/evaluating physician. | Some concerns | Those in the usual care group were aware of not receiving either injection, people delivering this arm of the trial were also aware of assignment. | Low risk of bias | All participant data included. | Low risk of bias | Outcome measurement described | Some concerns | Protocol analysis plan not available. | Some concerns | No other bias identified. |

Risk of bias for analysis 2.6 Pain during an activity in the long term

| **Study** | **Bias** | | | | | | | | | | | |
| **Randomisation process** | | **Deviations from intended interventions** | | **Missing outcome data** | | **Measurement of the outcome** | | **Selection of the reported results** | | **Overall** | |
| Authors' judgement | Support for judgement | Authors' judgement | Support for judgement | Authors' judgement | Support for judgement | Authors' judgement | Support for judgement | Authors' judgement | Support for judgement | Authors' judgement | Support for judgement |
| Topol 2011 | Low risk of bias | Random numbers table was used for assignment to supervised usual care or to an injection solution group blinded to the subject, guardian, and the treating/evaluating physician. | Some concerns | Those in the usual care group were aware of not receiving either injection, people delivering this arm of the trial were also aware of assignment. | Low risk of bias | All participant data included. | Low risk of bias | Outcome measurement described | Some concerns | Protocol analysis plan not available. | Some concerns | No other bias identified. |

Risk of bias for analysis 3.1 Overall pain in the short term

| **Study** | **Bias** | | | | | | | | | | | |
| **Randomisation process** | | **Deviations from intended interventions** | | **Missing outcome data** | | **Measurement of the outcome** | | **Selection of the reported results** | | **Overall** | |
| Authors' judgement | Support for judgement | Authors' judgement | Support for judgement | Authors' judgement | Support for judgement | Authors' judgement | Support for judgement | Authors' judgement | Support for judgement | Authors' judgement | Support for judgement |
| Kuyucu 2017 | Some concerns | No information about randomisation or baseline differences between groups. | High risk of bias | Unclear if participants knew about their group allocation, no information to understand the effect of assignment, important because of sex based differences in apophysis closure. No information about if there was a deviation from usual practice. The analysis relied on multiple unadjusted statistical tests (including within-group comparisons) without clearly estimating between-group effects | Low risk of bias | Nearly all participant data were reported. | Some concerns | Limited information about the anchor for subjective VAS data. | Some concerns | No pre-specified analysis plan identified. | High risk of bias | No other bias identified |

Risk of bias for analysis 3.2 Overall pain in the medium term

| **Study** | **Bias** | | | | | | | | | | | |
| **Randomisation process** | | **Deviations from intended interventions** | | **Missing outcome data** | | **Measurement of the outcome** | | **Selection of the reported results** | | **Overall** | |
| Authors' judgement | Support for judgement | Authors' judgement | Support for judgement | Authors' judgement | Support for judgement | Authors' judgement | Support for judgement | Authors' judgement | Support for judgement | Authors' judgement | Support for judgement |
| Kuyucu 2017 | Some concerns | No information about randomisation or baseline differences between groups. | High risk of bias | Unclear if participants knew about their group allocation, no information to understand the effect of assignment, important because of sex based differences in apophysis closure. No information about if there was a deviation from usual practice. The analysis relied on multiple unadjusted statistical tests (including within-group comparisons) without clearly estimating between-group effects | Low risk of bias | Nearly all participant data were reported. | Some concerns | Limited information about the anchor for subjective VAS data. | Some concerns | No pre-specified analysis plan identified. | High risk of bias | No other bias identified |

Risk of bias for analysis 3.3 Physical function in the short term

| **Study** | **Bias** | | | | | | | | | | | |
| **Randomisation process** | | **Deviations from intended interventions** | | **Missing outcome data** | | **Measurement of the outcome** | | **Selection of the reported results** | | **Overall** | |
| Authors' judgement | Support for judgement | Authors' judgement | Support for judgement | Authors' judgement | Support for judgement | Authors' judgement | Support for judgement | Authors' judgement | Support for judgement | Authors' judgement | Support for judgement |
| Kuyucu 2017 | Some concerns | No information about randomisation or baseline differences between groups. | High risk of bias | Unclear if participants knew about their group allocation, no information to understand the effect of assignment, important because of sex based differences in apophysis closure. No information about if there was a deviation from usual practice. The analysis relied on multiple unadjusted statistical tests (including within-group comparisons) without clearly estimating between-group effects | Low risk of bias | Nearly all participant data were reported. | Some concerns | No information on validity of tool for children as primarily an adult score | Some concerns | No pre-specified analysis plan identified. | High risk of bias | No other bias identified |

Risk of bias for analysis 3.4 Physical function in the medium term

| **Study** | **Bias** | | | | | | | | | | | |
| **Randomisation process** | | **Deviations from intended interventions** | | **Missing outcome data** | | **Measurement of the outcome** | | **Selection of the reported results** | | **Overall** | |
| Authors' judgement | Support for judgement | Authors' judgement | Support for judgement | Authors' judgement | Support for judgement | Authors' judgement | Support for judgement | Authors' judgement | Support for judgement | Authors' judgement | Support for judgement |
| Kuyucu 2017 | Some concerns | No information about randomisation or baseline differences between groups. | High risk of bias | Unclear if participants knew about their group allocation, no information to understand the effect of assignment, important because of sex based differences in apophysis closure. No information about if there was a deviation from usual practice. The analysis relied on multiple unadjusted statistical tests (including within-group comparisons) without clearly estimating between-group effects | Low risk of bias | Nearly all participant data were reported. | Some concerns | No information on validity of tool for children as primarily an adult score | Some concerns | No pre-specified analysis plan identified. | High risk of bias | No other bias identified |

Risk of bias for analysis 4.1 Overall pain in the short term

| **Study** | **Bias** | | | | | | | | | | | |
| **Randomisation process** | | **Deviations from intended interventions** | | **Missing outcome data** | | **Measurement of the outcome** | | **Selection of the reported results** | | **Overall** | |
| Authors' judgement | Support for judgement | Authors' judgement | Support for judgement | Authors' judgement | Support for judgement | Authors' judgement | Support for judgement | Authors' judgement | Support for judgement | Authors' judgement | Support for judgement |
| James 2016 | Low risk of bias | Concealed randomised allocation, with only minor differences in gender between one group. | Low risk of bias | Participants, carers and those delivering interventions were likely aware of intervention, and there were no non-protocol interventions, and followed the appropriate analysis plan specified in the protocol | Low risk of bias | Nearly all data available | Low risk of bias | Appropriate outcome measures with anchor questions to reduce subjectivity, without outcome assessor aware of the interventions. | Low risk of bias | Analysis protocol followed | Low risk of bias | No other biases detected |

Risk of bias for analysis 4.2 Overall pain in the medium term

| **Study** | **Bias** | | | | | | | | | | | |
| **Randomisation process** | | **Deviations from intended interventions** | | **Missing outcome data** | | **Measurement of the outcome** | | **Selection of the reported results** | | **Overall** | |
| Authors' judgement | Support for judgement | Authors' judgement | Support for judgement | Authors' judgement | Support for judgement | Authors' judgement | Support for judgement | Authors' judgement | Support for judgement | Authors' judgement | Support for judgement |
| Alfaro-Santafa 2021 | Low risk of bias | Random allocation, concealed until participants were enrolled. | Low risk of bias | Participants were aware of their assigned intervention during the trials. | Low risk of bias | Data available for nearly all participants | High risk of bias | Limited information about the anchor for subjective VAS data. Outcomes described in protocol were described differently in publication. | High risk of bias | Group randomisation and reporting results reported differently from the protocol to the publication. | High risk of bias | Concerns about difference between reporting of results and protocol impacted the overall bias. |

Risk of bias for analysis 4.3 Physical function in the short term

| **Study** | **Bias** | | | | | | | | | | | |
| **Randomisation process** | | **Deviations from intended interventions** | | **Missing outcome data** | | **Measurement of the outcome** | | **Selection of the reported results** | | **Overall** | |
| Authors' judgement | Support for judgement | Authors' judgement | Support for judgement | Authors' judgement | Support for judgement | Authors' judgement | Support for judgement | Authors' judgement | Support for judgement | Authors' judgement | Support for judgement |
| James 2016 | Low risk of bias | Concealed randomised allocation, with only minor differences in gender between one group. | Low risk of bias | Participants, carers and those delivering interventions were likely aware of intervention, and there were no non-protocol interventions, and followed the appropriate analysis plan specified in the protocol | Low risk of bias | Nearly all data available | Low risk of bias | Appropriate outcome measures with instructions to reduce subjectivity, without outcome assessor aware of the interventions. | Low risk of bias | Analysis protocol followed | Low risk of bias | No other bias identified |

Risk of bias for analysis 4.4 Physical function in the medium term

| **Study** | **Bias** | | | | | | | | | | | |
| **Randomisation process** | | **Deviations from intended interventions** | | **Missing outcome data** | | **Measurement of the outcome** | | **Selection of the reported results** | | **Overall** | |
| Authors' judgement | Support for judgement | Authors' judgement | Support for judgement | Authors' judgement | Support for judgement | Authors' judgement | Support for judgement | Authors' judgement | Support for judgement | Authors' judgement | Support for judgement |
| James 2016 | Low risk of bias | Concealed randomised allocation, with only minor differences in gender between one group. | Low risk of bias | Participants, carers and those delivering interventions were likely aware of intervention, and there were no non-protocol interventions, and followed the appropriate analysis plan specified in the protocol | Low risk of bias | Nearly all data available | Low risk of bias | Appropriate outcome measures with instructions to reduce subjectivity, without outcome assessor aware of the interventions. | Low risk of bias | Analysis protocol followed | Low risk of bias | No other biases detected |

Risk of bias for analysis 4.5 Physical function in the long term

| **Study** | **Bias** | | | | | | | | | | | |
| **Randomisation process** | | **Deviations from intended interventions** | | **Missing outcome data** | | **Measurement of the outcome** | | **Selection of the reported results** | | **Overall** | |
| Authors' judgement | Support for judgement | Authors' judgement | Support for judgement | Authors' judgement | Support for judgement | Authors' judgement | Support for judgement | Authors' judgement | Support for judgement | Authors' judgement | Support for judgement |
| James 2016 | Low risk of bias | Concealed randomised allocation, with only minor differences in gender between one group. | Low risk of bias | Participants, carers and those delivering interventions were likely aware of intervention, and there were no non-protocol interventions, and followed the appropriate analysis plan specified in the protocol | Low risk of bias | Nearly all data available | Low risk of bias | Appropriate outcome measures with instructions to reduce subjectivity, without outcome assessor aware of the interventions. | Low risk of bias | Analysis protocol followed | Low risk of bias | No other biases detected |

Risk of bias for analysis 4.6 Adverse events

| **Study** | **Bias** | | | | | | | | | | | |
| **Randomisation process** | | **Deviations from intended interventions** | | **Missing outcome data** | | **Measurement of the outcome** | | **Selection of the reported results** | | **Overall** | |
| Authors' judgement | Support for judgement | Authors' judgement | Support for judgement | Authors' judgement | Support for judgement | Authors' judgement | Support for judgement | Authors' judgement | Support for judgement | Authors' judgement | Support for judgement |
| James 2016 | Low risk of bias | Concealed randomised allocation, with only minor differences in gender between one group. | Low risk of bias | Participants, carers and those delivering interventions were likely aware of intervention, and there were no non-protocol interventions, and followed the appropriate analysis plan specified in the protocol | Low risk of bias | Nearly all data available | Low risk of bias | Appropriate outcome measures without outcome assessor aware of the interventions. | Low risk of bias | Analysis protocol followed | Low risk of bias | No other biases detected |

Risk of bias for analysis 4.8 Joint range of motion in the short term

| **Study** | **Bias** | | | | | | | | | | | |
| **Randomisation process** | | **Deviations from intended interventions** | | **Missing outcome data** | | **Measurement of the outcome** | | **Selection of the reported results** | | **Overall** | |
| Authors' judgement | Support for judgement | Authors' judgement | Support for judgement | Authors' judgement | Support for judgement | Authors' judgement | Support for judgement | Authors' judgement | Support for judgement | Authors' judgement | Support for judgement |
| James 2016 | Low risk of bias | Concealed randomised allocation, with only minor differences in gender between one group. | Low risk of bias | Participants, carers and those delivering interventions were likely aware of intervention, and there were no non-protocol interventions, and followed the appropriate analysis plan specified in the protocol | Low risk of bias | Nearly all data available | Low risk of bias | Appropriate outcome measures without outcome assessor aware of the interventions | Low risk of bias | Analysis protocol followed | Low risk of bias | No other biases detected |

Risk of bias for analysis 5.1 Physical function in the short term

| **Study** | **Bias** | | | | | | | | | | | |
| **Randomisation process** | | **Deviations from intended interventions** | | **Missing outcome data** | | **Measurement of the outcome** | | **Selection of the reported results** | | **Overall** | |
| Authors' judgement | Support for judgement | Authors' judgement | Support for judgement | Authors' judgement | Support for judgement | Authors' judgement | Support for judgement | Authors' judgement | Support for judgement | Authors' judgement | Support for judgement |
| Sweeney 2023 | Low risk of bias | Randomized participants into each group at enrollment using a 1:1 block randomization scheme (block size 4), concealed until assigned, with no group differences. | Some concerns | Participants and carers were aware of group assignment, without deviations due to trial context | Some concerns | Missing data from 11 participants, 16 participants in analysis, potential that missingness impacted outcomes. | Low risk of bias | Data self reported monthly. | High risk of bias | Analysis used Mann-Whitney U test and Hedges g to determine the magnitude of the effect between groups and this was different to the analysis reported within the protocol. | High risk of bias | No other bias identified. |

Risk of bias for analysis 5.3 Adverse events

| **Study** | **Bias** | | | | | | | | | | | |
| **Randomisation process** | | **Deviations from intended interventions** | | **Missing outcome data** | | **Measurement of the outcome** | | **Selection of the reported results** | | **Overall** | |
| Authors' judgement | Support for judgement | Authors' judgement | Support for judgement | Authors' judgement | Support for judgement | Authors' judgement | Support for judgement | Authors' judgement | Support for judgement | Authors' judgement | Support for judgement |
| Sweeney 2023 | Low risk of bias | Randomized participants into each group at enrollment using a 1:1 block randomization scheme (block size 4), concealed until assigned, with no group differences. | Low risk of bias | Participants and carers were aware of group assignment, without deviations due to trial context, with appropriate analysis. | Some concerns | Missing data from 11 participants, 16 participants in analysis, potential that missingness impacted outcomes. | Low risk of bias | Data self reported monthly. | High risk of bias | Analysis used Mann-Whitney U test and Hedges g to determine the magnitude of the effect between groups and this was different to the analysis reported within the protocol. | High risk of bias | No other bias identified. |

Risk of bias for analysis 5.4 Pain during activity in the short term

| **Study** | **Bias** | | | | | | | | | | | |
| **Randomisation process** | | **Deviations from intended interventions** | | **Missing outcome data** | | **Measurement of the outcome** | | **Selection of the reported results** | | **Overall** | |
| Authors' judgement | Support for judgement | Authors' judgement | Support for judgement | Authors' judgement | Support for judgement | Authors' judgement | Support for judgement | Authors' judgement | Support for judgement | Authors' judgement | Support for judgement |
| Sweeney 2023 | Low risk of bias | Randomized participants into each group at enrollment using a 1:1 block randomization scheme (block size 4), concealed until assigned, with no group differences. | Some concerns | Participants and carers were aware of group assignment, without deviations due to trial context | Some concerns | Missing data from 11 participants, 16 participants in analysis, potential that missingness impacted outcomes. | Low risk of bias | Data self reported monthly. | High risk of bias | Analysis used Mann-Whitney U test and Hedges g to determine the magnitude of the effect between groups and this was different to the analysis reported within the protocol. | High risk of bias | No other bias identified. |

Risk of bias for analysis 5.5 Pain during activity in the medium term

| **Study** | **Bias** | | | | | | | | | | | |
| **Randomisation process** | | **Deviations from intended interventions** | | **Missing outcome data** | | **Measurement of the outcome** | | **Selection of the reported results** | | **Overall** | |
| Authors' judgement | Support for judgement | Authors' judgement | Support for judgement | Authors' judgement | Support for judgement | Authors' judgement | Support for judgement | Authors' judgement | Support for judgement | Authors' judgement | Support for judgement |
| Sweeney 2023 | Low risk of bias | Randomized participants into each group at enrollment using a 1:1 block randomization scheme (block size 4), concealed until assigned, with no group differences. | Some concerns | Participants and carers were aware of group assignment, without deviations due to trial context | Some concerns | Missing data from 11 participants, 16 participants in analysis, potential that missingness impacted outcomes. | Low risk of bias | Data self reported monthly | High risk of bias | Analysis used Mann-Whitney U test and Hedges g to determine the magnitude of the effect between groups and this was different to the analysis reported within the protocol. | High risk of bias | No other bias identified. |
