# Supplementary material for: Non‐surgical treatment for lower limb apophyseal injuries
Source: Cochrane Database Syst Rev. 2026 Jul 15;2026(7):CD015156. doi: 10.1002/14651858.CD015156.pub2 (PMC13370774; doi:10.1002/14651858.CD015156.pub2)
Supplement: Supplementary file 6 — Supplementary material 6 Analyses [file CD015156-SUP-06-analyses.html]

Analyses


# Supplementary material 6 to: Non-surgical treatment for lower limb apophyseal injuries

Williams CM, Krommes K, Paterson KL, Haines T, Caserta A, Thorborg K
  
https://doi.org/10.1002/14651858.CD015156.pub2

The material in this section has been supplied by the author(s) for publication under a Licence for Publication and the author(s) are solely responsible for the material. Cochrane has reviewed this material, but Cochrane has not copyedited, formatted or proofread. Cochrane accordingly gives no representations or warranties of any kind in relation to, and accepts no liability for any reliance on or use of, such material.

Back to top

# Analyses

## Analysis group 1: Pharmaceutical interventions versus placebo for children with traction apophysitis of the tibial tubercle

| Analysis or subgroup title | No. of studies | No. of participants | Statistical method | Effect size |
| --- | --- | --- | --- | --- |
| 1.1 Overall pain in the short term | 1 | 23 | Mean Difference (IV, Fixed, 95% CI) | −0.52 [−1.24, 0.20] |
| 1.2 Physical function in the short term | 1 | 19 | Mean Difference (IV, Fixed, 95% CI) | −1.76 [−16.08, 12.56] |
| 1.3 Participation in sport in the short term | 1 | 16 | Mean Difference (IV, Fixed, 95% CI) | 7.90 [−0.41, 16.21] |
| 1.4 Adverse events in the short term | 2 | 74 | Risk Ratio (IV, Random, 95% CI) | 1.31 [0.88, 1.96] |
| 1.5 Pain during an activity in the medium term | 2 | 86 | Std. Mean Difference (IV, Random, 95% CI) | −0.47 [−4.91, 3.97] |
| 1.6 Pain during an activity in the long term | 1 | 34 | Mean Difference (IV, Fixed, 95% CI) | −1.00 [−1.87, −0.13] |
| 1.7 Pain during an activity in the short term | 1 | 43 | Mean Difference (IV, Fixed, 95% CI) | −4.30 [−17.06, 8.46] |

## Analysis group 2: Pharmaceutical interventions versus usual care for children with traction apophysitis of the tibial tubercle

| Analysis or subgroup title | No. of studies | No. of participants | Statistical method | Effect size |
| --- | --- | --- | --- | --- |
| 2.1 Overall pain in the short term | 1 | 21 | Mean Difference (IV, Fixed, 95% CI) | −0.80 [−1.73, 0.13] |
| 2.2 Physical function in the short term | 1 | 16 | Mean Difference (IV, Fixed, 95% CI) | 2.68 [−17.56, 22.92] |
| 2.3 Participation in sport in the short term | 1 | 11 | Mean Difference (IV, Fixed, 95% CI) | 0.85 [−7.13, 8.83] |
| 2.4 Adverse events in the short term | 1 | 30 | Risk Ratio (IV, Fixed, 95% CI) | 1.36 [0.88, 2.10] |
| 2.5 Pain during an activity in the medium term | 1 | 43 | Mean Difference (IV, Fixed, 95% CI) | −2.40 [−3.24, −1.56] |
| 2.6 Pain during an activity in the long term | 1 | 35 | Mean Difference (IV, Fixed, 95% CI) | −2.30 [−3.14, −1.46] |

## Analysis group 3: Taping versus placebo for children with calcaneal apophysitis

| Analysis or subgroup title | No. of studies | No. of participants | Statistical method | Effect size |
| --- | --- | --- | --- | --- |
| 3.1 Overall pain in the short term | 1 | 22 | Mean Difference (IV, Fixed, 95% CI) | 0.10 [−1.25, 1.45] |
| 3.2 Overall pain in the medium term | 1 | 22 | Mean Difference (IV, Fixed, 95% CI) | −1.00 [−1.77, −0.23] |
| 3.3 Physical function in the short term | 1 | 22 | Mean Difference (IV, Fixed, 95% CI) | 6.10 [−0.08, 12.28] |
| 3.4 Physical function in the medium term | 1 | 22 | Mean Difference (IV, Fixed, 95% CI) | 11.20 [5.69, 16.71] |

## Analysis group 4: Foot orthoses versus heel lifts for children with calcaneal apophysitis

| Analysis or subgroup title | No. of studies | No. of participants | Statistical method | Effect size |
| --- | --- | --- | --- | --- |
| 4.1 Overall pain in the short term | 1 | 123 | Mean Difference (IV, Fixed, 95% CI) | 0.00 [−0.44, 0.44] |
| 4.2 Overall pain in the medium term | 1 | 208 | Mean Difference (IV, Fixed, 95% CI) | −55.70 [−60.97, −50.43] |
| 4.3 Physical function in the short term | 1 | 124 | Mean Difference (IV, Fixed, 95% CI) | −1.30 [−7.58, 4.98] |
| 4.4 Physical function in the medium term | 1 | 106 | Mean Difference (IV, Fixed, 95% CI) | −7.80 [−14.22, −1.38] |
| 4.5 Physical function in the long term | 1 | 101 | Mean Difference (IV, Fixed, 95% CI) | −4.30 [−10.68, 2.08] |
| 4.6 Adverse events | 1 | 101 | Risk Ratio (IV, Fixed, 95% CI) | Not estimable |
| 4.7 Pain during activity in the short term | 1 | 44 | Mean Difference (IV, Fixed, 95% CI) | −1.00 [−2.29, 0.29] |
| 4.8 Joint range of motion in the short term | 1 | 124 | Mean Difference (IV, Fixed, 95% CI) | 0.70 [−0.98, 2.38] |

## Analysis group 5: Heel cushioning versus straps for children with calcaneal apophysitis

| Analysis or subgroup title | No. of studies | No. of participants | Statistical method | Effect size |
| --- | --- | --- | --- | --- |
| 5.1 Physical function in the short term | 1 | 43 | Mean Difference (IV, Fixed, 95% CI) | −2.00 [−12.48, 8.48] |
| 5.2 Physical function in the medium term | 1 | 43 | Mean Difference (IV, Fixed, 95% CI) | 2.00 [−7.02, 11.02] |
| 5.3 Adverse events | 1 | 43 | Risk Ratio (IV, Fixed, 95% CI) | 1.05 [0.07, 15.69] |
| 5.4 Pain during activity in the short term | 1 | 43 | Mean Difference (IV, Fixed, 95% CI) | 1.10 [−0.03, 2.23] |
| 5.5 Pain during activity in the medium term | 1 | 43 | Mean Difference (IV, Fixed, 95% CI) | −0.20 [−1.61, 1.21] |

## Analysis group 6: Heel lifts versus no intervention for children with calcaneal apophysitis

| Analysis or subgroup title | No. of studies | No. of participants | Statistical method | Effect size |
| --- | --- | --- | --- | --- |
| 6.1 Self reported treatment success in the short term | 1 | 65 | Mean Difference (IV, Fixed, 95% CI) | 5.00 [−3.19, 13.19] |
| 6.2 Self reported treatment success in the medium term | 1 | 65 | Mean Difference (IV, Fixed, 95% CI) | −5.00 [−11.65, 1.65] |
| 6.3 Pain during an activity in the short term | 1 | 65 | Mean Difference (IV, Fixed, 95% CI) | −0.50 [−1.74, 0.74] |
| 6.4 Pain during an activity in the medium term | 1 | 65 | Mean Difference (IV, Fixed, 95% CI) | −0.40 [−1.79, 0.99] |

## Analysis group 7: Foot orthoses versus usual care for children with calcaneal apophysitis

| Analysis or subgroup title | No. of studies | No. of participants | Statistical method | Effect size |
| --- | --- | --- | --- | --- |
| 7.1 Pain during activity in the short term | 1 | 30 | Mean Difference (IV, Fixed, 95% CI) | −2.25 [−2.98, −1.52] |

## Analysis group 8: Exercise versus usual care for children with calcaneal apophysitis

| Analysis or subgroup title | No. of studies | No. of participants | Statistical method | Effect size |
| --- | --- | --- | --- | --- |
| 8.1 Treatment success in the short term | 1 | 65 | Mean Difference (IV, Fixed, 95% CI) | −1.25 [−9.75, 7.25] |
| 8.2 Treatment success in the medium term | 1 | 65 | Mean Difference (IV, Fixed, 95% CI) | −2.50 [−7.80, 2.80] |
| 8.3 Withdrawals due to adverse events | 1 | 65 | Odds Ratio (IV, Fixed, 95% CI) | Not estimable |
| 8.4 Pain during an activity in the short term | 1 | 65 | Mean Difference (IV, Fixed, 95% CI) | −1.20 [−2.47, 0.07] |
| 8.5 Pain during an activity in the medium term | 1 | 65 | Mean Difference (IV, Fixed, 95% CI) | 0.10 [−1.31, 1.51] |

## Analysis group 9: Exercise versus heel lifts for children with calcaneal apophysitis

| Analysis or subgroup title | No. of studies | No. of participants | Statistical method | Effect size |
| --- | --- | --- | --- | --- |
| 9.1 Self reported treatment success in the short term | 1 | 66 | Mean Difference (IV, Fixed, 95% CI) | −6.25 [−14.66, 2.16] |
| 9.2 Self reported treatment success in the medium term | 1 | 66 | Mean Difference (IV, Fixed, 95% CI) | 2.50 [−4.56, 9.56] |
| 9.3 Pain during activity in the short term | 1 | 66 | Mean Difference (IV, Fixed, 95% CI) | −0.70 [−1.88, 0.48] |
| 9.4 Pain during activity in the medium term | 1 | 66 | Mean Difference (IV, Fixed, 95% CI) | 0.50 [−0.92, 1.92] |

## Analysis group 10: Foot orthoses versus no treatment for children with calcaneal apophysitis

| Analysis or subgroup title | No. of studies | No. of participants | Statistical method | Effect size |
| --- | --- | --- | --- | --- |
| 10.1 Pain during activity in the short term | 1 | 30 | Mean Difference (IV, Fixed, 95% CI) | −2.25 [−2.98, −1.52] |

# Figures and tables

Analysis 1.1: Overall pain in the short term


Study or Subgroup
Reesman 2024

Total
Test for overall effect: Z = 1.42 (P = 0.16)

Heterogeneity: Not applicable

Pharmaceutical
Mean
1.33
SD
0.65
Total
11

11

Placebo
Mean
1.85
SD
1.07
Total
12

12
Weight
100.0%

100.0%

Mean Difference
IV, Fixed, 95% CI
-0.52 [-1.24 , 0.20]

-0.52 [-1.24 , 0.20]

Mean Difference
IV, Fixed, 95% CI


-10

-5

0

5

10


Favours pharmaceutical

Favours placebo


Risk of Bias
A

?
B

−
C

−
D

−
E

?
F

−

Risk of bias legend

(A) Bias arising from the randomization process

(B) Bias due to deviations from intended interventions

(C) Bias due to missing outcome data

(D) Bias in measurement of the outcome

(E) Bias in selection of the reported result

(F) Overall bias


Analysis 1.2: Physical function in the short term


Study or Subgroup
Reesman 2024

Total
Test for overall effect: Z = 0.24 (P = 0.81)

Heterogeneity: Not applicable

Pharmaceutical
Mean
84.66
SD
17.5
Total
8

8

Placebo
Mean
86.42
SD
12.89
Total
11

11
Weight
100.0%

100.0%

Mean Difference
IV, Fixed, 95% CI
-1.76 [-16.08 , 12.56]

-1.76 [-16.08 , 12.56]

Mean Difference
IV, Fixed, 95% CI


-100

-50

0

50

100


Favours pharmaceutical

Favours placebo


Risk of Bias
A

?
B

−
C

−
D

−
E

?
F

−

Risk of bias legend

(A) Bias arising from the randomization process

(B) Bias due to deviations from intended interventions

(C) Bias due to missing outcome data

(D) Bias in measurement of the outcome

(E) Bias in selection of the reported result

(F) Overall bias


Analysis 1.3: Participation in sport in the short term


Study or Subgroup
Reesman 2024

Total
Test for overall effect: Z = 1.86 (P = 0.06)

Heterogeneity: Not applicable

Pharmaceutical
Mean
38.1
SD
9.48
Total
7

7

Placebo
Mean
30.2
SD
6.8
Total
9

9
Weight
100.0%

100.0%

Mean Difference
IV, Fixed, 95% CI
7.90 [-0.41 , 16.21]

7.90 [-0.41 , 16.21]

Mean Difference
IV, Fixed, 95% CI


-100

-50

0

50

100


Favours pharmaceutical

Favours placebo


Risk of Bias
A

?
B

−
C

−
D

−
E

?
F

−

Risk of bias legend

(A) Bias arising from the randomization process

(B) Bias due to deviations from intended interventions

(C) Bias due to missing outcome data

(D) Bias in measurement of the outcome

(E) Bias in selection of the reported result

(F) Overall bias


Analysis 1.4: Adverse events in the short term


Study or Subgroup
Nakase 2020
Reesman 2024

Total
Total events:
Test for overall effect: Z = 1.32 (P = 0.19)

Heterogeneity: Not applicable

Pharmaceutical
Events
0
14
14
Total
22
16

38

Placebo
Events
0
10
10
Total
21
15

36
Weight
100.0%

100.0%

Risk Ratio
IV, Random, 95% CI
Not estimable
1.31 [0.88 , 1.96]

1.31 [0.88 , 1.96]

Risk Ratio
IV, Random, 95% CI


0.01

0.1

1

10

100


Favours pharmaceutical

Favours placebo


Risk of Bias
A

?

?
B

+

−
C

+

−
D

?

?
E

?

?
F

?

−

Risk of bias legend

(A) Bias arising from the randomization process

(B) Bias due to deviations from intended interventions

(C) Bias due to missing outcome data

(D) Bias in measurement of the outcome

(E) Bias in selection of the reported result

(F) Overall bias


Analysis 1.5: Pain during an activity in the medium term


Study or Subgroup
Nakase 2020
Topol 2011

Total (HKSJ
a
)
Test for overall effect: T = 1.35, df = 1 (P = 0.41)

Heterogeneity: Tau² (REML
b
) = 0.15; Chi² = 2.50, df = 1 (P = 0.11); I² = 60%

Pharmaceutical
Mean
14.3
0.7
SD
18.7
1.2
Total
22
21

43

Placebo
Mean
16.8
1.8
SD
19.8
1.4
Total
21
22

43
Weight
50.9%
49.1%

100.0%

Std. Mean Difference
IV, Random, 95% CI
-0.13 [-0.73 , 0.47]
-0.83 [-1.45 , -0.20]

-0.47 [-4.91 , 3.97]

Std. Mean Difference
IV, Random, 95% CI


-100

-50

0

50

100


Favours pharmaceutical

Favours placebo


Risk of Bias
A

?

+
B

+

?
C

+

+
D

?

+
E

?

?
F

?

?

Footnotes

a
CI calculated by Hartung-Knapp-Sidik-Jonkman (HKSJ) method.

b
Tau² calculated by Restricted Maximum-Likelihood method.
Risk of bias legend

(A) Bias arising from the randomization process

(B) Bias due to deviations from intended interventions

(C) Bias due to missing outcome data

(D) Bias in measurement of the outcome

(E) Bias in selection of the reported result

(F) Overall bias


Analysis 1.6: Pain during an activity in the long term


Study or Subgroup
Topol 2011

Total
Test for overall effect: Z = 2.26 (P = 0.02)

Heterogeneity: Not applicable

Pharmaceutical
Mean
0.2
SD
0.7
Total
21

21

Placebo
Mean
1.2
SD
1.5
Total
13

13
Weight
100.0%

100.0%

Mean Difference
IV, Fixed, 95% CI
-1.00 [-1.87 , -0.13]

-1.00 [-1.87 , -0.13]

Mean Difference
IV, Fixed, 95% CI


-100

-50

0

50

100


Favours pharmaceutical

Favours placebo


Risk of Bias
A

+
B

?
C

+
D

+
E

?
F

?

Risk of bias legend

(A) Bias arising from the randomization process

(B) Bias due to deviations from intended interventions

(C) Bias due to missing outcome data

(D) Bias in measurement of the outcome

(E) Bias in selection of the reported result

(F) Overall bias


Analysis 1.7: Pain during an activity in the short term


Study or Subgroup
Nakase 2020

Total
Test for overall effect: Z = 0.66 (P = 0.51)

Heterogeneity: Not applicable

Pharmaceutical
Mean
23.1
SD
20.4
Total
22

22

Placebo
Mean
27.4
SD
22.2
Total
21

21
Weight
100.0%

100.0%

Mean Difference
IV, Fixed, 95% CI
-4.30 [-17.06 , 8.46]

-4.30 [-17.06 , 8.46]

Mean Difference
IV, Fixed, 95% CI


-100

-50

0

50

100


Favours pharmaceutical

Favours placebo


Risk of Bias
A

?
B

+
C

+
D

?
E

?
F

?

Risk of bias legend

(A) Bias arising from the randomization process

(B) Bias due to deviations from intended interventions

(C) Bias due to missing outcome data

(D) Bias in measurement of the outcome

(E) Bias in selection of the reported result

(F) Overall bias


Analysis 2.1: Overall pain in the short term


Study or Subgroup
Reesman 2024

Total
Test for overall effect: Z = 1.69 (P = 0.09)

Heterogeneity: Not applicable

Pharmaceutical
Mean
1.33
SD
0.65
Total
11

11

Usual care or no treatment
Mean
2.13
SD
1.36
Total
10

10
Weight
100.0%

100.0%

Mean Difference
IV, Fixed, 95% CI
-0.80 [-1.73 , 0.13]

-0.80 [-1.73 , 0.13]

Mean Difference
IV, Fixed, 95% CI


-10

-5

0

5

10


Favours pharmaceutical

Favours usual care


Risk of Bias
A

?
B

−
C

−
D

−
E

?
F

−

Risk of bias legend

(A) Bias arising from the randomization process

(B) Bias due to deviations from intended interventions

(C) Bias due to missing outcome data

(D) Bias in measurement of the outcome

(E) Bias in selection of the reported result

(F) Overall bias


Analysis 2.2: Physical function in the short term


Study or Subgroup
Reesman 2024

Total
Test for overall effect: Z = 0.26 (P = 0.80)

Heterogeneity: Not applicable

Pharmaceutical
Mean
84.66
SD
17.5
Total
8

8

Usual care or no treatment
Mean
81.98
SD
23.38
Total
8

8
Weight
100.0%

100.0%

Mean Difference
IV, Fixed, 95% CI
2.68 [-17.56 , 22.92]

2.68 [-17.56 , 22.92]

Mean Difference
IV, Fixed, 95% CI


-100

-50

0

50

100


Favours pharmaceutical

Favours usual care


Risk of Bias
A

?
B

−
C

−
D

−
E

?
F

−

Risk of bias legend

(A) Bias arising from the randomization process

(B) Bias due to deviations from intended interventions

(C) Bias due to missing outcome data

(D) Bias in measurement of the outcome

(E) Bias in selection of the reported result

(F) Overall bias


Analysis 2.3: Participation in sport in the short term


Study or Subgroup
Reesman 2024

Total
Test for overall effect: Z = 0.21 (P = 0.83)

Heterogeneity: Not applicable

Pharmaceutical
Mean
38.1
SD
9.48
Total
7

7

Usual care or no treatment
Mean
37.25
SD
3.86
Total
4

4
Weight
100.0%

100.0%

Mean Difference
IV, Fixed, 95% CI
0.85 [-7.13 , 8.83]

0.85 [-7.13 , 8.83]

Mean Difference
IV, Fixed, 95% CI


-50

-25

0

25

50


Favours pharmaceutical

Favours usual care


Risk of Bias
A

?
B

−
C

−
D

−
E

?
F

−

Risk of bias legend

(A) Bias arising from the randomization process

(B) Bias due to deviations from intended interventions

(C) Bias due to missing outcome data

(D) Bias in measurement of the outcome

(E) Bias in selection of the reported result

(F) Overall bias


Analysis 2.4: Adverse events in the short term


Study or Subgroup
Reesman 2024

Total
Total events:
Test for overall effect: Z = 1.40 (P = 0.16)

Heterogeneity: Not applicable

Pharmaceutical
Events
14
14
Total
16

16

Usual care or no treatment
Events
9
9
Total
14

14
Weight
100.0%

100.0%

Risk Ratio
IV, Fixed, 95% CI
1.36 [0.88 , 2.10]

1.36 [0.88 , 2.10]

Risk Ratio
IV, Fixed, 95% CI


0.01

0.1

1

10

100


Favours pharmaceutical

Favours usual care


Risk of Bias
A

?
B

−
C

−
D

?
E

?
F

−

Risk of bias legend

(A) Bias arising from the randomization process

(B) Bias due to deviations from intended interventions

(C) Bias due to missing outcome data

(D) Bias in measurement of the outcome

(E) Bias in selection of the reported result

(F) Overall bias


Analysis 2.5: Pain during an activity in the medium term


Study or Subgroup
Topol 2011

Total
Test for overall effect: Z = 5.58 (P < 0.00001)

Heterogeneity: Not applicable

Pharmaceutical
Mean
0.7
SD
1.2
Total
21

21

Usual care or no treatment
Mean
3.1
SD
1.6
Total
22

22
Weight
100.0%

100.0%

Mean Difference
IV, Fixed, 95% CI
-2.40 [-3.24 , -1.56]

-2.40 [-3.24 , -1.56]

Mean Difference
IV, Fixed, 95% CI


-4

-2

0

2

4


Favours pharmaceutical

Favours usual care


Risk of Bias
A

+
B

?
C

+
D

+
E

?
F

?

Risk of bias legend

(A) Bias arising from the randomization process

(B) Bias due to deviations from intended interventions

(C) Bias due to missing outcome data

(D) Bias in measurement of the outcome

(E) Bias in selection of the reported result

(F) Overall bias


Analysis 2.6: Pain during an activity in the long term


Study or Subgroup
Topol 2011

Total
Test for overall effect: Z = 5.36 (P < 0.00001)

Heterogeneity: Not applicable

Pharmaceutical
Mean
0.2
SD
0.7
Total
21

21

Usual care or no treatment
Mean
2.5
SD
1.5
Total
14

14
Weight
100.0%

100.0%

Mean Difference
IV, Fixed, 95% CI
-2.30 [-3.14 , -1.46]

-2.30 [-3.14 , -1.46]

Mean Difference
IV, Fixed, 95% CI


-100

-50

0

50

100


Favours pharmaceutical

Favours usual care or no treatment


Risk of Bias
A

+
B

?
C

+
D

+
E

?
F

?

Risk of bias legend

(A) Bias arising from the randomization process

(B) Bias due to deviations from intended interventions

(C) Bias due to missing outcome data

(D) Bias in measurement of the outcome

(E) Bias in selection of the reported result

(F) Overall bias


Analysis 3.1: Overall pain in the short term


Study or Subgroup
Kuyucu 2017

Total
Test for overall effect: Z = 0.15 (P = 0.88)

Heterogeneity: Not applicable

Foot orthoses, bracing, taping or straps
Mean
5.5
SD
0.9
Total
11

11

Placebo
Mean
5.4
SD
2.1
Total
11

11
Weight
100.0%

100.0%

Mean Difference
IV, Fixed, 95% CI
0.10 [-1.25 , 1.45]

0.10 [-1.25 , 1.45]

Mean Difference
IV, Fixed, 95% CI


-10

-5

0

5

10


Favours taping

Favours placebo


Risk of Bias
A

?
B

−
C

+
D

?
E

?
F

−

Risk of bias legend

(A) Bias arising from the randomization process

(B) Bias due to deviations from intended interventions

(C) Bias due to missing outcome data

(D) Bias in measurement of the outcome

(E) Bias in selection of the reported result

(F) Overall bias


Analysis 3.2: Overall pain in the medium term


Study or Subgroup
Kuyucu 2017

Total
Test for overall effect: Z = 2.55 (P = 0.01)

Heterogeneity: Not applicable

Foot orthoses, bracing, taping or straps
Mean
0.5
SD
0.5
Total
11

11

Placebo
Mean
1.5
SD
1.2
Total
11

11
Weight
100.0%

100.0%

Mean Difference
IV, Fixed, 95% CI
-1.00 [-1.77 , -0.23]

-1.00 [-1.77 , -0.23]

Mean Difference
IV, Fixed, 95% CI


-10

-5

0

5

10


Favours taping

Favours placebo


Risk of Bias
A

?
B

−
C

+
D

?
E

?
F

−

Risk of bias legend

(A) Bias arising from the randomization process

(B) Bias due to deviations from intended interventions

(C) Bias due to missing outcome data

(D) Bias in measurement of the outcome

(E) Bias in selection of the reported result

(F) Overall bias


Analysis 3.3: Physical function in the short term


Study or Subgroup
Kuyucu 2017

Total
Test for overall effect: Z = 1.93 (P = 0.05)

Heterogeneity: Not applicable

Foot orthoses, bracing, taping or straps
Mean
83.5
SD
5.5
Total
11

11

Placebo
Mean
77.4
SD
8.9
Total
11

11
Weight
100.0%

100.0%

Mean Difference
IV, Fixed, 95% CI
6.10 [-0.08 , 12.28]

6.10 [-0.08 , 12.28]

Mean Difference
IV, Fixed, 95% CI


-100

-50

0

50

100


Favours taping

Favours placebo


Risk of Bias
A

?
B

−
C

+
D

?
E

?
F

−

Risk of bias legend

(A) Bias arising from the randomization process

(B) Bias due to deviations from intended interventions

(C) Bias due to missing outcome data

(D) Bias in measurement of the outcome

(E) Bias in selection of the reported result

(F) Overall bias


Analysis 3.4: Physical function in the medium term


Study or Subgroup
Kuyucu 2017

Total
Test for overall effect: Z = 3.98 (P < 0.0001)

Heterogeneity: Not applicable

Foot orthoses, bracing, taping or straps
Mean
98.3
SD
3.1
Total
11

11

Placebo
Mean
87.1
SD
8.8
Total
11

11
Weight
100.0%

100.0%

Mean Difference
IV, Fixed, 95% CI
11.20 [5.69 , 16.71]

11.20 [5.69 , 16.71]

Mean Difference
IV, Fixed, 95% CI


-100

-50

0

50

100


Favours taping

Favours placebo


Risk of Bias
A

?
B

−
C

+
D

?
E

?
F

−

Risk of bias legend

(A) Bias arising from the randomization process

(B) Bias due to deviations from intended interventions

(C) Bias due to missing outcome data

(D) Bias in measurement of the outcome

(E) Bias in selection of the reported result

(F) Overall bias


Analysis 4.1: Overall pain in the short term


Study or Subgroup
James 2016

Total
Test for overall effect: Z = 0.00 (P = 1.00)

Heterogeneity: Not applicable

Foot orthoses, bracing, taping or straps
Mean
2.9
SD
1.3
Total
62

62

Heel lifts
Mean
2.9
SD
1.2
Total
61

61
Weight
100.0%

100.0%

Mean Difference
IV, Fixed, 95% CI
0.00 [-0.44 , 0.44]

0.00 [-0.44 , 0.44]

Mean Difference
IV, Fixed, 95% CI


-4

-2

0

2

4


Favours foot orthoses

Favours heel lifts


Risk of Bias
A

+
B

+
C

+
D

+
E

+
F

+

Risk of bias legend

(A) Bias arising from the randomization process

(B) Bias due to deviations from intended interventions

(C) Bias due to missing outcome data

(D) Bias in measurement of the outcome

(E) Bias in selection of the reported result

(F) Overall bias


Analysis 4.2: Overall pain in the medium term


Study or Subgroup
Alfaro-Santafa 2021

Total
Test for overall effect: Z = 20.71 (P < 0.00001)

Heterogeneity: Not applicable

Foot orthoses, bracing, taping or straps
Mean
11.6
SD
17.4
Total
104

104

Heel lifts
Mean
67.3
SD
21.2
Total
104

104
Weight
100.0%

100.0%

Mean Difference
IV, Fixed, 95% CI
-55.70 [-60.97 , -50.43]

-55.70 [-60.97 , -50.43]

Mean Difference
IV, Fixed, 95% CI


-100

-50

0

50

100


Favours foot orthoses

Favours heel lifts


Risk of Bias
A

+
B

+
C

+
D

−
E

−
F

−

Risk of bias legend

(A) Bias arising from the randomization process

(B) Bias due to deviations from intended interventions

(C) Bias due to missing outcome data

(D) Bias in measurement of the outcome

(E) Bias in selection of the reported result

(F) Overall bias


Analysis 4.3: Physical function in the short term


Study or Subgroup
James 2016

Total
Test for overall effect: Z = 0.41 (P = 0.68)

Heterogeneity: Not applicable

Foot orthoses, bracing, taping or straps
Mean
63.1
SD
16.7
Total
62

62

Heel lifts
Mean
64.4
SD
18.9
Total
62

62
Weight
100.0%

100.0%

Mean Difference
IV, Fixed, 95% CI
-1.30 [-7.58 , 4.98]

-1.30 [-7.58 , 4.98]

Mean Difference
IV, Fixed, 95% CI


-100

-50

0

50

100


Favours foot orthoses

Favours heel lifts


Risk of Bias
A

+
B

+
C

+
D

+
E

+
F

+

Risk of bias legend

(A) Bias arising from the randomization process

(B) Bias due to deviations from intended interventions

(C) Bias due to missing outcome data

(D) Bias in measurement of the outcome

(E) Bias in selection of the reported result

(F) Overall bias


Analysis 4.4: Physical function in the medium term


Study or Subgroup
James 2016

Total
Test for overall effect: Z = 2.38 (P = 0.02)

Heterogeneity: Not applicable

Foot orthoses, bracing, taping or straps
Mean
74.2
SD
17.5
Total
55

55

Heel lifts
Mean
82
SD
16.2
Total
51

51
Weight
100.0%

100.0%

Mean Difference
IV, Fixed, 95% CI
-7.80 [-14.22 , -1.38]

-7.80 [-14.22 , -1.38]

Mean Difference
IV, Fixed, 95% CI


-100

-50

0

50

100


Favours foot orthoses

Favours heel lifts


Risk of Bias
A

+
B

+
C

+
D

+
E

+
F

+

Risk of bias legend

(A) Bias arising from the randomization process

(B) Bias due to deviations from intended interventions

(C) Bias due to missing outcome data

(D) Bias in measurement of the outcome

(E) Bias in selection of the reported result

(F) Overall bias


Analysis 4.5: Physical function in the long term


Study or Subgroup
James 2016

Total
Test for overall effect: Z = 1.32 (P = 0.19)

Heterogeneity: Not applicable

Foot orthoses, bracing, taping or straps
Mean
79.5
SD
18.9
Total
51

51

Heel lifts
Mean
83.8
SD
13.4
Total
50

50
Weight
100.0%

100.0%

Mean Difference
IV, Fixed, 95% CI
-4.30 [-10.68 , 2.08]

-4.30 [-10.68 , 2.08]

Mean Difference
IV, Fixed, 95% CI


-100

-50

0

50

100


Favours foot orthoses

Favours heel lifts


Risk of Bias
A

+
B

+
C

+
D

+
E

+
F

+

Risk of bias legend

(A) Bias arising from the randomization process

(B) Bias due to deviations from intended interventions

(C) Bias due to missing outcome data

(D) Bias in measurement of the outcome

(E) Bias in selection of the reported result

(F) Overall bias


Analysis 4.6: Adverse events


Study or Subgroup
James 2016

Total
Total events:
Test for overall effect: Not applicable

Heterogeneity: Not applicable

Foot orthoses, bracing, taping or straps
Events
0
0
Total
51

51

Heel lifts
Events
0
0
Total
50

50
Weight

Risk Ratio
IV, Fixed, 95% CI
Not estimable

Not estimable

Risk Ratio
IV, Fixed, 95% CI


0.01

0.1

1

10

100


Favours foot orthoses

Favours heel lifts

Risk of Bias
A

+
B

+
C

+
D

+
E

+
F

+

Risk of bias legend

(A) Bias arising from the randomization process

(B) Bias due to deviations from intended interventions

(C) Bias due to missing outcome data

(D) Bias in measurement of the outcome

(E) Bias in selection of the reported result

(F) Overall bias


Analysis 4.7: Pain during activity in the short term


Study or Subgroup
Perhamre 2011a

Total
Test for overall effect: Z = 1.52 (P = 0.13)

Heterogeneity: Not applicable

Foot orthoses, bracing, taping or straps
Mean
2.17
SD
1.99
Total
20

20

Heel lifts
Mean
3.17
SD
2.36
Total
24

24
Weight
100.0%

100.0%

Mean Difference
IV, Fixed, 95% CI
-1.00 [-2.29 , 0.29]

-1.00 [-2.29 , 0.29]

Mean Difference
IV, Fixed, 95% CI


-100

-50

0

50

100


Favours foot orthoses

Favours heel lifts


Risk of Bias
A

+
B

?
C

+
D

?
E

?
F

?

Risk of bias legend

(A) Bias arising from the randomization process

(B) Bias due to deviations from intended interventions

(C) Bias due to missing outcome data

(D) Bias in measurement of the outcome

(E) Bias in selection of the reported result

(F) Overall bias


Analysis 4.8: Joint range of motion in the short term


Study or Subgroup
James 2016

Total
Test for overall effect: Z = 0.82 (P = 0.41)

Heterogeneity: Not applicable

Foot orthoses, bracing, taping or straps
Mean
31.6
SD
4.4
Total
62

62

Heel lifts
Mean
30.9
SD
5.1
Total
62

62
Weight
100.0%

100.0%

Mean Difference
IV, Fixed, 95% CI
0.70 [-0.98 , 2.38]

0.70 [-0.98 , 2.38]

Mean Difference
IV, Fixed, 95% CI


-50

-25

0

25

50


Favours heel lifts

Favours foot orthoses


Risk of Bias
A

+
B

+
C

+
D

+
E

+
F

+

Risk of bias legend

(A) Bias arising from the randomization process

(B) Bias due to deviations from intended interventions

(C) Bias due to missing outcome data

(D) Bias in measurement of the outcome

(E) Bias in selection of the reported result

(F) Overall bias


Analysis 5.1: Physical function in the short term


Study or Subgroup
Sweeney 2023

Total
Test for overall effect: Z = 0.37 (P = 0.71)

Heterogeneity: Not applicable

Footwear or heel cushioning
Mean
76
SD
16
Total
21

21

Foot orthoses, bracing, taping or straps
Mean
78
SD
19
Total
22

22
Weight
100.0%

100.0%

Mean Difference
IV, Fixed, 95% CI
-2.00 [-12.48 , 8.48]

-2.00 [-12.48 , 8.48]

Mean Difference
IV, Fixed, 95% CI


-100

-50

0

50

100


Favours strap

Favours heel cushioning


Risk of Bias
A

+
B

?
C

?
D

+
E

−
F

−

Risk of bias legend

(A) Bias arising from the randomization process

(B) Bias due to deviations from intended interventions

(C) Bias due to missing outcome data

(D) Bias in measurement of the outcome

(E) Bias in selection of the reported result

(F) Overall bias


Analysis 5.2: Physical function in the medium term


Study or Subgroup
Sweeney 2023

Total
Test for overall effect: Z = 0.43 (P = 0.66)

Heterogeneity: Not applicable

Footwear or heel cushioning
Mean
89
SD
13
Total
21

21

Foot orthoses, bracing, taping or straps
Mean
87
SD
17
Total
22

22
Weight
100.0%

100.0%

Mean Difference
IV, Fixed, 95% CI
2.00 [-7.02 , 11.02]

2.00 [-7.02 , 11.02]

Mean Difference
IV, Fixed, 95% CI


-100

-50

0

50

100


Favours strap

Favours heel cushioning


Risk of Bias
A

+
B

?
C

?
D

+
E

−
F

−

Risk of bias legend

(A) Bias arising from the randomization process

(B) Bias due to deviations from intended interventions

(C) Bias due to missing outcome data

(D) Bias in measurement of the outcome

(E) Bias in selection of the reported result

(F) Overall bias


Analysis 5.3: Adverse events


Study or Subgroup
Sweeney 2023

Total
Total events:
Test for overall effect: Z = 0.03 (P = 0.97)

Heterogeneity: Not applicable

Footwear or heel cushioning
Events
1
1
Total
21

21

Foot orthoses, bracing, taping or straps
Events
1
1
Total
22

22
Weight
100.0%

100.0%

Risk Ratio
IV, Fixed, 95% CI
1.05 [0.07 , 15.69]

1.05 [0.07 , 15.69]

Risk Ratio
IV, Fixed, 95% CI


0.01

0.1

1

10

100


Favours strap

Favours heel cushioning


Risk of Bias
A

+
B

+
C

?
D

+
E

−
F

−

Risk of bias legend

(A) Bias arising from the randomization process

(B) Bias due to deviations from intended interventions

(C) Bias due to missing outcome data

(D) Bias in measurement of the outcome

(E) Bias in selection of the reported result

(F) Overall bias


Analysis 5.4: Pain during activity in the short term


Study or Subgroup
Sweeney 2023

Total
Test for overall effect: Z = 1.90 (P = 0.06)

Heterogeneity: Not applicable

Footwear or heel cushioning
Mean
6.6
SD
1.4
Total
21

21

Foot orthoses, bracing, taping or straps
Mean
5.5
SD
2.3
Total
22

22
Weight
100.0%

100.0%

Mean Difference
IV, Fixed, 95% CI
1.10 [-0.03 , 2.23]

1.10 [-0.03 , 2.23]

Mean Difference
IV, Fixed, 95% CI


-10

-5

0

5

10


Favours strap

Favours heel cushioning


Risk of Bias
A

+
B

?
C

?
D

+
E

−
F

−

Risk of bias legend

(A) Bias arising from the randomization process

(B) Bias due to deviations from intended interventions

(C) Bias due to missing outcome data

(D) Bias in measurement of the outcome

(E) Bias in selection of the reported result

(F) Overall bias


Analysis 5.5: Pain during activity in the medium term


Study or Subgroup
Sweeney 2023

Total
Test for overall effect: Z = 0.28 (P = 0.78)

Heterogeneity: Not applicable

Footwear or heel cushioning
Mean
3.4
SD
2.1
Total
21

21

Foot orthoses, bracing, taping or straps
Mean
3.6
SD
2.6
Total
22

22
Weight
100.0%

100.0%

Mean Difference
IV, Fixed, 95% CI
-0.20 [-1.61 , 1.21]

-0.20 [-1.61 , 1.21]

Mean Difference
IV, Fixed, 95% CI


-10

-5

0

5

10


Favours strap

Favours heel cushioning


Risk of Bias
A

+
B

?
C

?
D

+
E

−
F

−

Risk of bias legend

(A) Bias arising from the randomization process

(B) Bias due to deviations from intended interventions

(C) Bias due to missing outcome data

(D) Bias in measurement of the outcome

(E) Bias in selection of the reported result

(F) Overall bias


Analysis 6.1: Self reported treatment success in the short term


Study or Subgroup
Wiegerinck 2016

Total
Test for overall effect: Z = 1.20 (P = 0.23)

Heterogeneity: Not applicable

Heel lifts
Mean
72.5
SD
16.8
Total
33

33

Usual care or no treatment
Mean
67.5
SD
16.9
Total
32

32
Weight
100.0%

100.0%

Mean Difference
IV, Fixed, 95% CI
5.00 [-3.19 , 13.19]

5.00 [-3.19 , 13.19]

Mean Difference
IV, Fixed, 95% CI


-100

-50

0

50

100


Favours heel lifts

Favours no treatment


Risk of Bias
A

+
B

?
C

+
D

+
E

?
F

?

Risk of bias legend

(A) Bias arising from the randomization process

(B) Bias due to deviations from intended interventions

(C) Bias due to missing outcome data

(D) Bias in measurement of the outcome

(E) Bias in selection of the reported result

(F) Overall bias


Analysis 6.2: Self reported treatment success in the medium term


Study or Subgroup
Wiegerinck 2016

Total
Test for overall effect: Z = 1.47 (P = 0.14)

Heterogeneity: Not applicable

Heel lifts
Mean
75
SD
16.83
Total
33

33

Usual care or no treatment
Mean
80
SD
9.68
Total
32

32
Weight
100.0%

100.0%

Mean Difference
IV, Fixed, 95% CI
-5.00 [-11.65 , 1.65]

-5.00 [-11.65 , 1.65]

Mean Difference
IV, Fixed, 95% CI


-100

-50

0

50

100


Favours heel lifts

Favours no treatment


Risk of Bias
A

+
B

?
C

+
D

+
E

?
F

?

Risk of bias legend

(A) Bias arising from the randomization process

(B) Bias due to deviations from intended interventions

(C) Bias due to missing outcome data

(D) Bias in measurement of the outcome

(E) Bias in selection of the reported result

(F) Overall bias


Analysis 6.3: Pain during an activity in the short term


Study or Subgroup
Wiegerinck 2016

Total
Test for overall effect: Z = 0.79 (P = 0.43)

Heterogeneity: Not applicable

Heel lifts
Mean
-2.5
SD
2.4
Total
33

33

Usual care or no treatment
Mean
-2
SD
2.7
Total
32

32
Weight
100.0%

100.0%

Mean Difference
IV, Fixed, 95% CI
-0.50 [-1.74 , 0.74]

-0.50 [-1.74 , 0.74]

Mean Difference
IV, Fixed, 95% CI


-10

-5

0

5

10


Favours heel lifts

Favours no treatment


Risk of Bias
A

+
B

?
C

+
D

+
E

?
F

?

Risk of bias legend

(A) Bias arising from the randomization process

(B) Bias due to deviations from intended interventions

(C) Bias due to missing outcome data

(D) Bias in measurement of the outcome

(E) Bias in selection of the reported result

(F) Overall bias


Analysis 6.4: Pain during an activity in the medium term


Study or Subgroup
Wiegerinck 2016

Total
Test for overall effect: Z = 0.57 (P = 0.57)

Heterogeneity: Not applicable

Heel lifts
Mean
-4.3
SD
2.9
Total
33

33

Usual care or no treatment
Mean
-3.9
SD
2.8
Total
32

32
Weight
100.0%

100.0%

Mean Difference
IV, Fixed, 95% CI
-0.40 [-1.79 , 0.99]

-0.40 [-1.79 , 0.99]

Mean Difference
IV, Fixed, 95% CI


-10

-5

0

5

10


Favours heel lifts

Favours no treatment


Risk of Bias
A

+
B

?
C

+
D

+
E

?
F

?

Risk of bias legend

(A) Bias arising from the randomization process

(B) Bias due to deviations from intended interventions

(C) Bias due to missing outcome data

(D) Bias in measurement of the outcome

(E) Bias in selection of the reported result

(F) Overall bias


Analysis 7.1: Pain during activity in the short term


Study or Subgroup
Perhamre 2012

Total
Test for overall effect: Z = 6.02 (P < 0.00001)

Heterogeneity: Not applicable

Foot orthoses, bracing, taping or straps
Mean
0.25
SD
0.14
Total
15

15

Usual care or no treatment
Mean
2.5
SD
1.44
Total
15

15
Weight
100.0%

100.0%

Mean Difference
IV, Fixed, 95% CI
-2.25 [-2.98 , -1.52]

-2.25 [-2.98 , -1.52]

Mean Difference
IV, Fixed, 95% CI


-100

-50

0

50

100


Favours foot orthoses

Favours usual care


Risk of Bias
A

?
B

?
C

+
D

+
E

?
F

?

Risk of bias legend

(A) Bias arising from the randomization process

(B) Bias due to deviations from intended interventions

(C) Bias due to missing outcome data

(D) Bias in measurement of the outcome

(E) Bias in selection of the reported result

(F) Overall bias


Analysis 8.1: Treatment success in the short term


Study or Subgroup
Wiegerinck 2016

Total
Test for overall effect: Z = 0.29 (P = 0.77)

Heterogeneity: Not applicable

Exercise
Mean
66.25
SD
18.04
Total
33

33

Usual care or no treatment
Mean
67.5
SD
16.9
Total
32

32
Weight
100.0%

100.0%

Mean Difference
IV, Fixed, 95% CI
-1.25 [-9.75 , 7.25]

-1.25 [-9.75 , 7.25]

Mean Difference
IV, Fixed, 95% CI


-10

-5

0

5

10


Favours exercise

Favours no treatment


Risk of Bias
A

+
B

?
C

+
D

+
E

?
F

?

Risk of bias legend

(A) Bias arising from the randomization process

(B) Bias due to deviations from intended interventions

(C) Bias due to missing outcome data

(D) Bias in measurement of the outcome

(E) Bias in selection of the reported result

(F) Overall bias


Analysis 8.2: Treatment success in the medium term


Study or Subgroup
Wiegerinck 2016

Total
Test for overall effect: Z = 0.92 (P = 0.36)

Heterogeneity: Not applicable

Exercise
Mean
77.5
SD
12.02
Total
33

33

Usual care or no treatment
Mean
80
SD
9.68
Total
32

32
Weight
100.0%

100.0%

Mean Difference
IV, Fixed, 95% CI
-2.50 [-7.80 , 2.80]

-2.50 [-7.80 , 2.80]

Mean Difference
IV, Fixed, 95% CI


-100

-50

0

50

100


Favours exercise

Favours no treatment


Risk of Bias
A

+
B

?
C

+
D

+
E

?
F

?

Risk of bias legend

(A) Bias arising from the randomization process

(B) Bias due to deviations from intended interventions

(C) Bias due to missing outcome data

(D) Bias in measurement of the outcome

(E) Bias in selection of the reported result

(F) Overall bias


Analysis 8.3: Withdrawals due to adverse events


Study or Subgroup
Wiegerinck 2016

Total
Total events:
Test for overall effect: Not applicable

Heterogeneity: Not applicable

Exercise
Events
0
0
Total
33

33

Usual care or no treatment
Events
0
0
Total
32

32
Weight

Odds Ratio
IV, Fixed, 95% CI
Not estimable

Not estimable

Odds Ratio
IV, Fixed, 95% CI


0.01

0.1

1

10

100


Favours exercise

Favours usual care or no treatment

Risk of Bias
A

+
B

?
C

+
D

+
E

?
F

?

Risk of bias legend

(A) Bias arising from the randomization process

(B) Bias due to deviations from intended interventions

(C) Bias due to missing outcome data

(D) Bias in measurement of the outcome

(E) Bias in selection of the reported result

(F) Overall bias


Analysis 8.4: Pain during an activity in the short term


Study or Subgroup
Wiegerinck 2016

Total
Test for overall effect: Z = 1.86 (P = 0.06)

Heterogeneity: Not applicable

Exercise
Mean
-3.2
SD
2.5
Total
33

33

Usual care or no treatment
Mean
-2
SD
2.7
Total
32

32
Weight
100.0%

100.0%

Mean Difference
IV, Fixed, 95% CI
-1.20 [-2.47 , 0.07]

-1.20 [-2.47 , 0.07]

Mean Difference
IV, Fixed, 95% CI


-10

-5

0

5

10


Favours exercise

Favours no treatment


Risk of Bias
A

+
B

?
C

+
D

+
E

?
F

?

Risk of bias legend

(A) Bias arising from the randomization process

(B) Bias due to deviations from intended interventions

(C) Bias due to missing outcome data

(D) Bias in measurement of the outcome

(E) Bias in selection of the reported result

(F) Overall bias


Analysis 8.5: Pain during an activity in the medium term


Study or Subgroup
Wiegerinck 2016

Total
Test for overall effect: Z = 0.14 (P = 0.89)

Heterogeneity: Not applicable

Exercise
Mean
-3.8
SD
3
Total
33

33

Usual care or no treatment
Mean
-3.9
SD
2.8
Total
32

32
Weight
100.0%

100.0%

Mean Difference
IV, Fixed, 95% CI
0.10 [-1.31 , 1.51]

0.10 [-1.31 , 1.51]

Mean Difference
IV, Fixed, 95% CI


-10

-5

0

5

10


Favours exercise

Favours no treatment


Risk of Bias
A

+
B

?
C

+
D

+
E

?
F

?

Risk of bias legend

(A) Bias arising from the randomization process

(B) Bias due to deviations from intended interventions

(C) Bias due to missing outcome data

(D) Bias in measurement of the outcome

(E) Bias in selection of the reported result

(F) Overall bias


Analysis 9.1: Self reported treatment success in the short term


Study or Subgroup
Wiegerinck 2016

Total
Test for overall effect: Z = 1.46 (P = 0.15)

Heterogeneity: Not applicable

Exercise
Mean
66.25
SD
18.04
Total
33

33

Heel lifts
Mean
72.5
SD
16.8
Total
33

33
Weight
100.0%

100.0%

Mean Difference
IV, Fixed, 95% CI
-6.25 [-14.66 , 2.16]

-6.25 [-14.66 , 2.16]

Mean Difference
IV, Fixed, 95% CI


-100

-50

0

50

100


Favours heel lifts

Favours no treatment


Risk of Bias
A

+
B

?
C

+
D

+
E

?
F

?

Risk of bias legend

(A) Bias arising from the randomization process

(B) Bias due to deviations from intended interventions

(C) Bias due to missing outcome data

(D) Bias in measurement of the outcome

(E) Bias in selection of the reported result

(F) Overall bias


Analysis 9.2: Self reported treatment success in the medium term


Study or Subgroup
Wiegerinck 2016

Total
Test for overall effect: Z = 0.69 (P = 0.49)

Heterogeneity: Not applicable

Exercise
Mean
77.5
SD
12.02
Total
33

33

Heel lifts
Mean
75
SD
16.83
Total
33

33
Weight
100.0%

100.0%

Mean Difference
IV, Fixed, 95% CI
2.50 [-4.56 , 9.56]

2.50 [-4.56 , 9.56]

Mean Difference
IV, Fixed, 95% CI


-100

-50

0

50

100


Favours exercise

Favours heel lifts


Risk of Bias
A

+
B

?
C

+
D

+
E

?
F

?

Risk of bias legend

(A) Bias arising from the randomization process

(B) Bias due to deviations from intended interventions

(C) Bias due to missing outcome data

(D) Bias in measurement of the outcome

(E) Bias in selection of the reported result

(F) Overall bias


Analysis 9.3: Pain during activity in the short term


Study or Subgroup
Wiegerinck 2016

Total
Test for overall effect: Z = 1.16 (P = 0.25)

Heterogeneity: Not applicable

Exercise
Mean
-3.2
SD
2.5
Total
33

33

Heel lifts
Mean
-2.5
SD
2.4
Total
33

33
Weight
100.0%

100.0%

Mean Difference
IV, Fixed, 95% CI
-0.70 [-1.88 , 0.48]

-0.70 [-1.88 , 0.48]

Mean Difference
IV, Fixed, 95% CI


-10

-5

0

5

10


Favours exercise

Favours heel lifts


Risk of Bias
A

+
B

?
C

+
D

+
E

?
F

?

Risk of bias legend

(A) Bias arising from the randomization process

(B) Bias due to deviations from intended interventions

(C) Bias due to missing outcome data

(D) Bias in measurement of the outcome

(E) Bias in selection of the reported result

(F) Overall bias


Analysis 9.4: Pain during activity in the medium term


Study or Subgroup
Wiegerinck 2016

Total
Test for overall effect: Z = 0.69 (P = 0.49)

Heterogeneity: Not applicable

Exercise
Mean
-3.8
SD
3
Total
33

33

Heel lifts
Mean
-4.3
SD
2.9
Total
33

33
Weight
100.0%

100.0%

Mean Difference
IV, Fixed, 95% CI
0.50 [-0.92 , 1.92]

0.50 [-0.92 , 1.92]

Mean Difference
IV, Fixed, 95% CI


-10

-5

0

5

10


Favours exercise

Favours heel lifts


Risk of Bias
A

+
B

?
C

+
D

+
E

?
F

?

Risk of bias legend

(A) Bias arising from the randomization process

(B) Bias due to deviations from intended interventions

(C) Bias due to missing outcome data

(D) Bias in measurement of the outcome

(E) Bias in selection of the reported result

(F) Overall bias


Analysis 10.1: Pain during activity in the short term


Study or Subgroup
Perhamre 2012

Total
Test for overall effect: Z = 6.02 (P < 0.00001)

Heterogeneity: Not applicable

Foot orthoses, bracing, taping or straps
Mean
0.25
SD
0.14
Total
15

15

Usual care or no treatment
Mean
2.5
SD
1.44
Total
15

15
Weight
100.0%

100.0%

Mean Difference
IV, Fixed, 95% CI
-2.25 [-2.98 , -1.52]

-2.25 [-2.98 , -1.52]

Mean Difference
IV, Fixed, 95% CI


-100

-50

0

50

100


Favours foot orthoses

Favours no treatment


Risk of Bias
A

?
B

?
C

+
D

+
E

?
F

?

Risk of bias legend

(A) Bias arising from the randomization process

(B) Bias due to deviations from intended interventions

(C) Bias due to missing outcome data

(D) Bias in measurement of the outcome

(E) Bias in selection of the reported result

(F) Overall bias
