# Supplementary material for: Non‐surgical treatment for lower limb apophyseal injuries
Source: Cochrane Database Syst Rev. 2026 Jul 15;2026(7):CD015156. doi: 10.1002/14651858.CD015156.pub2 (PMC13370774; doi:10.1002/14651858.CD015156.pub2)
Supplement: Supplementary file 8 — Supplementary material 8 Consensus responses to the signalling questions for each domain for all extracted outcomes [file CD015156-SUP-08-other.html]

Consensus responses to the signalling questions for each domain for all extracted outcomes


# Supplementary material 8 to: Non-surgical treatment for lower limb apophyseal injuries

Williams CM, Krommes K, Paterson KL, Haines T, Caserta A, Thorborg K
  
https://doi.org/10.1002/14651858.CD015156.pub2

The material in this section has been supplied by the author(s) for publication under a Licence for Publication and the author(s) are solely responsible for the material. Cochrane has reviewed this material, but Cochrane has not copyedited, formatted or proofread. Cochrane accordingly gives no representations or warranties of any kind in relation to, and accepts no liability for any reliance on or use of, such material.

Back to top

# Consensus responses to the signalling questions for each domain for all extracted outcomes

|  |  |  |  |  |  |  |  |  |  |  |  |  |  |  |  |  |  |  |  |  |  |  |  |  |  |  |  |  |  |  |  |  |  |  |  |  |  |  |  |  |  |  |  |  |  |  |  |  |  |  |  |  |  |  |  |  |  |  |  |  |  |  |  |  |  |  |  |  |  |  |  |  |  |  |  |  |  |  |  |  |  |  |  |  |  |  |
| --- | --- | --- | --- | --- | --- | --- | --- | --- | --- | --- | --- | --- | --- | --- | --- | --- | --- | --- | --- | --- | --- | --- | --- | --- | --- | --- | --- | --- | --- | --- | --- | --- | --- | --- | --- | --- | --- | --- | --- | --- | --- | --- | --- | --- | --- | --- | --- | --- | --- | --- | --- | --- | --- | --- | --- | --- | --- | --- | --- | --- | --- | --- | --- | --- | --- | --- | --- | --- | --- | --- | --- | --- | --- | --- | --- | --- | --- | --- | --- | --- | --- | --- | --- | --- | --- | --- |
| Basic information | | | | | | | | | | | | | Domain 1. Randomization process | | | | | | | | | | Domain 2. Deviations from intended interventions | | | | | | | | | | | | | | | | | | Domain 3. Mising outcome data | | | | | | | | | | | | | Domain 4. Measurement of the outcome | | | | | | | | | | | | | | | Domain 5. Selection of the reported result | | | | | | | | | | | Domain 6. Overall Bias | | | |  |  |  |
| **Time** | **Unique ID** | **Assessor** | **Study ID** | **Reference** | **Experimental** | **Comparator** | **Outcome** | **Results** | **Aim** | **Effect of adhering to intervention?** | **Weight** | **Sources** | **1.1** | **1.2** | **Note for 1.1&1.2** | **1.3** | **Note for 1.3** | **1.0 Algorithm result** | **1.0 Assessor's Judgement** | **1.0 General note** | **1.0 Optional Question** | **1.0 Note for optional question** | **2.1** | **2.2** | **Note for 2.1&2.2** | **2.3** | **Note for 2.3** | **2.4** | **Note for 2.4** | **2.5** | **Note for 2.5** | **2.6** | **Note for 2.6** | **2.7** | **Note for 2.7** | **2.0 Algorithm result** | **2.0 Assessor's Judgement** | **2.0 General Notes** | **2.0 Optional Question** | **2.0 Note for optional question** | **3.1** | **Note for 3.1** | **3.2** | **Note for 3.2** | **3.3** | **Note for 3.3&3.4** | **3.4** | **Note for 3.4 (not use)** | **3.0 Algorithm result** | **3.0 Assessor's judgement** | **3.0 Gerenal notes** | **3.0 Optional Question** | **3.0 Note for optional question** | **4.1** | **Note for 4.1** | **4.2** | **Note for 4.2** | **4.3** | **Note for 4.3** | **4.4** | **Note for 4.4&4.5** | **4.5** | **Note for 4.5 (not use)** | **4.0 Algorithm result** | **4.0 Assessor's Judgement** | **4.0 General note** | **4.0 Optional Question** | **4.0 Note for optional question** | **5.1** | **Note for 5.1** | **5.2** | **Note for 5.2** | **5.3** | **Note for 5.3** | **5.0 Algorithm result** | **5.0 Assessor's Judgement** | **5.0 General note** | **5.0 Optional Question** | **5.0 Note for optional question** | **Algorithm's overall Judgement** | **Assessor's overall Judgement** | **6.0 General Note** | **6.0 Optional Question** | **6.0 Note for optional question** |  |  |
| 2025.03.31:10:30 | Alfaro-Santafa 2021 | AC/KP | Alfaro-Santafa 2021 | | Orthoses | Heel lift | Overall pain |  | assignment to intervention (the 'intention-to-treat' effect) | NA | 1 | Journal article(s); Non-commercial trial registry record (e.g. ClinicalTrials.gov record), published protocol, publications in repositories | Y | Y | Random allocation, concealed until participants were enrolled. | N | No differences visible in table with baseline characteristics | Low | Low | Random allocation, concealed until participants were enrolled. | | | Y | NI | Participants were aware of their assigned intervention during the trials.    No information was provieded about who delivered the intervention | N |  | NA |  | NA |  | Y |  | NA |  | Low | Low | Participants were aware of their assigned intervention during the trials.    No information was provieded about who delivered the intervention | | | Y | Data available for nearly all participants | PY |  | NA |  | NA |  | Low | Low | Data available for nearly all participants | | | PY | Limited information about the anchor for subjective VAS data. Outcomes described in protocol were described differently in publication. | N |  | NA |  | NA |  | NA |  | High | High | Limited information about the anchor for subjective VAS data. Outcomes described in protocol were described differently in publication. | | | N | Group randomisation and reporting results reported differently from the protocol to the publication. | PY | Y/PY | PY |  | High | High | Group randomisation and reporting results reported differently from the protocol to the publication. | | | High | High | Concerns about difference between reporting of results and protocol impacted the overall bias. | | | | |
| 2026/03/31 11.20 | James 2016 | AC/KP | James 2016 |  | Orthoses | Heel lift | Adverse events |  | assignment to intervention (the 'intention-to-treat' effect) | NA |  | Journal article(s); Trial protocol; Non-commercial trial registry record (e.g. ClinicalTrials.gov record) | Y | Y | Allocation blocks   PY/Y - ended with Y for 1.2 | PN | 1 group had more males | Low | Low | Concealed randomised allocation, with only minor differences in gender between one group. | | | Y | Y |  | N |  | NA |  | NA |  | PY |  | NA |  | Low | Low | Participants, carers and those delivering interventions were likely aware of intervention, and there were no non-protocol interventions, and followed the appropriate analysis plan specified in the protocol | | | Y | Nearly all data available | NA |  | NA |  | NA |  | Low | Low | Nearly all data available | |  | N |  | N |  | N |  | NA |  | NA |  | Low | Low | Appropriate outcome measures without outcome assessor aware of the interventions. | | | Y |  | N |  | N |  | Low | Low | Analysis protocol followed | |  | Low | Low | No other biases detected | |  |  |  |
| 2026/03/31 11.22 | James 2016 | AC/KP | James 2016 |  | Orthoses | Heel lift | Joint ROM |  | assignment to intervention (the 'intention-to-treat' effect) | NA |  | Journal article(s); Trial protocol; Non-commercial trial registry record (e.g. ClinicalTrials.gov record) | Y | Y | Allocation blocks   PY/Y - ended with Y for 1.2 | PN | 1 group had more males | Low | Low | Concealed randomised allocation, with only minor differences in gender between one group. | | | Y | Y |  | N |  | NA |  | NA |  | PY |  | NA |  | Low | Low | Participants, carers and those delivering interventions were likely aware of intervention, and there were no non-protocol interventions, and followed the appropriate analysis plan specified in the protocol | | | Y | Nearly all data available | NA |  | NA |  | NA |  | Low | Low | Nearly all data available | |  | N |  | N |  | N |  | NA |  | NA |  | Low | Low | Appropriate outcome measures without outcome assessor aware of the interventions. | | | Y |  | N |  | N |  | Low | Low | Analysis protocol followed | |  | Low | Low | No other biases detected | |  |  |  |
| 2026/03/31 11.28 | James 2016 | AC/KP | James 2016 |  | Orthoses | Heel lift | Overall pain |  | assignment to intervention (the 'intention-to-treat' effect) | NA |  | Journal article(s); Trial protocol; Non-commercial trial registry record (e.g. ClinicalTrials.gov record) | Y | Y | Allocation blocks   PY/Y - ended with Y for 1.2 | PN | 1 group had more males | Low | Low | Concealed randomised allocation, with only minor differences in gender between one group. | | | Y | Y |  | N |  | NA |  | NA |  | PY |  | NA |  | Low | Low | Participants, carers and those delivering interventions were likely aware of intervention, and there were no non-protocol interventions, and followed the appropriate analysis plan specified in the protocol | | | Y | Nearly all data available | NA |  | NA |  | NA |  | Low | Low | Nearly all data available | |  | N |  | N |  | N |  | NA |  | NA |  | Low | Low | Appropriate outcome measures with anchor questions to reduce subjectivity, without outcome assessor aware of the interventions. | | | Y |  | N |  | N |  | Low | Low | Analysis protocol followed | |  | Low | Low | No other biases detected | |  |  |  |
| 2026/03/31 11.30 | James 2016 | AC/KP | James 2016 |  | Orthoses | Heel lift | Physical Function |  | assignment to intervention (the 'intention-to-treat' effect) | NA |  | Journal article(s); Trial protocol; Non-commercial trial registry record (e.g. ClinicalTrials.gov record) | Y | Y | Allocation blocks   PY/Y - ended with Y for 1.2 | PN | 1 group had more males | Low | Low | Concealed randomised allocation, with only minor differences in gender between one group. | | | Y | Y |  | N |  | NA |  | NA |  | PY |  | NA |  | Low | Low | Participants, carers and those delivering interventions were likely aware of intervention, and there were no non-protocol interventions, and followed the appropriate analysis plan specified in the protocol | | | Y | Nearly all data available | NA |  | NA |  | NA |  | Low | Low | Nearly all data available | |  | N |  | N |  | N |  | NA |  | NA |  | Low | Low | Appropriate outcome measures with instructions to reduce subjectivity, without outcome assessor aware of the interventions. | | | Y |  | N |  | N |  | Low | Low | Analysis protocol followed | |  | Low | Low | No other biases detected | |  |  |  |
| 2026/03/31 10.54 | Kuyuci 2017 | AC/KP | Kuyuci 2017 |  | Taping | Placebo | Overall pain |  | assignment to intervention (the 'intention-to-treat' effect) | NA |  | Journal article(s) | NI | NI |  | NI | Not preported | Some concerns | Some concerns | No information about randomisation or baseline differences between groups. | | | NI | Y | Unclear if participants knew | NI |  | NA |  | NA |  | N |  | Y |  | High | High | Unclear if participants knew about their group allocation, no information to understand the effect of assignment, important because of sex based differences in apophysis closure. No information about if there was a deviation from usual practice. The analysis relied on multiple unadjusted statistical tests (including within-group comparisons) without clearly estimating between-group effects | | | PY |  | NA |  | NA |  | NA |  | Low | Low | Nearly all participant data were reported. | | | NI | Not enought information | N |  | NI | No enough | Y | No difference. | PN |  | Some concerns | Some concerns | Limited information about the anchor for subjective VAS data. | | | NI | No protocol | NI |  | NI |  | Some concerns | Some concerns | No pre-specified analysis plan identified. | | | High | High | No other bias identified | |  |  |  |
| 2026/03/31 10.58 | Kuyuci 2017 | AC/KP | Kuyuci 2017 |  | Taping | Placebo | Physical Function |  | assignment to intervention (the 'intention-to-treat' effect) | NA |  | Journal article(s) | NI | NI |  | NI | Not preported | Some concerns | Some concerns | No information about randomisation or baseline differences between groups. | | | NI | Y | Unclear if participants knew | NI |  | NA |  | NA |  | N |  | Y |  | High | High | Unclear if participants knew about their group allocation, no information to understand the effect of assignment, important because of sex based differences in apophysis closure. No information about if there was a deviation from usual practice. The analysis relied on multiple unadjusted statistical tests (including within-group comparisons) without clearly estimating between-group effects | | | PY |  | NA |  | NA |  | NA |  | Low | Low | Nearly all participant data were reported. | | | NI | Not enought information | N |  | NI | No enough | Y | No difference. | PN |  | Some concerns | Some concerns | No information on validity of tool for children as primarily an adult score | | | NI | No protocol | NI |  | NI |  | Some concerns | Some concerns | No pre-specified analysis plan identified. | | | High | High | No other bias identified | |  |  |  |
| 2026/03/31 12.02 | Nakase 2020 | AC/KP | Nakase 2020 |  | Pharmaceutical (Dextrose) | Placebo | Adverse events |  | assignment to intervention (the 'intention-to-treat' effect) | NA |  | Journal article(s) | NI | NI | Unclear | N |  | Some concerns | Some concerns | Randomisation process was not described. | | | N | N |  | NA |  | NA |  | NA |  | N |  | PY | Analysis based on t-tests at multiple points | Some concerns | Some concerns | Participants and people delivering intervention were blinded. No differences between groups. | | | PY |  | NA |  | NA |  | NA |  | Low | Low | Data available for nearly all participants. | | | PY |  | PN |  | NI | Didn't describe who was blinded. | Y |  | PN |  | Some concerns | Some concerns | It was unclear if the outcome assessors were aware of the intervention group. | | | NI | Unclear - unable to determine | PN |  | PN |  | Some concerns | Some concerns | No information on pre-specified analysis plan. | | | Some concerns | Some concerns | No other bias identified | |  |  |  |
| 2026/03/31 12.02 | Nakase 2020 | AC/KP | Nakase 2020 |  | Pharmaceutical (Dextrose) | Placebo | Pain during activity | | assignment to intervention (the 'intention-to-treat' effect) | NA |  | Journal article(s) | NI | NI | Unclear | N |  | Some concerns | Some concerns | Randomisation process was not described. | | | N | N |  | NA |  | NA |  | NA |  | N |  | PY | Analysis based on t-tests at multiple points | Some concerns | Some concerns | Participants and people delivering intervention were blinded. No differences between groups. | | | PY |  | NA |  | NA |  | NA |  | Low | Low | Data available for nearly all participants. | | | PN | ? Wrong outcome measure | PN |  | NI | Didn't describe who was blinded. | Y |  | PN |  | Some concerns | Some concerns | It was unclear if the method of measuring outcome was appropriate for the knee as only the VISA was described. It was unclear if the outcome assessors were aware of the intervention group. | | | NI | Unclear - unable to determine | PN |  | PN |  | Some concerns | Some concerns | No information on pre-specified analysis plan. | | | Some concerns | Some concerns | No other bias identified | |  |  |  |
| 2026/03/31 11.52 | Perhamre 2011 | AC/KP | Peramre 2011 |  | Foot orthoses | Heel lift | Pain during activity | | assignment to intervention (the 'intention-to-treat' effect) | NA |  | Journal article(s) | Y | NI |  | N |  | Some concerns | Some concerns | Randomized into two groups with tickets concealed in a box. No stratification was made. | | | NI | PY |  | NI |  | NA |  | NA |  | PY |  | NA |  | Some concerns | Some concerns | No information on if participants were aware of assigned intervention | | | PY |  | NA |  | NA |  | NA |  | Low | Low | Outcome data available. | |  | N |  | N |  | NI |  | PY | Based on how the trial was reported and next phase | PN |  | Some concerns | Some concerns | No information about blinding of assessor which could have impacted outcome. | | | NI |  | NI |  | NI |  | Some concerns | Some concerns | No protocol or pre-specified analysis plan | | | Some concerns | Some concerns | No protocol or pre-analysis plan. | |  |  |  |
| 2026/03/31 11.40 | Perhamre 2012 | AC/KP | Perhamre 2012 |  | Foot orthoses | Usual care | Pain during activity | | assignment to intervention (the 'intention-to-treat' effect) | NA |  | Journal article(s) | Y | NI | Randomised but no infomation | N |  | Some concerns | Some concerns | Randomised but no information about how or if there was allocation concealment | | | Y | PY | Unclear if they knew but based on writing. | NI |  | NA |  | NA |  | PY | Appropriate | NA |  | Some concerns | Some concerns | Participants and carers knew the allocation. Unclear is assessors knew allocation. | | | Y |  |  |  |  |  |  |  | Low | Low | All outcomes for randomised participants were analysed. | | | N |  | PN |  | PN |  | NA |  | NA |  | Low | Low | Outcome mesaure appropriate and self reported | | | NI | No protocol | NI |  | NI |  | Some concerns | Some concerns | No protocol or pre-analysis plan. | |  | Some concerns | Some concerns | No protocol or pre-analysis plan. | |  |  |  |
| 2026/03/31 11.21 | Reesman 2024 | AC/KP | Reesman 2024 |  | Pharmaceutical (Dexamethasone) | Placebo & Usual care | Adverse events |  | assignment to intervention (the 'intention-to-treat' effect) | NA |  | Non-commercial trial registry record (e.g. ClinicalTrials.gov record) | NI | NI |  | NI |  | Some concerns | Some concerns | No information about randomisation process. | | | PY | NI |  | NI |  | NA |  | NA |  | NI |  | NI |  | High | High | No information about deviations due to trial ending early | | | N |  | N |  | NI |  | NI |  | High | High | Trial ended early, missing outcome data. | | | PN |  | PN |  | NI |  | NI |  | PN |  | Some concerns | Some concerns | Trial ended early, limited information about how outcomes were collected | | | N |  | NI |  | NI |  | Some concerns | Some concerns | No protocol published. | |  | High | High | No other bias identified | |  |  |  |
| 2026/03/31 11.25 | Reesman 2024 | AC/KP | Reesman 2024 |  | Pharmaceutical (Dexamethasone) | Placebo & Usual care | Overall pain |  | assignment to intervention (the 'intention-to-treat' effect) | NA |  | Non-commercial trial registry record (e.g. ClinicalTrials.gov record) | NI | NI |  | NI |  | Some concerns | Some concerns | No information about randomisation process. | | | PY | NI |  | NI |  | NA |  | NA |  | NI |  | NI |  | High | High | No information about deviations due to trial ending early | | | N |  | N |  | NI |  | NI |  | High | High | Trial ended early, missing outcome data. | | | PN |  | PN |  | NI |  | NI |  | PN |  | High | High | Trial ended early, limited information about how outcomes were collected and the outcome is subjective | | | N |  | NI |  | NI |  | Some concerns | Some concerns | No protocol published. | |  | High | High | No other bias identified | |  |  |  |
| 2026/03/31 11.27 | Reesman 2024 | AC/KP | Reesman 2024 |  | Pharmaceutical (Dexamethasone) | Placebo & Usual care | Physical Function |  | assignment to intervention (the 'intention-to-treat' effect) | NA |  | Non-commercial trial registry record (e.g. ClinicalTrials.gov record) | NI | NI |  | NI |  | Some concerns | Some concerns | No information about randomisation process. | | | PY | NI |  | NI |  | NA |  | NA |  | NI |  | NI |  | High | High | No information about deviations due to trial ending early | | | N |  | N |  | NI |  | NI |  | High | High | Trial ended early, missing outcome data. | | | PN |  | PN |  | NI |  | NI |  | PN |  | High | High | Trial ended early, limited information about how outcomes were collected and the outcome is subjective | | | N |  | NI |  | NI |  | Some concerns | Some concerns | No protocol published. | |  | High | High | No other bias identified | |  |  |  |
| 2026/03/31 11.27 | Reesman 2024 | AC/KP | Reesman 2024 |  | Pharmaceutical (Dexamethasone) | Placebo & Usual care | Sport Participation |  | assignment to intervention (the 'intention-to-treat' effect) | NA |  | Non-commercial trial registry record (e.g. ClinicalTrials.gov record) | NI | NI |  | NI |  | Some concerns | Some concerns | No information about randomisation process. | | | PY | NI |  | NI |  | NA |  | NA |  | NI |  | NI |  | High | High | No information about deviations due to trial ending early | | | N |  | N |  | NI |  | NI |  | High | High | Trial ended early, missing outcome data. | | | PN |  | PN |  | NI |  | NI |  | PN |  | High | High | Trial ended early, limited information about how outcomes were collected and the outcome is subjective | | | N |  | NI |  | NI |  | Some concerns | Some concerns | No protocol published. | |  | High | High | No other bias identified | |  |  |  |
| 2026/03/31 12.14 | Sweeney 2023 | AC/KP | Sweeney 2023 |  | Brace | Cushion | Adverse events |  | assignment to intervention (the 'intention-to-treat' effect) | NA |  | Journal article(s); Trial protocol | Y | PY |  | N |  | Low | Low | Randomized participants into each group at enrollment using a 1:1 block randomization scheme (block size 4), concealed until assigned, with no group differences. | | | Y | Y |  | N |  | NA |  | NA |  | Y |  | NA |  | Low | Low | Participants and carers were aware of group assignment, without deviations due to trial context and appropriate analysis of adverse event data | | | N |  | N |  | Y |  | PN |  | Some concerns | Some concerns | Missing data from 11 participants, 16 participants in analysis, potential that missingness impacted outcomes. | | | N |  | PY |  | NA |  | NA |  | NA |  | Low | Low | Data self reported monthly | |  | N |  | N |  | PY | Used different analysis to the protocol | High | High | Analysis used Mann-Whitney U test and Hedges g to determine the magnitude of the effect between groups and this was different to the analysis reported within the protocol. | | | High | High | No other bias identified | |  |  |  |
| 2026/03/31 12.16 | Sweeney 2023 | AC/KP | Sweeney 2023 |  | Brace | Cushion | Pain during activity | | assignment to intervention (the 'intention-to-treat' effect) | NA |  | Journal article(s); Trial protocol | Y | PY |  | N |  | Low | Low | Randomized participants into each group at enrollment using a 1:1 block randomization scheme (block size 4), concealed until assigned, with no group differences. | | | Y | Y |  | N |  | NA |  | NA |  | PN |  | PN |  | Some concerns | Some concerns | Participants and carers were aware of group assignment, without deviations due to trial context | | | N |  | N |  | Y |  | PN |  | Some concerns | Some concerns | Missing data from 11 participants, 16 participants in analysis, potential that missingness impacted outcomes. | | | N |  | PY |  | NA |  | NA |  | NA |  | Low | Low | Data self reported monthly | |  | N |  | N |  | PY | Used different analysis to the protocol | High | High | Analysis used Mann-Whitney U test and Hedges g to determine the magnitude of the effect between groups and this was different to the analysis reported within the protocol. | | | High | High | No other bias identified | |  |  |  |
| 2026/03/31 12.17 | Sweeney 2023 | AC/KP | Sweeney 2023 |  | Brace | Cushion | Physical Function |  | assignment to intervention (the 'intention-to-treat' effect) | NA |  | Journal article(s); Trial protocol | Y | PY |  | N |  | Low | Low | Randomized participants into each group at enrollment using a 1:1 block randomization scheme (block size 4), concealed until assigned, with no group differences. | | | Y | Y |  | N |  | NA |  | NA |  | PN |  | PN |  | Some concerns | Some concerns | Participants and carers were aware of group assignment, without deviations due to trial context | | | N |  | N |  | Y |  | PN |  | Some concerns | Some concerns | Missing data from 11 participants, 16 participants in analysis, potential that missingness impacted outcomes. | | | N |  | PY |  | NA |  | NA |  | NA |  | Low | Low | Data self reported monthly | |  | N |  | N |  | PY | Used different analysis to the protocol | High | High | Analysis used Mann-Whitney U test and Hedges g to determine the magnitude of the effect between groups and this was different to the analysis reported within the protocol. | | | High | High | No other bias identified | |  |  |  |
| 2026/03/31 12.21 | Topol 2011 | AC/KP | Topol 2011 |  | Dextrose | Placebo/usual care | Pain during activity | | assignment to intervention (the 'intention-to-treat' effect) | NA |  | Journal article(s); Trial protocol | Y | Y |  | NI | No table of participatnts | Low | Low | Random numbers table was used for assignment to supervised usual care or to an injection solution group blinded to the subject, guardian, and the treating/evaluating physician. | | | N | N |  | NA |  | NA |  | NA |  | PY |  | NA |  | Low | Low | All participant data included. | |  | Y |  | NA |  | NA |  | NA |  | Low | Low | Outcome measurement described | |  | N |  | PN |  | N |  | NA |  | NA |  | Low | Low | Outcome measurement described | |  | NI | Protocol analysis plan not available. | N |  | NI | Unclear due to analysis plan | Some concerns | Some concerns | Protocol analysis plan not available. | |  | Some concerns | Some concerns | No other bias identified | |  |  |  |
| 2026/03/31 12.29 | Wiegnerinck 2016 | AC/KP | Wiegnerinck 2016 |  | Ecercise | Heel lift / Usual care | Adverse events |  | assignment to intervention (the 'intention-to-treat' effect) | NA |  | Journal article(s); Trial protocol | Y | Y |  | PN | Different in males. | Low | Low | Randomisation described with minimial between group differences. | | | Y | Y |  | NI |  | NA |  | NA |  | PY |  | NA |  | Some concerns | Some concerns | Participants and carers aware of group allocation. | | | Y |  | NA |  | NA |  | NA |  | Low | Low | Limited missing outcome data. | |  | N |  | PN |  | N |  | NA |  | NA |  | Low | Low | No bias identified in outcome measurement | | | NI | Unclear - no known anlaysis plan | NI |  | PN |  | Some concerns | Some concerns | No identified pre-analysis plan | |  | Some concerns | Some concerns | No other bias identified | |  |  |  |
| 2026/03/31 12.33 | Wiegnerinck 2016 | AC/KP | Wiegnerinck 2016 |  | Ecercise | Heel lift / Usual care | Pain during activity | | assignment to intervention (the 'intention-to-treat' effect) | NA |  | Journal article(s); Trial protocol | Y | Y |  | PN | Different in males. | Low | Low | Randomisation described with minimial between group differences. | | | Y | Y |  | NI |  | NA |  | NA |  | PY |  | NA |  | Some concerns | Some concerns | Participants and carers aware of group allocation. | | | Y |  | NA |  | NA |  | NA |  | Low | Low | Limited missing outcome data. | |  | N |  | PN |  | N |  | NA |  | NA |  | Low | Low | No bias identified in outcome measurement | | | NI | Unclear - no known anlaysis plan | NI |  | PN |  | Some concerns | Some concerns | No identified pre-analysis plan | |  | Some concerns | Some concerns | No other bias identified | |  |  |  |
| 2026/03/31 12.36 | Wiegnerinck 2016 | AC/KP | Wiegnerinck 2016 |  | Ecercise | Heel lift / Usual care | Treatment success |  | assignment to intervention (the 'intention-to-treat' effect) | NA |  | Journal article(s); Trial protocol | Y | Y |  | PN | Different in males. | Low | Low | Randomisation described with minimial between group differences. | | | Y | Y |  | NI |  | NA |  | NA |  | PY |  | NA |  | Some concerns | Some concerns | Participants and carers aware of group allocation. | | | Y |  | NA |  | NA |  | NA |  | Low | Low | Limited missing outcome data. | |  | N |  | PN |  | N |  | NA |  | NA |  | Low | Low | No bias identified in outcome measurement | | | NI | Unclear - no known anlaysis plan | NI |  | PN |  | Some concerns | Some concerns | No identified pre-analysis plan | |  | Some concerns | Some concerns | No other bias identified | |  |  |  |
