# Supplementary material for: Non‐surgical treatment for lower limb apophyseal injuries
Source: Cochrane Database Syst Rev. 2026 Jul 15;2026(7):CD015156. doi: 10.1002/14651858.CD015156.pub2 (PMC13370774; doi:10.1002/14651858.CD015156.pub2)
Supplement: Supplementary file 9 — Supplementary material 9 Supplementary summary of findings: Pharmaceutical intervention compared to a placebo for children with traction apophysitis of the tibial tubercle for all outcomes [file CD015156-SUP-09-other.html]

Supplementary summary of findings: Pharmaceutical intervention compared to a placebo for children with traction apophysitis of the tibial tubercle for all outcomes


# Supplementary material 9 to: Non-surgical treatment for lower limb apophyseal injuries

Williams CM, Krommes K, Paterson KL, Haines T, Caserta A, Thorborg K
  
https://doi.org/10.1002/14651858.CD015156.pub2

The material in this section has been supplied by the author(s) for publication under a Licence for Publication and the author(s) are solely responsible for the material. Cochrane has reviewed this material, but Cochrane has not copyedited, formatted or proofread. Cochrane accordingly gives no representations or warranties of any kind in relation to, and accepts no liability for any reliance on or use of, such material.

Back to top

# Supplementary summary of findings: Pharmaceutical intervention compared to a placebo for children with traction apophysitis of the tibial tubercle for all outcomes

|  |  |  |  |  |  |  |
| --- | --- | --- | --- | --- | --- | --- |
| **Summary of findings:** | | | | | | |
| **A pharmaceutical intervention compared to placebo for children with traction apophysitis of the tibial tubercle** | | | | | | |
| **Patient or population:**  children with traction apophysitis of the tibial tubercle  **Setting:** Tertiary Care  **Intervention:**  a pharmaceutical intervention  **Comparison:**  placebo | | | | | | |
| Outcomes | **Anticipated absolute effects\*** (95% CI) | | Relative effect (95% CI) | № of participants (studies) | Certainty of the evidence (GRADE) | Comments |
|  | **Risk with placebo** | **Risk with a pharmaceutical intervention** |  |  |  |  |
| Overall pain assessed with: VAS (Lower = less pain) Scale from: 0 to 10 follow-up: 8 weeks | The mean overall pain was **1.85** points | MD **0.52 points lower**  (1.24 lower to 0.2 higher) | - | 23 (1 RCT) | ⨁◯◯◯ Very low a,b,c | The evidence is very uncertain about the effect of a pharmaceutical intervention on overall pain in the short term. |
| Physical function assessed with: LEFS (Higher = greater function) Scale from: 0 to 100 follow-up: 8 weeks | The mean physical function was **86.42** points | MD **1.76 points lower**  (16.08 lower to 12.56 higher) | - | 19 (1 RCT) | ⨁◯◯◯ Very lowa,b,c | The evidence is very uncertain about the effect of a pharmaceutical intervention on physical function in the short term. |
| Participation in sports or physical activity assessed with: Days to return to sport (Lower = quicker) Scale from: 0 to 56 follow-up: 8 weeks | The mean participation in sport was **30.2** days | MD **7.9 days higher**  (0.41 lower to 16.21 higher) | - | 16 (1 RCT) | ⨁◯◯◯ Very lowa,b,c | The evidence is very uncertain about the effect of a pharmaceutical intervention on participation in sport in the short term. |
| Withdrawals due to adverse events - not measured |  | |  | - | - | There were 3 withdrawals due to not wanting another injection however these were not reported as a result of an adverse event |
| Adverse events assessed with: Count follow-up: range 8 weeks to 12 weeks | 278 per 1000 | **364 per 1000**  (244 to 544) | **RR 1.31**  (0.88 to 1.96) | 74 (2 RCTs) | ⨁◯◯◯ Very lowa,b,c | The evidence is very uncertain about the adverse effects of pharmaceutical interventions. |
| Treatment success - not measured |  | |  | - | - |  |
| Change in pain during activity (Pharmaceuticals) assessed with: VISA (Higher = less pain)  Scale from: 0 to 100  follow-up: 1 months | The mean overallin pain during activity was 27.4 points | MD 4.3 points higher  (8.46 lower to 17.06 higher) | - | 43 (1 RCT) | ⊕⊝⊝⊝  Very low d,e | The evidence is very uncertain about the effect of a pharmaceutical intervention on the change of pain during activity in the short term |
| Pain during an activity assessed with: VISA (Reversed where lower = less pain) and NPPS (Lower = less pain) Scale from: 0 to 100 follow-up: mean 3 months | - | SMD **2.36 higher**  (29.63 lower to 34.35 higher) | - | 86 (2 RCTs) | ⨁⨁◯◯ Lowd,e | A pharmaceutical may result in little to no difference in pain during an activity in the medium term. |
| Pain during an activity assessed with: NPPS (Lower = less pain) Scale from: 0 to 7 follow-up: 12 months | The mean pain during an activity was **- 1.2** points | MD **1 points lower**  (1.87 lower to 0.13 lower) | - | 34 (1 RCT) | ⊕⊝⊝⊝  Very low f,g | A pharmaceutical intervention may result in little to no difference in pain during an activity in the long term. |
| Active range of motion - not measured |  |  |  |  |  |  |
| Quality of life - not measured |  |  |  |  |  |  |
| \***The risk in the intervention group** (and its 95% confidence interval) is based on the assumed risk in the comparison group and the **relative effect** of the intervention (and its 95% CI).    **CI:** confidence interval; **MD:** mean difference; **RR:** risk ratio; **SMD:** standardised mean difference | | | | | | |
| **GRADE Working Group grades of evidence**   **High certainty:** we are very confident that the true effect lies close to that of the estimate of the effect.  **Moderate certainty:** we are moderately confident in the effect estimate: the true effect is likely to be close to the estimate of the effect, but there is a possibility that it is substantially different.  **Low certainty:** our confidence in the effect estimate is limited: the true effect may be substantially different from the estimate of the effect.  **Very low certainty:** we have very little confidence in the effect estimate: the true effect is likely to be substantially different from the estimate of effect. | | | | | | |

#### Explanations

a We downgraded twice for risk of bias as single study had a high risk of bias  
b We downgraded twice for imprecision as as sample size was not reached and trial ceased early  
c We downgraded for publication bias as only limited results were available from online trial registry without information on adherence to protocol  
d We downgraded once for risk of bias as it was unclear how participants were randomised or how allocation was concealed in one trial  
e We downgraded once for imprecision due large confidence interval  
f We downgraded once for risk of bias due to some concerns with trial blinding  
g We downgraded twice for imprecision due to very small participant numbers
