# Supplementary material for: Non‐surgical treatment for lower limb apophyseal injuries
Source: Cochrane Database Syst Rev. 2026 Jul 15;2026(7):CD015156. doi: 10.1002/14651858.CD015156.pub2 (PMC13370774; doi:10.1002/14651858.CD015156.pub2)
Supplement: Supplementary file 10 — Supplementary material 10 Supplementary summary of findings: pharmaceutical intervention compared to usual care for children with traction apophysitis of the tibial tubercle for all outcomes [file CD015156-SUP-10-other.html]

Supplementary summary of findings: pharmaceutical intervention compared to usual care for children with traction apophysitis of the tibial tubercle for all outcomes


# Supplementary material 10 to: Non-surgical treatment for lower limb apophyseal injuries

Williams CM, Krommes K, Paterson KL, Haines T, Caserta A, Thorborg K
  
https://doi.org/10.1002/14651858.CD015156.pub2

The material in this section has been supplied by the author(s) for publication under a Licence for Publication and the author(s) are solely responsible for the material. Cochrane has reviewed this material, but Cochrane has not copyedited, formatted or proofread. Cochrane accordingly gives no representations or warranties of any kind in relation to, and accepts no liability for any reliance on or use of, such material.

Back to top

# Supplementary summary of findings: pharmaceutical intervention compared to usual care for children with traction apophysitis of the tibial tubercle for all outcomes

|  |  |  |  |  |  |  |
| --- | --- | --- | --- | --- | --- | --- |
| **Summary of findings for all outcomes:** | | | | | | |
| **A pharmaceutical intervention compared to usual care for children with traction apophysitis of the tibial tubercle** | | | | | | |
| **Patient or population:**  children with traction apophysitis of the tibial tubercle  **Setting:**  Tertiarycare  **Intervention:**  a pharmaceutical intervention  **Comparison:**  usual care | | | | | | |
| Outcomes | **Anticipated absolute effects\*** (95% CI) | | Relative effect (95% CI) | № of participants (studies) | Certainty of the evidence (GRADE) | Comments |
|  | **Risk with usual care** | **Risk with a pharmaceutical intervention** |  |  |  |  |
| Overall pain  assessed with: VAS (Lower = less pain) Scale from: 0 to 10 follow-up: 8 weeks | The mean overall pain was **2.13** points | MD **0.8 points lower**  (1.73 lower to 0.13 higher) | - | 21 (1 RCT) | ⨁◯◯◯ Very lowa,b,c | The evidence is very uncertain about the effect of a dexamethasone on overall pain in the short term. |
| Physical function assessed with: LEFS (Higher = greater function) Scale from: 0 to 100 follow-up: 8 weeks | The mean physical function was **81.98** points | MD **2.68 points higher**  (17.56 lower to 22.92 higher) | - | 16 (1 RCT) | ⨁◯◯◯ Very lowa,b,c | The evidence is very uncertain about the effect of a dexamethasone on physical function in the short term |
| Participation in sport or physical activity assessed with: Days to return to sport (Lower = quicker) Scale from: 0 to 56 follow-up: 8 weeks | The mean participation in sport was **37.25** days | MD **0.85 days higher**  (7.13 lower to 8.83 higher) | - | 11 (1 RCT) | ⨁◯◯◯ Very lowa,b,c | The evidence is very uncertain about the effect of a dexamethasone on participation in sport in the short term. |
| Treatment success - not measured |  | |  | - | - |  |
| Withdrawals due to adverse events - not measured |  | |  | - | - |  |
| Adverse events assessed with: Count follow-up: 8 weeks | 643 per 1000 | **874 per 1000**  (566 to 1000) | **RR 1.36**  (0.88 to 2.10) | 30 (1 RCT) | ⨁◯◯◯ Very lowa,b,c | The evidence is very uncertain about the adverse effects of dexamethasone. |
| Pain during an activity assessed with: NPPS (Lower = less pain) Scale from: 0 to 7 follow-up: 3 months | The mean pain during an activity was **3.1** points | MD **2.4 points lower**  (3.24 lower to 1.56 lower) | - | 43 (1 RCT) | ⨁◯◯◯ Very lowd,e | The evidence is very uncertain about the effect of a dextrose injections on pain during an activity in the medium term. |
| Pain during an activity assessed with: NPPS (Lower = less pain) Scale from: 0 to 7 follow-up: 12 months | The mean pain during an activity was **2.5** points | MD **2.3 points lower**  (3.14 lower to 1.46 lower) | - | 35 (1 RCT) | ⨁◯◯◯ Very lowd,e | The evidence is very uncertain about the effect of a dextrose injections on pain during an activity in the long term. |
| Active range of motion - not measured |  | |  | - | - |  |
| Quality of life - not measured |  | |  | - | - |  |
| \***The risk in the intervention group** (and its 95% confidence interval) is based on the assumed risk in the comparison group and the **relative effect** of the intervention (and its 95% CI).    **CI:** confidence interval; **MD:** mean difference; **RR:** risk ratio | | | | | | |
| **GRADE Working Group grades of evidence**   **High certainty:** we are very confident that the true effect lies close to that of the estimate of the effect.  **Moderate certainty:** we are moderately confident in the effect estimate: the true effect is likely to be close to the estimate of the effect, but there is a possibility that it is substantially different.  **Low certainty:** our confidence in the effect estimate is limited: the true effect may be substantially different from the estimate of the effect.  **Very low certainty:** we have very little confidence in the effect estimate: the true effect is likely to be substantially different from the estimate of effect. | | | | | | |

#### Explanations

a We downgraded twice for risk of bias as single study had a high risk of bias  
b We downgraded twice for imprecision as as sample size was not reached and trial ceased early  
c We downgraded for publication bias as only limited results were available from online trial registry without information on adherence to protocol  
d We downgraded once for risk of bias due to some concerns with trial blinding  
e We downgraded twice for imprecision due to very small participant numbers
