# Supplementary material for: Non‐surgical treatment for lower limb apophyseal injuries
Source: Cochrane Database Syst Rev. 2026 Jul 15;2026(7):CD015156. doi: 10.1002/14651858.CD015156.pub2 (PMC13370774; doi:10.1002/14651858.CD015156.pub2)
Supplement: Supplementary file 11 — Supplementary material 11 Supplementary findings tables: Taping compared to placebo for children with calcaneal apophysitis for all outcomes [file CD015156-SUP-11-other.html]

Supplementary findings tables: Taping compared to placebo for children with calcaneal apophysitis for all outcomes


# Supplementary material 11 to: Non-surgical treatment for lower limb apophyseal injuries

Williams CM, Krommes K, Paterson KL, Haines T, Caserta A, Thorborg K
  
https://doi.org/10.1002/14651858.CD015156.pub2

The material in this section has been supplied by the author(s) for publication under a Licence for Publication and the author(s) are solely responsible for the material. Cochrane has reviewed this material, but Cochrane has not copyedited, formatted or proofread. Cochrane accordingly gives no representations or warranties of any kind in relation to, and accepts no liability for any reliance on or use of, such material.

Back to top

# Supplementary findings tables: Taping compared to placebo for children with calcaneal apophysitis for all outcomes

|  |  |  |  |  |  |  |
| --- | --- | --- | --- | --- | --- | --- |
| **Summary of findings for all outcomes:** | | | | | | |
| **Taping compared to placebo for children with calcaneal apophysitis** | | | | | | |
| **Patient or population:**  children with calcaneal apophysitis  **Setting:**  Tertiary care  **Intervention:**  taping  **Comparison:**  placebo | | | | | | |
| Outcomes | **Anticipated absolute effects\*** (95% CI) | | Relative effect (95% CI) | № of participants (studies) | Certainty of the evidence (GRADE) | Comments |
|  | **Risk with placebo** | **Risk with taping** |  |  |  |  |
| Overall pain assessed with: VAS (Lower = less pain) Scale from: 0 to 10 follow-up: 4 weeks | The mean overall pain was **5.4** points | MD **0.1 cm higher**  (1.25 lower to 1.45 higher) | - | 22 (1 RCT) | ⨁◯◯◯ Very lowa,b | The evidence is very uncertain about the effect of taping on overall pain in the short term. |
| Overall pain  assessed with: VAS (Lower = less pain) Scale from: 0 to 10 follow-up: 3 months | The mean overall pain was **1.5** points | MD **1 cm lower**  (1.77 lower to 0.23 lower) | - | 22 (1 RCT) | ⨁◯◯◯ Very lowa,b | The evidence is very uncertain about the effect of taping on overall pain in the medium term. |
| Physical function assessed with: AOFAS (Higher = greater function Scale from: 0 to 100 follow-up: 4 weeks | The mean physical function was **77.4** points | MD **6.1 points higher**  (0.08 lower to 12.28 higher) | - | 22 (1 RCT) | ⨁◯◯◯ Very lowa,b | The evidence is very uncertain about the effect of taping on physical function in the short term. |
| Physical function assessed with: AOFAS (Higher = greater function) Scale from: 0 to 100 follow-up: 3 months | The mean physical function was **87.1** points | MD **2.3 points higher**  (0.07 lower to 4.67 higher) | - | 22 (1 RCT) | ⨁◯◯◯ Very lowa,b | The evidence is very uncertain about the effect of taping on physical function in the medium term. |
| Participation in sport or physical activity - not measured |  | |  | - | - |  |
| Withdrawals due to adverse events - not measured |  | |  | - | - |  |
| Total adverse events - not measured |  | |  | - | - |  |
| Pain during an activity - not measured |  | |  | - | - |  |
| Active range of motion - not measured |  | |  | - | - |  |
| Quality of life - not measured |  | |  | - | - |  |
| \***The risk in the intervention group** (and its 95% confidence interval) is based on the assumed risk in the comparison group and the **relative effect** of the intervention (and its 95% CI).    **CI:** confidence interval; **MD:** mean difference | | | | | | |
| **GRADE Working Group grades of evidence**   **High certainty:** we are very confident that the true effect lies close to that of the estimate of the effect.  **Moderate certainty:** we are moderately confident in the effect estimate: the true effect is likely to be close to the estimate of the effect, but there is a possibility that it is substantially different.  **Low certainty:** our confidence in the effect estimate is limited: the true effect may be substantially different from the estimate of the effect.  **Very low certainty:** we have very little confidence in the effect estimate: the true effect is likely to be substantially different from the estimate of effect. | | | | | | |

#### Explanations

aWe downgraded twice for risk of bias as single study had a high risk of bias

bWe downgraded twice for imprecision due to very small participant numbers
