# Supplementary material for: Non‐surgical treatment for lower limb apophyseal injuries
Source: Cochrane Database Syst Rev. 2026 Jul 15;2026(7):CD015156. doi: 10.1002/14651858.CD015156.pub2 (PMC13370774; doi:10.1002/14651858.CD015156.pub2)
Supplement: Supplementary file 12 — Supplementary material 12 Supplementary summary of findings: Foot orthoses compared to heel lifts for children with calcaneal apophysitis for all outcomes [file CD015156-SUP-12-other.html]

Supplementary summary of findings: Foot orthoses compared to heel lifts for children with calcaneal apophysitis for all outcomes


# Supplementary material 12 to: Non-surgical treatment for lower limb apophyseal injuries

Williams CM, Krommes K, Paterson KL, Haines T, Caserta A, Thorborg K
  
https://doi.org/10.1002/14651858.CD015156.pub2

The material in this section has been supplied by the author(s) for publication under a Licence for Publication and the author(s) are solely responsible for the material. Cochrane has reviewed this material, but Cochrane has not copyedited, formatted or proofread. Cochrane accordingly gives no representations or warranties of any kind in relation to, and accepts no liability for any reliance on or use of, such material.

Back to top

# Supplementary summary of findings: Foot orthoses compared to heel lifts for children with calcaneal apophysitis for all outcomes

|  |  |  |  |  |  |  |
| --- | --- | --- | --- | --- | --- | --- |
| **Summary of findings:** | | | | | | |
| **Foot orthoses compared to heel lifts for children with calcaneal apophysitis** | | | | | | |
| **Patient or population:**  children with calcaneal apophysitis  **Setting:**  Tertiary care  **Intervention:**  foot orthoses  **Comparison:**  heel lifts | | | | | | |
| Outcomes | **Anticipated absolute effects\*** (95% CI) | | Relative effect (95% CI) | № of participants (studies) | Certainty of the evidence (GRADE) | Comments |
|  | **Risk with heel lifts** | **Risk with foot orthoses** |  |  |  |  |
| Overall pain  assessed with: Faces pain scale (Lower = less pain) Scale from: 0 to 6 follow-up: 4 months | The mean overall pain was **2.9** points | MD **0 points**  (0.44 lower to 0.44 higher) |  | 124 (1 RCT) | ⨁⨁⨁◯ Moderatea | Foot orthoses likely result in little to no difference in overall pain in the short term compared to heel lifts |
| Overall pain  assessed with: VAS (Lower = less pain) Scale from: 0 to 100 follow-up: 3 months | The mean overall pain was **67.3** mm | MD **55.7 mm lower**  (60.97 lower to 50.43 lower) | - | 208 (1 RCT) | ⨁◯◯◯ Very lowb,c | Foot orthoses may result in a reduction in overall pain in the medium term compared to heel lifts. |
| Physical function assessed with: OAFQ-C (Physical) (Higher = better function) Scale from: 0 to 100 follow-up: 4 | The mean physical function was **64.4** points | MD **1.3 points lower**  (7.58 lower to 4.98 higher) | - | 124 (1 RCT) | ⨁⨁⨁◯ Moderatea | Foot orthoses likely result in little to no difference in physical function in the short term compared to heel lifts |
| Physical function assessed with: OAFQ-C (Physical) (Higher = better function) Scale from: 0 to 100 follow-up: 6 months | The mean physical function was **82.0** points | MD **7.8 points lower**  (14.22 lower to 1.38 lower) | - | 106 (1 RCT) | ⨁⨁⨁◯ Moderatea | Foot orthoses likely result in a slight reduction in physical function in the medium term compared to heel lifts. |
| Physical function assessed with: OAFQ-C (Physical) (Higher = better function) Scale from: 0 to 100 follow-up: 12 months | The mean physical function was **83.8** points | MD **4.3 points lower**  (10.68 lower to 2.08 higher) | - | 101 (1 RCT) | ⨁⨁⨁◯ Moderatea | Foot orthoses likely result in little to no difference in physical function in the long term compared to heel lifts |
| Participation in sport or physical activity - not measured |  | |  | - | - |  |
| Withdrawals due to adverse events - not measured |  | |  |  |  |  |
| Adverse events  assessed with: Count follow-up: 12 months | Not pooled as there were no reported adverse events in either group. | |  | 101 (1 RCT) | ⨁⨁⨁◯ Moderatea | Foot orthoses likely do not increase adverse events compared to heel lifts in the long term. |
| Treatment success - not measured |  | |  |  |  |  |
| Pain during activity assessed with: Borg-10 (Lower = less pain) Scale from: 0 to 10 follow-up: 4 weeks | The mean pain during activity in the short term was **3.17** points | MD **1 points lower**  (2.29 lower to 0.29 higher) | - | 44 (1 RCT) | ⨁◯◯◯ Very lowd,e | The evidence is very uncertain about the effect of foot orthoses on pain during activity in the short term compared to heel lifts. |
| Joint range of motion assessed with: Weight bearing lunge (Higher = greater range) Scale from: 0 to 50 follow-up: 4 weeks | The mean joint range of motion was **30.9** degrees | MD **0.7 degrees higher**  (0.98 lower to 2.38 higher) | - | 124 (1 RCT) | ⨁⨁⨁◯ Moderatea | Foot orthoses likely result in little to no difference in joint range of motion compared to heel lifts in the short term. |
| Quality of life - not measured |  | |  | - | - |  |
| \***The risk in the intervention group** (and its 95% confidence interval) is based on the assumed risk in the comparison group and the **relative effect** of the intervention (and its 95% CI).    **CI:** confidence interval; **MD:** mean difference; **RR:** risk ratio | | | | | | |
| **GRADE Working Group grades of evidence**   **High certainty:** we are very confident that the true effect lies close to that of the estimate of the effect.  **Moderate certainty:** we are moderately confident in the effect estimate: the true effect is likely to be close to the estimate of the effect, but there is a possibility that it is substantially different.  **Low certainty:** our confidence in the effect estimate is limited: the true effect may be substantially different from the estimate of the effect.  **Very low certainty:** we have very little confidence in the effect estimate: the true effect is likely to be substantially different from the estimate of effect. | | | | | | |

#### Explanations

a We downgraded one level for imprecision due to one trial with results with a narrow confidence interval  
b We downgraded twice for risk of bias as this single study had a high risk of bias  
c We downgraded for publication bias due to unexplained differences in outcome measure reporting and randomisation differences between protocol and publication

d We downgraded one level for risk of bias as the study was rated as having some concerns

e We downgraded twice for imprecision due to very small participant numbers
