# Supplementary material for: Non‐surgical treatment for lower limb apophyseal injuries
Source: Cochrane Database Syst Rev. 2026 Jul 15;2026(7):CD015156. doi: 10.1002/14651858.CD015156.pub2 (PMC13370774; doi:10.1002/14651858.CD015156.pub2)
Supplement: Supplementary file 13 — Supplementary material 13 Supplementary findings tables: Heel cushioning compared to straps for children with calcaneal apophysitis for all outcomes [file CD015156-SUP-13-other.html]

Supplementary findings tables: Heel cushioning compared to straps for children with calcaneal apophysitis for all outcomes


# Supplementary material 13 to: Non-surgical treatment for lower limb apophyseal injuries

Williams CM, Krommes K, Paterson KL, Haines T, Caserta A, Thorborg K
  
https://doi.org/10.1002/14651858.CD015156.pub2

The material in this section has been supplied by the author(s) for publication under a Licence for Publication and the author(s) are solely responsible for the material. Cochrane has reviewed this material, but Cochrane has not copyedited, formatted or proofread. Cochrane accordingly gives no representations or warranties of any kind in relation to, and accepts no liability for any reliance on or use of, such material.

Back to top

# Supplementary findings tables: Heel cushioning compared to straps for children with calcaneal apophysitis for all outcomes

|  |  |  |  |  |  |  |
| --- | --- | --- | --- | --- | --- | --- |
| **Summary of findings:** | | | | | | |
| **Heel cushions compared to heel braces for children with calcaneal apophysitis** | | | | | | |
| **Patient or population:**  children with calcaneal apophysitis  **Setting:**  Tertiary care  **Intervention:**  heel cushions  **Comparison:**  heel braces | | | | | | |
| Outcomes | **Anticipated absolute effects\*** (95% CI) | | Relative effect (95% CI) | № of participants (studies) | Certainty of the evidence (GRADE) | Comments |
|  | **Risk with heel braces** | **Risk with heel cushions** |  |  |  |  |
| Overall pain - not measured | - | - | - | - | - |  |
| Physical function assessed with: OAFQ-C (Physical) Scale from: 0 to 100 follow-up: 4 weeks | The mean physical function in the short term was **78** point | MD **2 point lower**  (14.17 lower to 10.17 higher) | - | 43 (1 RCT) | ⨁◯◯◯ Very lowa,b | The evidence is very uncertain about the effect of heel cushioning on physical function in short term versus a heel strap. |
| Physical function assessed with: OAFQ-C (Physical) Scale from: 0 to 100 follow-up: 3 months | The mean physical function in the medium term was **87** points | MD **2 points higher**  (8.49 lower to 12.49 higher) | - | 43 (1 RCT) | ⨁◯◯◯ Very lowa,b | The evidence is very uncertain about the effect of heel cushioning on physical function in the medium term versus a heel strap. |
| Participation in sport or physical activity - not measured | - | - | - | - | - |  |
| Withdrawals due to adverse events - not measured | - | - | - | - | - |  |
| Adverse events | 45 per 1000 | **45 per 1000**  (3 to 665) | **RR 1.00**  (0.07 to 14.64) | 43 (1 RCT) | ⨁◯◯◯ Very lowa,b | The evidence is very uncertain about adverse events of heel cushioning compared to bracing. |
| Treatment success - not measured | - | - | - | - | - |  |
| Pain during activity assessed with: VAS Scale from: 0 to 10 follow-up: 4 weeks | The mean pain during activity in the short term was **5.5** points | MD **1.1 points higher**  (0.22 lower to 2.42 higher) | - | 43 (1 RCT) | ⨁◯◯◯ Very lowa,b | The evidence is very uncertain about the effect of heel cushioning on pain during activity in the short term versus a heel strap. |
| Pain during activity assessed with: VAS Scale from: 0 to 10 follow-up: 3 months | The mean pain during activity in the medium term was **3.6** points | MD **0.2 points lower**  (1.84 lower to 1.44 higher) | - | 43 (1 RCT) | ⨁◯◯◯ Very lowa,b | The evidence is very uncertain about the effect of heel cushioning on pain during activity in the medium term versus a heel strap. |
| Joint range of motion - not measured | - | - | - | - | - |  |
| Quality of Life - not measured | - | - | - | - | - |  |
| \***The risk in the intervention group** (and its 95% confidence interval) is based on the assumed risk in the comparison group and the **relative effect** of the intervention (and its 95% CI).    **CI:** confidence interval; **MD:** mean difference; **RR:** risk ratio | | | | | | |
| **GRADE Working Group grades of evidence**   **High certainty:** we are very confident that the true effect lies close to that of the estimate of the effect.  **Moderate certainty:** we are moderately confident in the effect estimate: the true effect is likely to be close to the estimate of the effect, but there is a possibility that it is substantially different.  **Low certainty:** our confidence in the effect estimate is limited: the true effect may be substantially different from the estimate of the effect.  **Very low certainty:** we have very little confidence in the effect estimate: the true effect is likely to be substantially different from the estimate of effect. | | | | | | |

#### Explanations

aWe downgraded twice for risk of bias as single study had a high risk of bias

bWe downgraded twice for imprecision due to very small participant numbers
