# Supplementary material for: Non‐surgical treatment for lower limb apophyseal injuries
Source: Cochrane Database Syst Rev. 2026 Jul 15;2026(7):CD015156. doi: 10.1002/14651858.CD015156.pub2 (PMC13370774; doi:10.1002/14651858.CD015156.pub2)
Supplement: Supplementary file 14 — Supplementary material 14 Supplementary summary of findings: Heel lifts compared to no treatment for children with calcaneal apophysitis [file CD015156-SUP-14-other.html]

Supplementary summary of findings: Heel lifts compared to no treatment for children with calcaneal apophysitis


# Supplementary material 14 to: Non-surgical treatment for lower limb apophyseal injuries

Williams CM, Krommes K, Paterson KL, Haines T, Caserta A, Thorborg K
  
https://doi.org/10.1002/14651858.CD015156.pub2

The material in this section has been supplied by the author(s) for publication under a Licence for Publication and the author(s) are solely responsible for the material. Cochrane has reviewed this material, but Cochrane has not copyedited, formatted or proofread. Cochrane accordingly gives no representations or warranties of any kind in relation to, and accepts no liability for any reliance on or use of, such material.

Back to top

# Supplementary summary of findings: Heel lifts compared to no treatment for children with calcaneal apophysitis

|  |  |  |  |  |  |  |
| --- | --- | --- | --- | --- | --- | --- |
| **Summary of findings:** | | | | | | |
| **Heel lifts compared to no treatment for children with calcaneal apophysitis** | | | | | | |
| **Patient or population:**  children with calcaneal apophysitis  **Setting:**  Tertiary care  **Intervention:**  heel lifts  **Comparison:**  no treatment | | | | | | |
| Outcomes | **Anticipated absolute effects\*** (95% CI) | | Relative effect (95% CI) | № of participants (studies) | Certainty of the evidence (GRADE) | Comments |
|  | **Risk with no treatment** | **Risk with heel lifts** |  |  |  |  |
| Overall pain - not measured |  |  |  |  |  |  |
| Physical function - not measured |  |  |  |  |  |  |
| Participation in sport or physical activity - not measured |  |  |  |  |  |  |
| Self reported treatment success assessed with: VAS (Higher = more satisfied) Scale from: 0 to 100 follow-up: 6 weeks | The mean self reported treatment success was **67.5** mm | MD **5 mm higher**  (3.19 lower to 13.19 higher) | - | 65 (1 RCT) | ⨁⨁◯◯ Lowa,b | Heel lifts may result in little to no difference in self reported treatment success in the short term. |
| Withdrawals due to adverse events - not measured |  |  |  |  |  |  |
| Adverse events - not measured |  |  |  |  |  |  |
| Self reported treatment success assessed with: VAS (Higher = more satisfied) Scale from: 0 to 100 follow-up: 3 months | The mean self reported treatment success was **80** mm | MD **5 mm lower**  (11.65 lower to 1.65 higher) | - | 65 (1 RCT) | ⨁⨁◯◯ Lowa,b | Heel lifts may result in little to no difference in self reported treatment success in the medium term. |
| Pain during an activity assessed with: VAS (Lower = less pain) Scale from: 0 to 10 follow-up: 6 weeks | The mean pain change during an activity was -**2.5** cm | MD **0.5 cm lower**  (1.74 lower to 0.74 higher) | - | 65 (1 RCT) | ⨁⨁◯◯ Lowa,b | Heel lifts may result in little to no difference in pain during an activity in the short term.. |
| Pain during an activity assessed with: VAS (Lower = less pain) Scale from: 0 to 10 follow-up: 3 months | The mean pain change during an activity was -**3.9** cm | MD **0.4 cm lower**  (1.79 lower to 0.99 higher) | - | 65 (1 RCT) | ⨁⨁◯◯ Lowa,b | Heel lifts may result in little to no difference in pain during an activity in the medium term. |
| Joint range of motion - not measured |  |  |  |  |  |  |
| Quality of life - not measured |  |  |  |  |  |  |
| \***The risk in the intervention group** (and its 95% confidence interval) is based on the assumed risk in the comparison group and the **relative effect** of the intervention (and its 95% CI).    **CI:** confidence interval; **MD:** mean difference | | | | | | |
| **GRADE Working Group grades of evidence**   **High certainty:** we are very confident that the true effect lies close to that of the estimate of the effect.  **Moderate certainty:** we are moderately confident in the effect estimate: the true effect is likely to be close to the estimate of the effect, but there is a possibility that it is substantially different.  **Low certainty:** our confidence in the effect estimate is limited: the true effect may be substantially different from the estimate of the effect.  **Very low certainty:** we have very little confidence in the effect estimate: the true effect is likely to be substantially different from the estimate of effect. | | | | | | |

#### Explanations

a We downgraded once for risk of bias as single study had a some concerns   
b We downgraded once for imprecision due to small participant numbers in a single trial
