# Supplementary material for: Non‐surgical treatment for lower limb apophyseal injuries
Source: Cochrane Database Syst Rev. 2026 Jul 15;2026(7):CD015156. doi: 10.1002/14651858.CD015156.pub2 (PMC13370774; doi:10.1002/14651858.CD015156.pub2)
Supplement: Supplementary file 17 — Supplementary material 17 Supplementary summary of findings: Exercise compared to heel lifts for children with calcaneal apophysitis [file CD015156-SUP-17-other.html]

Supplementary summary of findings: Exercise compared to heel lifts for children with calcaneal apophysitis


# Supplementary material 17 to: Non-surgical treatment for lower limb apophyseal injuries

Williams CM, Krommes K, Paterson KL, Haines T, Caserta A, Thorborg K
  
https://doi.org/10.1002/14651858.CD015156.pub2

The material in this section has been supplied by the author(s) for publication under a Licence for Publication and the author(s) are solely responsible for the material. Cochrane has reviewed this material, but Cochrane has not copyedited, formatted or proofread. Cochrane accordingly gives no representations or warranties of any kind in relation to, and accepts no liability for any reliance on or use of, such material.

Back to top

# Supplementary summary of findings: Exercise compared to heel lifts for children with calcaneal apophysitis

|  |  |  |  |  |  |  |
| --- | --- | --- | --- | --- | --- | --- |
| **Summary of findings:** | | | | | | |
| **Exercise compared to heel lifts for children with calcaneal apophysitis** | | | | | | |
| **Patient or population:**  children with calcaneal apophysitis  **Setting:**  Tertiary care  **Intervention:**  exercise  **Comparison:**  heel lifts | | | | | | |
| Outcomes | **Anticipated absolute effects\*** (95% CI) | | Relative effect (95% CI) | № of participants (studies) | Certainty of the evidence (GRADE) | Comments |
|  | **Risk with heel lifts** | **Risk with exercise** |  |  |  |  |
| Overall pain - not measured |  |  |  |  |  |  |
| Physical function - not measured |  |  |  |  |  |  |
| Participation in sport or physical activity - not measured |  |  |  |  |  |  |
| Adverse events - not reported |  |  |  |  |  |  |
| Withdrawals due to adverse events - not reported |  |  |  |  |  |  |
| Treatment success assessed with: VAS (Higher = more satisfied) Scale from: 0 to 100 follow-up: 6 weeks | The mediun self reported treatment success in the short term was **80** mm | MD **6.25 mm lower**  (14.66 lower to 2.16 higher) | - | 66 (1 RCT) | ⨁⨁◯◯ Lowa,b | Exercise may result in little to no difference in self reported treatment success in the short term compared to heel lifts. |
| Treatment success assessed with: VAS (Higher = more satisfied) Scale from: 0 to 100 follow-up: 3 months | The median self reported treatment success in the medium term was **85** mm | MD **2.5 mm higher**  (4.56 lower to 9.56 higher) | - | 66 (1 RCT) | ⨁⨁◯◯ Lowa,b | Exercise may result in little to no difference in self reported treatment success in the medium term compared to heel lifts. |
| Pain during activity assessed with: FPS-R Scale from: 0 to 10 follow-up: 6 weeks | The mean pain during activity in the short term was **-2.5** cm | MD **0.7 cm lower**  (1.88 lower to 0.48 higher) | - | 66 (1 RCT) | ⨁⨁◯◯ Lowa,b | Exercise may result in little to no difference in pain during activity in the short term compared to heel lifts. |
| Pain during activity assessed with: FPS-R Scale from: 0 to 10 follow-up: 3 months | The mean pain during activity in the medium term was **-4.3** cm | MD **0.5 cm higher**  (0.92 lower to 1.92 higher) | - | 66 (1 RCT) | ⨁⨁◯◯ Lowa,b | Exercise may result in little to no difference in pain during activity in the medium term compared to heel lifts. |
| Joint range of motion - not measured |  |  |  |  |  |  |
| Quality of life - not measured |  |  |  |  |  |  |
| \***The risk in the intervention group** (and its 95% confidence interval) is based on the assumed risk in the comparison group and the **relative effect** of the intervention (and its 95% CI).    **CI:** confidence interval; **MD:** mean difference | | | | | | |
| **GRADE Working Group grades of evidence**   **High certainty:** we are very confident that the true effect lies close to that of the estimate of the effect.  **Moderate certainty:** we are moderately confident in the effect estimate: the true effect is likely to be close to the estimate of the effect, but there is a possibility that it is substantially different.  **Low certainty:** our confidence in the effect estimate is limited: the true effect may be substantially different from the estimate of the effect.  **Very low certainty:** we have very little confidence in the effect estimate: the true effect is likely to be substantially different from the estimate of effect. | | | | | | |

#### Explanations

a We downgraded once for risk of bias as single study had a some concerns   
b We downgraded once for imprecision due to small participant numbers in a single trial
